# Dnmt1 has de novo activity targeted to transposable elements

**DOI:** 10.1038/s41594-021-00603-8

**Published:** 2021-06-17

**Authors:** Chuck Haggerty, Helene Kretzmer, Christina Riemenschneider, Abhishek Sampath Kumar, Alexandra L. Mattei, Nina Bailly, Judith Gottfreund, Pay Giesselmann, Raha Weigert, Björn Brändl, Pascal Giehr, René Buschow, Christina Galonska, Ferdinand von Meyenn, Melissa B. Pappalardi, Michael T. McCabe, Lars Wittler, Claudia Giesecke-Thiel, Thorsten Mielke, David Meierhofer, Bernd Timmermann, Franz-Josef Müller, Jörn Walter, Alexander Meissner

**Affiliations:** 1 Department of Genome Regulation, Max Planck Institute for Molecular Genetics, Berlin, Germany; 2 Institute of Chemistry and Biochemistry, Freie Universität Berlin, Berlin, Germany; 3 Institute of Biotechnology, Technische Universität Berlin, Berlin, Germany; 4 Department of Stem Cell and Regenerative Biology, Harvard University, Cambridge, MA USA; 5 Department of Molecular and Cellular Biology, Harvard University, Cambridge, MA USA; 6 Department of Genetics and Epigenetics, Saarland University, Saarbrücken, Germany; 7 Christian-Albrechts-Universität zu Kiel, Department of Psychiatry and Psychotherapy, Kiel, Germany; 8 Institute of Food, Nutrition and Health, ETH Zurich, Schwerzenbach, Switzerland; 9 Microscopy and Cryo-electron Microscopy Service Group, Max Planck Institute for Molecular Genetics, Berlin, Germany; 10 Spatial Transcriptomics, Part of 10x Genomics Inc, Stockholm, Sweden; 11 Epigenetics Research Unit, Oncology R&D, GlaxoSmithKline, Collegeville, PA USA; 12 Department of Developmental Genetics, Max Planck Institute for Molecular Genetics, Berlin, Germany; 13 Flow Cytometry Joint Facilities Scientific Service, Max Planck Institute for Molecular Genetics, Berlin, Germany; 14 Mass Spectrometry Joint Facilities Scientific Service, Max Planck Institute for Molecular Genetics, Berlin, Germany; 15 Sequencing Core Facility, Max Planck Institute for Molecular Genetics, Berlin, Germany; 16 Broad Institute of MIT and Harvard, Cambridge, MA USA

**Keywords:** Epigenomics, Chromatin, Developmental biology, DNA methylation

## Abstract

DNA methylation plays a critical role during development, particularly in repressing retrotransposons. The mammalian methylation landscape is dependent on the combined activities of the canonical maintenance enzyme Dnmt1 and the de novo Dnmts, 3a and 3b. Here, we demonstrate that Dnmt1 displays de novo methylation activity in vitro and in vivo with specific retrotransposon targeting. We used whole-genome bisulfite and long-read Nanopore sequencing in genetically engineered methylation-depleted mouse embryonic stem cells to provide an in-depth assessment and quantification of this activity. Utilizing additional knockout lines and molecular characterization, we show that the de novo methylation activity of Dnmt1 depends on Uhrf1, and its genomic recruitment overlaps with regions that enrich for Uhrf1, Trim28 and H3K9 trimethylation. Our data demonstrate that Dnmt1 can catalyze DNA methylation in both a de novo and maintenance context, especially at retrotransposons, where this mechanism may provide additional stability for long-term repression and epigenetic propagation throughout development.

## Abstract (web summary)

The canonical DNA methylation maintenance enzyme Dnmt1 displays global de novo methylation activity with greater targeting towards IAP transposons, which may contribute to their stable repression during early development.

## Main

Mammalian development is a highly orchestrated process facilitated by a complex multi-layered epigenome that dynamically changes as a function of time. After fertilization, the genome undergoes global DNA demethylation, followed by an extensive wave of de novo methylation to establish the somatic methylation landscape. This is dependent on the combined activities of the maintenance DNA methyltransferase (Dnmt) 1, its co-factor the E3 ubiquitin protein ligase Uhrf1, and the de novo enzymes Dnmt3a and Dnmt3b. Through their activity, Dnmts affect genomic stability and accessibility, including the silencing of transposable elements^1–3^. Loss of Dnmt1 is embryonically lethal and leads to a global reduction of DNA methylation that results in increased expression of long terminal repeat (LTR) retrotransposons, most notably intracisternal A particles (IAPs)^3–5^.

Currently, Dnmt1 is described as the canonical maintenance methyltransferase because of its high affinity towards hemimethylated DNA and its role in re-establishing the methylation landscape after DNA replication^6,7^, while the de novo Dnmts (3a/3b) target CpG sites in both hemi- and unmethylated contexts^8^. Dnmt1’s preference towards hemimethylated substrates has been linked to its auto-inhibitory conformation and presumed inability to act alone. Uhrf1 appears necessary for alleviating this inhibition and also plays a role in the localization as well as genomic recruitment of Dnmt1^9–12^.

Despite Dnmt1’s strong preference for hemimethylated DNA substrate, some studies have reported that it can, albeit with low efficiency, catalytically act on unmethylated DNA substrates. For example, purified Dnmt1 has been shown to have up to 50-fold higher activity towards hemimethylated DNA, but nonetheless catalyzed measurable methylation of unmethylated DNA substrates^13,14^. Mouse embryonic stem cells (ESCs) deficient for both Dnmt3a and 3b were shown to gain low levels of methylation (<3%) at an integrated unmethylated Moloney murine leukemia virus LTR and slightly higher levels when introduced with some pre-existing methylation^15^. Hidden Markov models of Dnmt activity based on repeat elements analyzed using hairpin-bisulfite sequencing predicted Dnmt1 to have methylation activity at IAPs and major satellite repeats^16^. It was also suggested that Dnmt1 has a role in the de novo methylation of inactive gene promoters in *Stella* (*Dppa3*) knockout oocytes. Specifically, a low-level gain of methylation for Stella and Dnmt3a deficient oocytes was reported compared to Stella and Dnmt1 knockouts. However, the presence of other Dnmts, such as Dnmt3b, and the inability to separate Dnmt1 maintenance and de novo methylation activity complicate the interpretation^17^. Finally, two recent studies investigating DNA replication-coupled maintenance using a combination of DNA labeling and hairpin-bisulfite sequencing suggested the possibility of Dnmt1 engaging in low levels of post-replication de novo methylation^18,19^. Based on these earlier studies, which all make intriguing observations, a thorough and well-controlled investigation is needed to clarify the precise ability of Dnmt1 to act as a de novo enzyme in cells and development.

## Results

### In vivo gain of DNA methylation in the absence of Dnmt3s

**Fig. 1:**
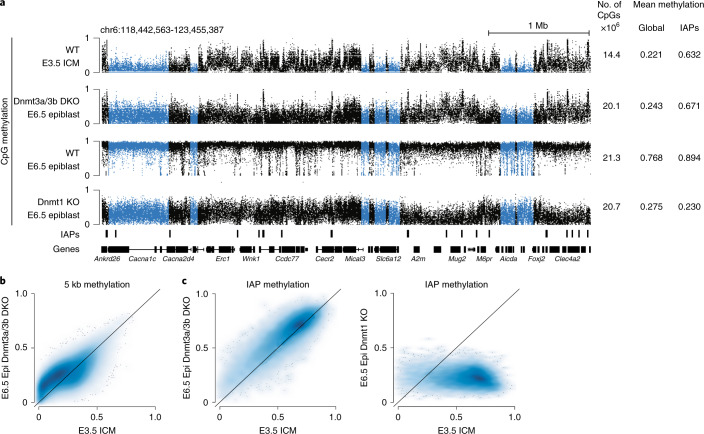
Dnmt1 displays de novo methylation activity in vivo. **a**, Representative genome browser tracks of WGBS data for WT E3.5 ICM (*n* = 2 samples from 5–10 pooled embryos each), Dnmt3a/3b DKO E6.5 epiblast (*n* = 1 from ~10 pooled embryos each), WT E6.5 epiblast (*n* = 2 from ~10 pooled embryos each) and Dnmt1 KO E6.5 epiblast (*n* = 1 from ~10 pooled embryos each). Regions with a notable gain of methylation in the absence of the de novo Dnmt3s are highlighted across all tracks in blue. The coverage and average methylation values are shown on the right. **b**, Correlation plot of E3.5 ICM compared to E6.5 epiblast (Epi) of the Dnmt3a/3b DKO in 5-kb windows, excluding all IAP retrotransposon overlapping CpGs. *n* = 461,268 5-kb windows. **c**, Correlation plot of the average IAP retrotransposon methylation per element in the E3.5 ICM compared to E6.5 epiblast of the Dnmt3a/3b DKO (left) and Dnmt1 KO (right); 48% of IAPs are more methylated (>0.05 methylation difference) than in the ICM of Dnmt3a/3b DKO epiblast and almost all (96%) are less methylated in the Dnmt1 KO than in the ICM. *n* = 14,787 IAP elements. Source data are provided at 10.6084/m9.figshare.14555250.

To begin this investigation, we compared the global landscape of wild-type (WT) embryonic day (E) 3.5 inner cell mass (ICM), which represents a low point of DNA methylation during early development, with Dnmt3a/3b double knockout (DKO) post-implantation E6.5 epiblast, a stage where re-methylation is largely completed in wild-type embryos (Fig. 1a)^20^. Unexpectedly, we found that 50% of the genome gains more than 5% methylation, even in the absence of the Dnmt3s, compared to the ICM, pointing towards a rather widespread Dnmt3-independent de novo methylation activity (Fig. 1a,b, blue shaded areas). Another noteworthy feature of the Dnmt3a/3b DKO epiblast methylome is the highly methylated regions overlapping with IAPs (Fig. 1a,c, left). By contrast, the Dnmt1 KO epiblast loses DNA methylation at IAPs, with 96% of IAPs being less methylated in the E6.5 Dnmt1 KO versus E3.5 ICM (methylation loss is defined as a difference greater than 5% (delta ≥ 0.05); Fig. 1a,c, right). This highlights that Dnmt1, not Dnmt3 activity, is necessary and sufficient to maintain high methylation levels at IAPs through pre- and post-implantation development.

### DNA-methylation-depleted ESCs confirm Dnmt1 de novo activity

**Fig. 2:**
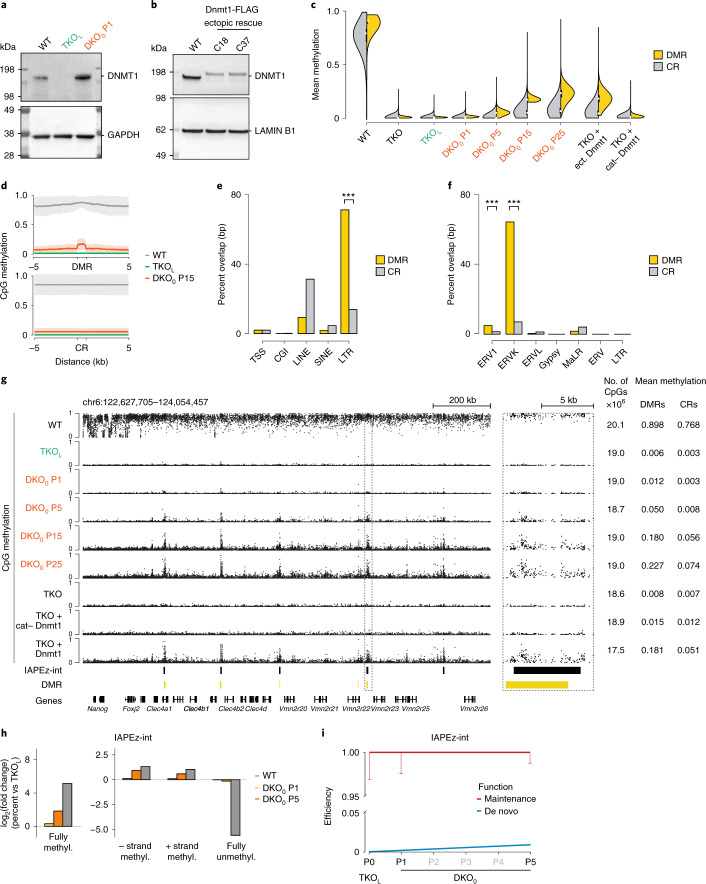
Dnmt1 de novo activity is targeted towards IAP elements in vitro. **a**, Western blot of DNMT1 (183 kDa) in WT, TKO_L_ and DKO_0_ ESCs. Loading control: GAPDH (36 kDa). **b**, Western blot of DNMT1 in WT and TKO+ ectopic FLAG-tagged Dnmt1. Ectopic DNMT1 protein runs higher due to the addition of the FLAG tag. Loading control: LAMIN B1 (62 kDa). **c**, Methylation distribution of DMRs (yellow) and CRs (gray) for WT, TKO, TKO_L_, DKO_0_ at P1, P5, P15 and P25, TKO+ ectopic Dnmt1 and TKO + catalytically inactive Dnmt1 (cat−), one sample each. Average methylation, from left to right: DMRs, 0.898, 0.008, 0.006, 0.012, 0.050, 0.180, 0.227, 0.181, 0.015; CRs, 0.768, 0.007, 0.003, 0.003, 0.008, 0.056, 0.074, 0.051, 0.012. The boxplot elements are defined as follows: white dot, median; boxes, first and third quartiles; whiskers, 1.5× inter-quartile range; data beyond the whiskers are omitted. CGI, CpG island; LINE, long interspersed nuclear element; SINE, short interspersed nuclear element. **d**, Profile plot depicting the methylation over DMRs or CRs in WT, TKO_L_ and DKO_0_ at P15 (*n* = 2,573 DMRs and 2,573,000 CRs). **e**, Distribution of DMRs across genomic regions. The percent overlap in length is measured in base pairs. ***P = 2.2 × 10^−16^, Wilcoxon test, one-sided. **f**, Distribution of DMRs across LTR retrotransposon classes; ***P < 0.001, ERV1 = 2.49 × 10^−5^, ERVK = 2.2 × 10^−16^, Wilcoxon test, one-sided. **g**, Methylation tracks for WT, TKO_L_ and DKO_0_ at P1, P5, P15 and P25, as well as the TKO, TKO + catalytically inactive Dnmt1 (cat−) and TKO + ectopic Dnmt1. Right: zoom-in to a representative DMR (dashed box). Total number of CpGs measured and average methylation values are shown. **h**, Hairpin-bisulfite data depicted as log_2_(fold change) of all possible CpG dyad combinations at IAPEz-int elements (±strand hemimethylation, fully methylated or unmethylated on complementary strands) for WT, P1 and P5 DKO_0_ CpG versus TKO_L_. **i**, Visualization of maintenance and de novo efficiencies predicted by a hidden Markov model using IAPEz-int hairpin-bisulfite sequencing data from TKO_L_, P1 and P5 DKO_0_. Black labels indicate the sampled time points. Uncropped images for **a** and **b** are available as source data. Additional source data are provided at 10.6084/m9.figshare.14555250. Source data

**Extended Data Fig. 1:**
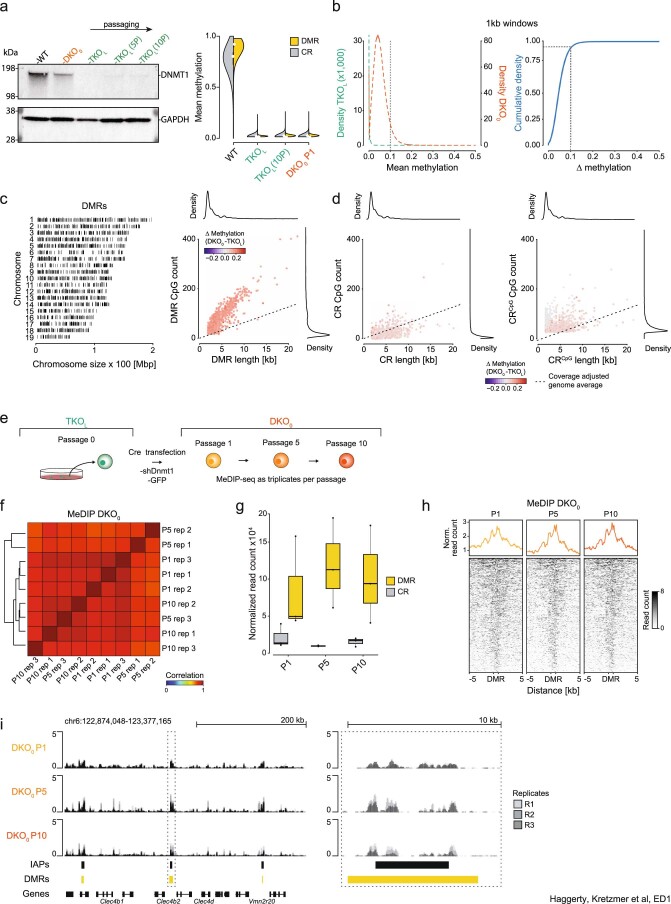
Focal gain of methylation depends on Dnmt1, is stable, and reproducible. **a**, Western blot of DNMT1 (183kDa) in WT, DKO_0_ and TKO_L_ passage 1, 5 and 10. Loading control: GAPDH (36kDa). *n* = 1. Violin plot showing average methylation distribution of DMRs (yellow) and CRs (gray) for WT, TKO_L_ P1, TKO_L_ P10, and DKO_0_ P1. White dots indicate the median; boxes indicate first and third quartiles; whiskers, 1.5 x inter-quartile range; data beyond the end of the whiskers are omitted. **b**, Genome-wide 1kb window methylation rate distribution of TKO_L_ and DKO_0_ P15 cells (left). The empirical cumulative distribution curve of the TKO_L_ and DKO_0_ methylation difference in 1kb windows (right). Dotted line shows the 95% quantile. **c**, Genomic distribution of DMRs (black lines, *n* = 2,573). The height of the black line indicates the mean methylation difference of the DMRs (left). DMR and length in base pairs versus CpG count. Methylation difference is indicated by dot color, and the dashed line indicates the coverage adjusted genomic average (right). **d**, CR (random: light gray, CpG matched: dark gray) length in base pairs versus CpG count. Methylation difference is indicated by dot color and the dashed line indicates the coverage adjusted genomic average. **e**, Simplified overview of the MeDIP-seq experiment and sample collection. **f**, Correlation-based clustering of MeDIP-seq replicates for passage 1, 5, and 10 (*n* = 3 each). The color indicates the correlation value (Spearman correlation). **g**, Read-counts at DMRs and CRs normalized for the number of regions. Centerline is median; boxes, first and third quartiles; whiskers, 1.5 x inter-quartile range; points are data beyond the end of whiskers. **h**, Summary plots of aggregated replicates for each of the three passages over DMRs. **i**, Representative genome browser track showing MeDIP-seq coverage for each replicate (different gray scale). Source data

**Extended Data Fig. 2:**
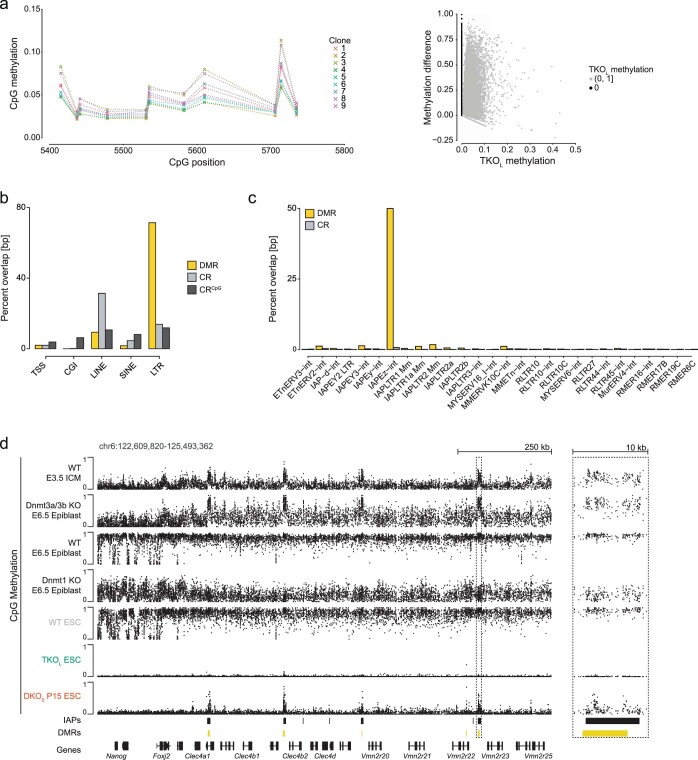
DMRs are enriched for IAPEz-int sub-family of ERVK LTR retrotransposons. **a**, Methylation levels of CpGs in IAPEz-gag as measured by bisulfite amplicon sequencing. Clones are single cell sorted using FACS from DKO_0_ P1 and then passaged until P5 as individual clones. Strong similarities of overall methylation level and pattern across clones was observed (left). Comparison of TKO_L_ methylation levels and gain of methylation between TKO_L_ and P15 in the DKO_0_ for position matched CpGs. Completely unmethylated CpGs (black, defined as 0.00) gain methylation to a similar extent as CpGs with low-level methylation in the TKO_L_. **b**, Distribution of DMRs, CRs and CRs^CpG^ across genomic regions. DMRs overlap with LTR retrotransposon at a significantly greater frequency than both CRs. Despite similar CpG density of DMRs and CRs^CpG^, only CRs^CpG^ show enrichment in CpG rich regions like CGI and a depletion in LTR overlap compared to CRs. Percent overlap in length measured in base pairs. **c**, Distribution of DMRs within ERVK LTR subclasses. DMRs overlap most frequently with the IAPEz-int sub-family of ERVK LTR retrotransposons. CRs do not show any strong bias across ERV families. Percent overlap in length is measured in base pairs. **d**, Representative genome browser tracks of *in vivo* and *in vitro* DNA methylation data. Methylation values per CpG (dot) in WT E3.5 ICM, Dnmt3a/3b DKO, E6.5 WT, and Dnmt1 KO Epiblast (top) compared to WT, TKO_L,_ and DKO_0_ P15 ESC lines (bottom). DMRs between TKO_L_ and DKO_0_ P15 overlap with IAP elements and are hypermethylated compared to background regions in the ICM and Dnmt3a/3b Epiblast. Notably, this higher methylation level is specifically missing in the Dnmt1 KO Epiblast. Dashed box is a zoom-in to highlight the levels over a representative DMR.

To more systematically investigate the Dnmt3-independent de novo activity, we utilized mouse ESCs that lack catalytically active Dnmt3a and Dnmt3b, combined with reversible Dnmt1 depletion, using a Cre-excisable short-hairpin RNA (shRNA) against Dnmt1 (triple knockout like, TKO_L_; Fig. 2a, Extended Data Fig. 1a and Supplementary Table 1). This system shows global loss of DNA methylation and enables us to track any de novo activity upon knockdown reversal using endogenous Dnmt1 (termed double knockout zero methylation, DKO_0_; Fig. 2a)^21,22^. Importantly, the clonal nature of the TKO_L_ cell line excludes that a subpopulation of cells retained methylation and then later expanded to give rise to the observed changes in methylation. However, to further remove any uncertainty, we also created a true triple knockout (TKO) ESC line and later rescued Dnmt1 through ectopic expression via PiggyBac integration (Fig. 2b). We then generated whole-genome bisulfite sequencing (WGBS) data of our ESC lines, including a time course of DKO_0_ after 1, 5, 15 and 25 passages (P). Both the endogenous rescue and ectopic expression show a notable gain of Dnmt1-induced de novo DNA methylation (Fig. 2c). Importantly, the vector-based ectopic reintroduction of Dnmt1 into the TKO cells further demonstrates that Dnmt1’s catalytic function is necessary, as we did not observe a gain of methylation when introducing a catalytically inactive Dnmt1 mutant. Next, we determined differentially methylated regions (DMRs) between the TKO_L_, from which all DKO_0_ samples are generated, and DKO_0_ at P15 (DMRs, *n* = 2,573, Supplementary Table 2). The latter was chosen for the DMR calling as it shows a robust gain of methylation at specific regions over background. We also defined a set of length-matched, randomly distributed control regions for our analysis (CRs; *n* = DMRs × 1,000; Extended Data Fig. 1b–d and Supplementary Information). The DMRs have a mean methylation of 0.180 in the DKO_0_ at P15 (compared to 0.006 in TKO_L_), while the gain at CRs matches the global average (Fig. 2c,d). Thus, the methylation increase seems to segregate into two distinct activities: an ubiquitous lower-level global gain and a more pronounced gain at specific focal regions. To independently confirm the DMRs and assess their consistent emergence, we used methylated DNA immunoprecipitation sequencing (MeDIP-seq) on isolated and passaged clones of DKO_0_ P1, P5 and P10 in triplicate. Although MeDIP is not as quantitative, it provides a binary estimate regarding the presence or absence of methylation and shows that Dnmt1 re-expression leads to a highly reproducible gain of methylation across replicates (Extended Data Fig. 1e–i). Finally, we used fluorescence-activated single-cell sorting to isolate individual cells from DKO_0_ at P1 and expanded individual clones until P5. We then assayed methylation at IAPEz-int (internal region of an IAP-subtype) elements with amplicon bisulfite sequencing and found little variation and similar methylation patterns emerging within the nine tested clones. Furthermore, we compared the methylation levels of individual CpGs within DMRs between the TKO_L_ to the gain between the TKO_L_ and DKO_0_ P15. Notably, CpGs with residual levels of methylation in the TKO_L_ did not show any larger gain in the DKO_0_ than fully unmethylated CpGs. Together with the clonal origin of the TKO_L_ cell line, these results argue against remaining methylation in a subpopulation of cells as the explanation for our observed de novo methylation (Extended Data Fig. 2a).

### IAPs are specific targets of Dnmt1’s de novo activity

**Extended Data Fig. 3:**
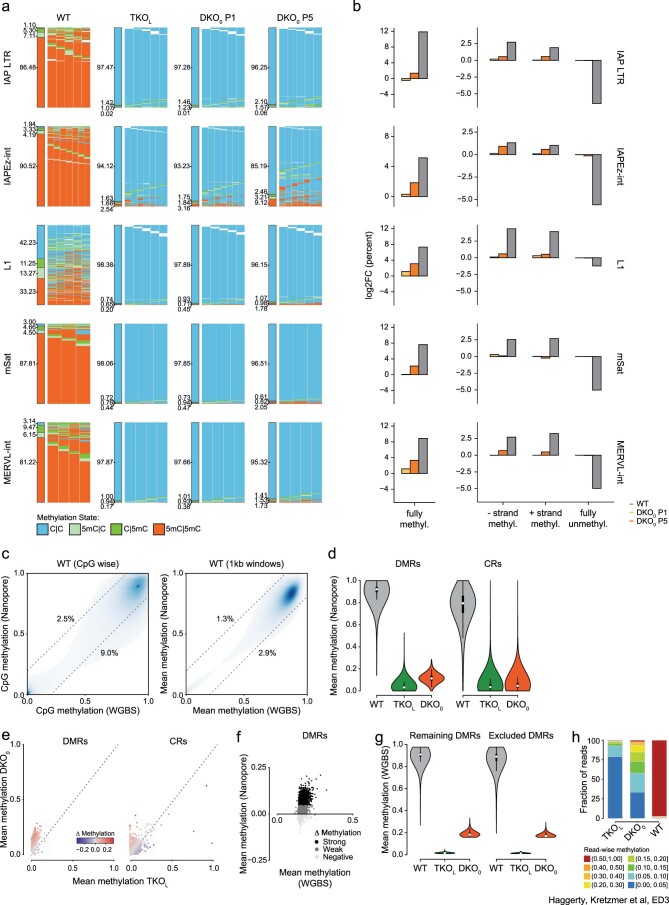
Generation of Uhrf1 KO TKO_L_ clones and enrichment of H3K9me3 at DMRs. **a**, Amplicon hairpin-bisulfite sequencing data for several repeat classes in WT, TKO_L_, P1, and P5 DKO_0_ with columns representing covered CpGs, except the leftmost (average) and rows individual reads. Light blue represents an unmethylated CpG dyad, orange a fully methylated one, and dark and light green represent plus or minus-strand hemimethylation. **b**, The same amplicon data from panel a depicted as log2 fold-change of all CpG dyad combinations for WT, P1, and P5 DKO_0_ CpG versus TKO_L_. **c**, Correlation of DNA methylation rates in WT measured by WGBS and Nanopore at CpG resolution and over 1kb windows. **d**, DNA methylation distribution based on Nanopore sequencing. Shown are mean methylation levels of DMRs (left *n* = 1,335) and CRs (right, *n* = 1,268,188) in WT, TKO_L,_ and DKO_0_ ESC. For violin plots, white dots indicate the median, the boxes indicate first and third quartiles; whiskers, 1.5 x inter-quartile range; data beyond the end of the whiskers are omitted. **e**, Comparison of mean DMR methylation levels of TKO_L_ vs. DKO_0_ cells and mean CR methylation levels of TKO_L_ vs. DKO_0_ cells. **f**, Comparison of WGBS and Nanopore methylation levels. DMRs with a difference below 0.05 (Nanopore), were discarded. Gray dots represent unsupported, filtered DMRs with a mean difference of less than 0.05 (*n* = 1,058). **g**, Methylation distribution (WGBS) of supported (remaining) and unsupported (excluded) DMRs (*n* = 1,515 and 1,058, respectively). For violin plots, white dots indicate the median, the boxes indicate first and third quartiles; whiskers, 1.5 x inter-quartile range; data beyond the end of the whiskers are omitted. **h**, Fraction of Nanopore single-read methylation levels in DMRs with at least 5 CpGs and spanning a minimum of 80% of the DMRs for WT, TKO_L_ and DKO_0_.

Next, we compared the DMR distribution over genomic features and found a highly significant enrichment at LTRs, which is independent of regional CpG density (*P* < 2.2 × 10^−16^, Wilcoxon test, DMRs compared with CRs; Fig. 2e and Extended Data Fig. 2b). LTRs contain several families, including ERV1 and ERVK, which were specifically enriched in DMRs relative to CRs (*P*_ERV1_ = 2.49 × 10^−05^, *P*_ERVK_ < 2.2 × 10^−16^, Wilcoxon test; Fig. 2f). More precisely, the DMRs overlap with the IAPEz-int sub-family of ERVKs and gain methylation over time (Fig. 2g and Extended Data Fig. 2c). As noted above, IAP methylation is maintained at high levels through early development in WT as well as Dnmt3-deficient embryos; Dnmt1 de novo DMRs in ESCs show substantial overlap with the regions of focal methylation in the ICM and the Dnmt3a/3b DKO epiblast (Extended Data Fig. 2d). To further explore Dnmt1’s de novo activity on the complementary DNA strands and model the kinetics of this process, we subjected WT, TKO_L_, as well as P1 and P5 DKO_0_ cells to hairpin-bisulfite amplicon sequencing across several repeat classes. We observed an increase in fully methylated CpG dyads across all repeat classes relative to the TKO_L_ by P5, with IAPEz showing the highest fraction (Extended Data Fig. 2h). Additionally, in contrast to other repeat classes, the IAPEz elements show a relative increase in hemimethylated CpG dyads on both strands across passaging (Fig. 2h and Extended Data Fig. 3b). This increase in hemimethylated DNA, specifically at full-length IAPEz elements, over other repeat classes (including the larger set of IAP LTRs that includes more than 4,000 solo LTRs) could potentially be the result of sequence-specific features that influence Dnmt1’s recruitment and hence its de novo activity. We then used the H(O)TA hidden Markov model to estimate the efficiency of de novo and maintenance methylation^23^. For most repeat classes, the low levels of methylation in the KO lines make modeling difficult; however, the increased methylation observed at IAPEz-int elements allowed us to effectively generate a model of de novo versus maintenance methylation (Fig. 2i). Taken together, this points to a measurable de novo activity of Dnmt1, in particular at IAPs, that together with its canonical and highly efficient maintenance activity ensures robust methylation levels, independent of the Dnmt3s.

### Nanopore sequencing enables unique mapping and DMR calling

Approximately 40% of the mouse genome is composed of repetitive sequences, and each strain usually shows some deviation from the standard reference genome. Consequently, reads generated from short-read techniques can align to multiple loci or regions missing in the actual sequenced genome. To exclude potential sequencing or alignment artifacts, ensure our findings are not biased by ambiguous short-read alignments, or overemphasize missing sites, we utilized long-read sequencing (Oxford Nanopore Technologies). This technology detects methylation, independent of bisulfite conversion, and can readily produce reads longer than 25 kb, which allows for their precise placement in the genome, including reliable detection of genomic rearrangements and therefore accurate locus-specific methylation calling.

**Fig. 3:**
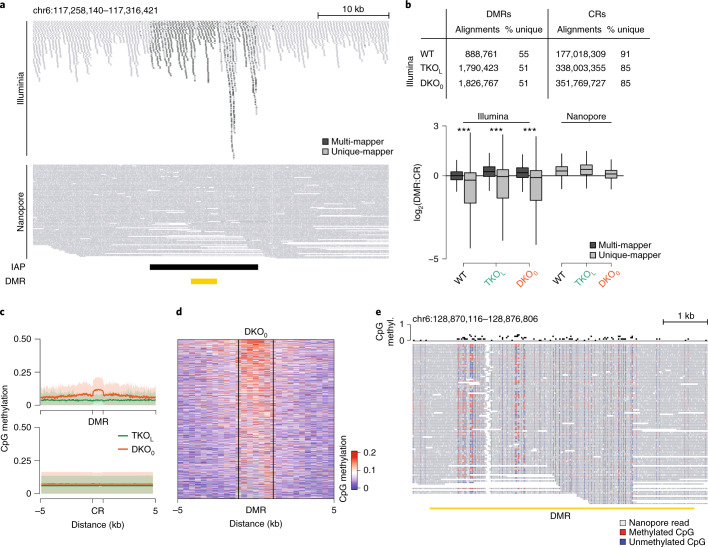
Nanopore sequencing enables high-confidence DMR calling. **a**, Genome browser tracks displaying alignments of Illumina short-read (top) and Nanopore long-read (bottom) sequencing data over a representative DMR region. Owing to the overlap of DMRs with repetitive sequences, DMRs show a local enrichment of multi-mapped Illumina short-reads (black). Conversely, Nanopore long-reads homogeneously cover the DMR using only unique mappers. **b**, Top: summary of alignment statistics of Illumina short-reads and Nanopore long-reads in DMRs and CRs. Short-read alignments show a local enrichment in multi-mappers in DMRs. Bottom: boxplots showing the distribution of DMR coverage (*n* = 2,573 DMRs) normalized to corresponding CR coverage for Illumina (left) and Nanopore (right). Removal of multi-mappers from Illumina reads significantly decreases DMR coverage compared to CR coverage (***P < 0.001, Illumina 2.2 × 10^−16^, Wilcoxon test, paired, two-sided). Nanopore sequencing depth: WT 62×, TKO_L_ 91×, and DKO_0_ 93×. For boxplots, the centerline is median; boxes, first and third quartiles; whiskers, 1.5× inter-quartile range; outlier data points are omitted. **c**, Profile plot depicting the methylation distribution measured by Nanopore sequencing over DMRs and CRs in TKO_L_ and DKO_0_. **d**, Heatmap displaying DKO_0_ methylation levels across DMRs. Nanopore sequencing-derived methylation levels at DMRs with a minimum of 10 sufficiently covered CpGs show methylation levels above background across the entire DMR. **e**, Representative genome browser track of DKO_0_ reads aligned to a DMR region. Reads span large portions of the DMR and show both methylated (red) as well as unmethylated (blue) CpGs within a single read. Source data are provided at 10.6084/m9.figshare.14555250.

We sequenced the WT, TKO_L_ and DKO_0_ P15 to an average of 82× genome-wide coverage considering only uniquely aligned reads (Fig. 3a,b). In contrast to the short-read data with a high multi-mapper fraction in DMRs, we did not observe a coverage bias at DMRs versus CRs with the Nanopore data. We then utilized these long reads in two ways: first, we directly excluded all CpGs that are annotated in regions detected as a deletion in the TKO_L_ and DKO_0_ but present in the reference genome from the DMR detection (*n* = 8,210 missing regions, in total 26 Mb, excluded from the analysis). Second, we filtered the WGBS-derived DMRs for high confidence by excluding those with an average methylation difference of less than 0.05 between the DKO_0_ and TKO_L_ in the Nanopore data (*n* = 1,058 excluded DMRs, Extended Data Fig. 3c–g, Supplementary Information). This filtering strategy resulted in 1,515 remaining high-confidence DMRs with a focal gain in methylation as detected by WGBS and, if covered, Nanopore sequencing (Fig. 3c and Extended Data Fig. 3g). Given that these long reads span much larger regions, we could show the gain of methylation on single molecules across the entire length of the DMRs (Fig. 3d,e and Extended Data Fig. 3h). These measurements allowed us to increase the accuracy of our DMRs in a manner unbiased by short-read mapping ability at repetitive sequences, account for genomic differences to the reference and filter for highly methylated DMRs.

### Dnmt1 requires Uhrf1 for de novo methylation

**Fig. 4:**
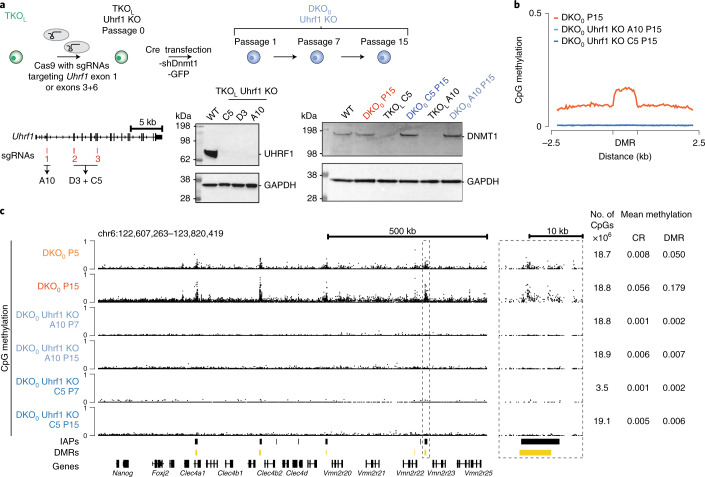
Uhrf1 is essential for Dnmt1’s de novo function. **a**, Overview of the experimental design for CRISPR-Cas-mediated Uhrf1 deletion in TKO_L_ cells and the derivation of DKO_0_ cells in the absence of Uhrf1. Targets for the sgRNAs are indicated and the names of the isolated clonal lines are shown below. Western blot analysis of UHRF1 (95 kDa) in WT ESC and *Uhrf1* KO TKO_L_ clones C5, D3 and A10 (left) and of DNMT1 (183 kDa) protein levels in the various cell lines (right). GAPDH (36 kDa) serves as the loading control. **b**, Profile plot of the methylation level over DMRs in DKO_0_ P15 compared to two Uhrf1 KO DKO_0_ clones at P15. **c**, Representative genome browser tracks of WGBS data for DKO_0_ P5 and P15 and the two Uhrf1 KO DKO_0_ clones at P7 and P15. Right: zoom-in to a representative DMR (dashed box). Total number of CpGs measured and average methylation values are shown. Source data

**Extended Data Fig. 4:**
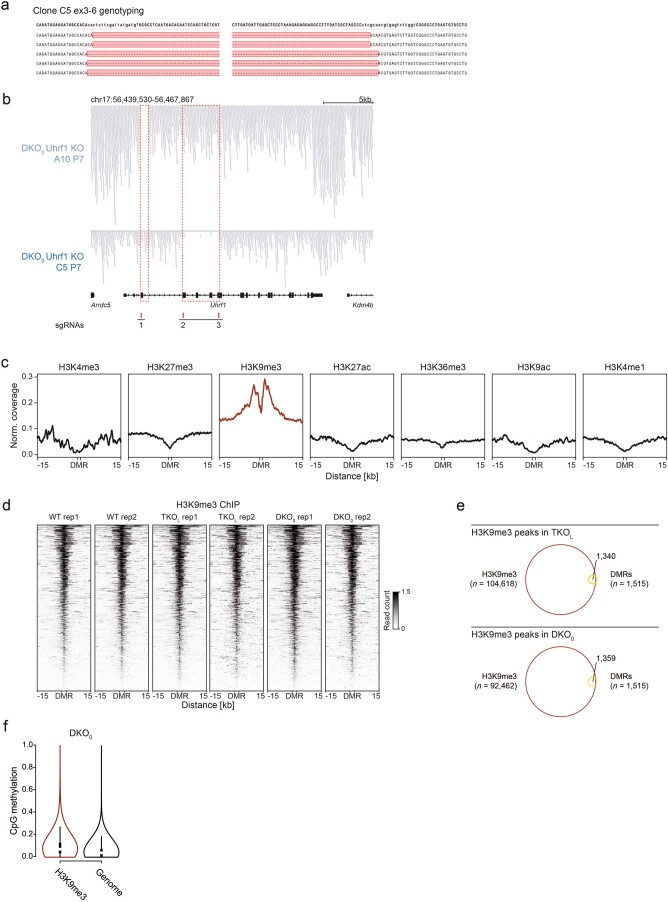
Generation of Uhrf1 KO TKO_L_ clones and enrichment of H3K9me3 at DMRs. **a**, Sanger sequencing result for clone C5 indicates the deletion of exons 3–6 and a resulting frameshift mutation. **b**, Confirmation of *Uhrf1* disruption in DKO_0_ clones. WGBS reads were used to infer *Uhrf1* gene targeting in P7 Uhrf1 KO DKO_0_ clones A10 (top) and C5 (bottom). Read alignments show large deletions proximal to sgRNA target sites. Clone A10 has a deletion of the initiation codon in the first protein-coding exon, and Clone C5 has a large exon 3–6 deletion with a resulting frameshift. **c**, Coverage of Encode histone ChIP-seq from Bruce-4 ESCs over DMRs. Only H3K9me3 ChIP-seq data show increased enrichment around the DMRs, while all other included histone modifications appear depleted compared to their background. **d**, Heatmap of H3K9me3 enrichment at DMRs. ChIP-seq of H3K9me3 in replicates from WT, TKO_L,_ and DKO_0_ cells confirms the enrichment of the H3K9me3 modification over DMRs. **e**, Overlap of H3K9me3 peaks with DMRs. The majority of DMRs (88% and 90%) overlap with H3K9me3 peaks detected in TKO_L_ and DKO_0_ cells. *n* = 1,515 DMRs; *n* = 104,618 and 92,462 H3K9me3 peaks in TKO_L_ and DKO_0_, respectively. Of these DMRs overlapping H3K9me3 peaks, 50% also overlap with IAPEz-ints. **f**, Distribution of CpG methylation genome-wide and at H3K9me3 peaks. In the DKO_0_ P15, methylation levels are twice as high at H3K9me3 peaks (mean methylation rate 0.094) than at genomic regions not enriched for H3K9me3 (mean methylation rate 0.048). *n* = 1,127,488 and 17,772,311 CpGs, respectively. For boxplots within violin plots, white dot indicates the median, the boxes indicate first and third quartiles; whiskers, 1.5 x inter-quartile range; data beyond the end of the whiskers are omitted.

Our complementary and systematic analyses have established the in vitro and in vivo de novo methylation potential of Dnmt1, including specific targeting of DMRs enriched for IAP retrotransposons. We next sought to investigate the underlying mechanism and potential role of known Dnmt1 co-factors. Starting with Uhrf1, which is required for Dnmt1 maintenance methylation^9^, we utilized single guide (sg) RNAs with Cas9 to disrupt *Uhrf1* in the TKO_L_ background. Subsequently, Dnmt1 knockdown was reversed to derive Uhrf1 KO DKO_0_, followed by WGBS of two different clones at P7 and P15 (Fig. 4a and Extended Data Fig. 4a,b). Interestingly, none of the Uhrf1 KO DKO_0_ clones showed any detectable global or targeted methylation gain, suggesting that Uhrf1 is essential for Dnmt1’s maintenance and its de novo activity (Fig. 4b,c).

### H3K9me3, Trim28 and Uhrf1 overlap Dnmt1 de novo hotspots

Uhrf1 contains both a tandem Tudor domain and plant homeobox domain that act in concert to bind H3K9me3. Since H3K9me3 is known to be enriched at LTRs and to contribute to their repression in the pluripotent state, we hypothesized that it may play a role in the recruitment of Uhrf1 and Dnmt1 to DMRs^24–26^. As expected, we found our DMRs enriched for H3K9me3 in publicly available ESC chromatin immunoprecipitation sequencing (ChIP-seq) data (Extended Data Fig. 4c). We further validated the enrichment of H3K9me3 by performing ChIP-seq in the TKO_L_ and DKO_0_ cells and found that 88% and 90% of the DMRs overlap with H3K9me3 peaks, respectively (Extended Data Fig. 4d,e). Moreover, H3K9me3 peaks tend to be more methylated than other regions, suggesting that H3K9me3 may influence Dnmt1’s recruitment or activity at DMR regions (Extended Data Fig. 4f).

**Fig. 5:**
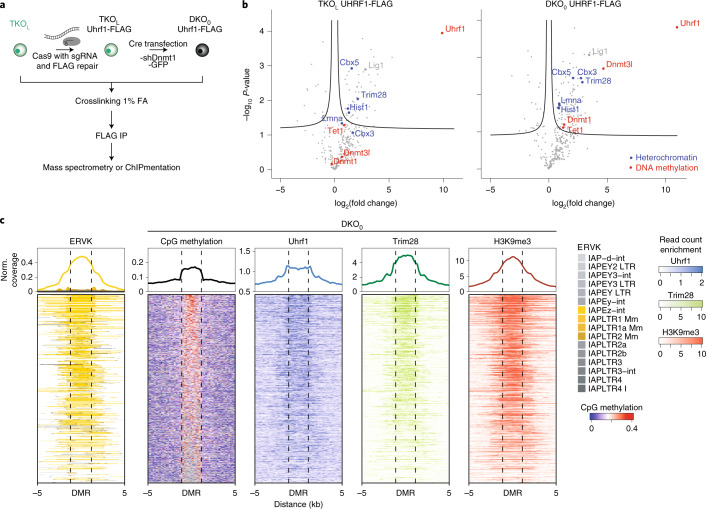
Dnmt1 de novo targets are enriched for Uhrf1, H3K9me3 and Trim28. **a**, Simplified schematic of the experimental design for generating Uhrf1-FLAG-tagged TKO_L_ and DKO_0_ cell lines and their analysis by MS and ChIPmentation. **b**, Volcano plot of interacting proteins acquired by rapid immunoprecipitation MS in both DKO_0_ and TKO_L_ cells using Uhrf1-FLAG as bait. Known Uhrf1 interactors such as Lig1, heterochromatin- and DNA methylation–associated proteins are indicated. Detailed results are listed in Supplementary Table 3. **c**, Integrative visualization of DMR loci and the chromatin environment. The signal is displayed as noise-subtracted read enrichment for pooled replicates. *n* = 2 ChIP-seq samples per condition.

**Extended Data Fig. 5:**
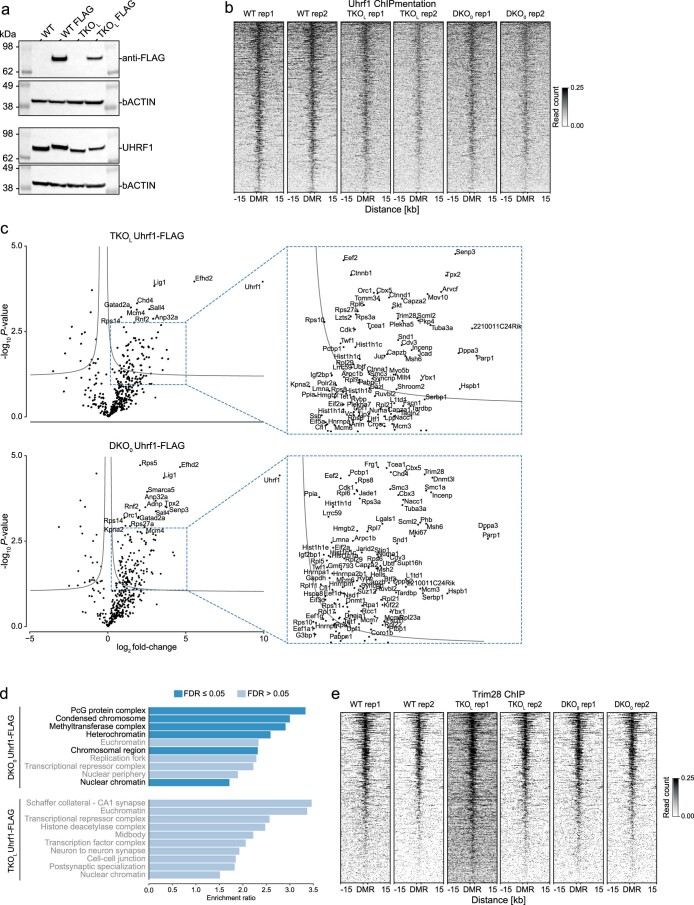
DMRs show enrichment of UHRF1 and TRIM28. **a**, Western blot confirming the FLAG tagged version of the endogenous Uhrf1. FLAG tag was recognized with an anti-FLAG antibody, with signal only observed in targeted cell lines. The UHRF1 blot shows the expected increase in the size of the UHRF1-FLAG protein and demonstrates comparable expression to non-FLAG tagged controls. *n* = 1. **b**, Heatmap of UHRF1 enrichment at DMRs. ChIPmentation-seq of FLAG-tagged Uhrf1 shows enrichment at DMRs for all replicates from WT, TKO_L,_ and DKO_0_ ESCs. The signal is displayed as fragment pile up per million reads per replicate. **c**, Volcano plot of interacting proteins acquired by Rapid Immunoprecipitation Mass Spectrometry (endogenous protein purification mass spectrometry) for TKO_L_ and DKO_0_ cells with endogenous Uhrf1-FLAG used as bait. Uhrf1-FLAG immunoprecipitation shows enrichment for known interactors like Lig1, heterochromatin, and DNA methylation associated proteins in both cell lines. Zoom highlights enriched proteins (see Supplementary Table 3). **d**, Visualization of a gene set enrichment analysis for TKO_L_ and DKO_0_ for Uhrf1 interacting proteins as detected by Rapid Immunoprecipitation Mass Spectrometry. Significant enrichment for heterochromatin and condensed chromatin as well as for methyltransferase complexes was found for the DKO_0_ cell line. **e**, Heatmap of Trim28 enrichment at DMRs. ChIP-seq of Trim28 shows enrichment over DMRs for all replicates from WT, TKO_L,_ and DKO_0_ ESCs. The signal is displayed as fragment pile up per million reads per replicate. Source data

Next, we integrated a FLAG tag at the endogenous *Uhrf1* locus and performed ChIPmentation (Fig. 5a, Extended Data Fig. 5a and Supplementary Table 1). Uhrf1 enrichment was observed over DMRs in DKO_0_, TKO_L_ and WT cells (Extended Data Fig. 5b). Of note, Dnmt1 has been reported to recognize Uhrf1, H3K18/23 ubiquitin (which is catalyzed by Uhrf1) and potentially dual-modified H3K9me3 and H3K18/23 ubiquitin. Therefore, one may speculate that the presence of H3K9me3 and Uhrf1 at DMRs could increase Dnmt1 activity or recruitment^11,27,28^.

To uncover other potential interaction partners involved in Uhrf1-dependent Dnmt1 de novo activity, we performed rapid immunoprecipitation mass spectrometry (MS) (Fig. 5a and Supplementary Table 3)^29^. Uhrf1 represented the most enriched protein relative to a non-FLAG-tagged DKO_0_ control, and known interacting partners like Lig1 were highly enriched, indicating a successful immunoprecipitation (Fig. 5b and Extended Data Fig. 5c)^30^. Gene ontology analysis of enriched proteins highlights terms associating with heterochromatin and the replication fork, another site of Uhrf1 recruitment (Extended Data Fig. 5d)^31^. As expected, Dnmt1 is enriched following Uhrf1 pulldown, confirming that these two proteins interact in the DKO_0_ cells. Furthermore, we also observed enrichment of Trim28, a heterochromatin scaffolding protein that is recruited to retrotransposons through zinc finger proteins, which is mechanistically interesting as it could potentially explain the enhanced Dnmt1 de novo activity at retrotransposons^32^. Interestingly, even in the absence of Dnmts or DNA methylation in TKO_L_ cells, Uhrf1 and TRIM28 enrich at DMRs and co-localize with proteins involved in repressive chromatin conformation like Cbx3, Cbx5 and the Hist1 family (Extended Data Fig. 5e).

Although still speculative based on our current data, the enrichment of Trim28 and deposition of H3K9me3 at the DMRs may provide an interaction scaffold for Uhrf1, which in turn could serve to recruit or activate Dnmt1 at these regions to facilitate its targeted de novo activity at IAPEz-ints (Fig. 5c).

### Dnmt1 de novo methylation correlates with repression

**Fig. 6:**
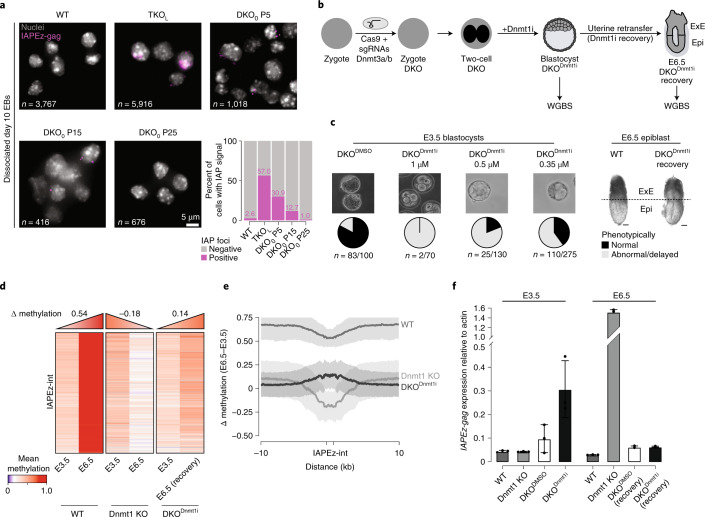
Dnmt1 de novo methylation and its effects in the early embryo. **a**, Representative images of IAPEz-gag expression assayed by RNA-FISH in dissociated EBs after 10 days of differentiation from WT, TKO_L_ and DKO_0_ cells. *n* = total counted nuclei. IAPEz-gag-positive cells out of total counted nuclei: WT 99/3,767; TKO_L_ 3,374/5,916; DKO_0_ P5 315/1,018; DKO_0_ P15 53/416; DKO_0_ P25 7/676. **b**, Experimental design for the generation of zygotic Dnmt3a/b DKO embryos and subsequent Dnmt1i (GSK3484862) treatment (from the two-cell stage until day E3.5 blastocyst) and retransfer for in vivo post-inhibitor recovery until E6.5. WGBS sample collections are indicated and include E3.5 (*n* = 8 WT, 12 Dnmt1 KO, 20 DKO, 8 DKO^DMSO^, 8 DKO^Dnmt1i^ embryos) and E6.5 (*n* = 3 epiblast samples). Epi, epiblast; ExE, extraembryonic ectoderm. **c**, Morphological and viability assessment of DKO^Dnmt1i^ E3.5 blastocysts. Left: representative images of DKO blastocysts treated with DMSO or different concentrations of Dnmt1i from the two-cell stage until day E3.5. Pie charts show the proportions of normal and delayed/abnormal blastocysts. *n* = number of normal blastocysts/total number of DKO 2-cell embryos included in each treatment. Right: representative images of WT and Dnmt1i-treated E6.5 embryos. Scale bars, 100 µm. **d**, Heatmap representation of mean methylation over IAPEz-int (*n* = 5,362) in the respective E3.5 blastocyst samples compared with E6.5 epiblasts. **e**, Profile plot of methylation changes between an E3.5 blastocyst and E6.5 epiblast. Shaded area represents standard deviation. **f**, RT-PCR quantification of *IAPEz-gag* expression relative to β-actin in WT, Dnmt1 KO, DKO^DMSO^ and DKO^Dnmt1i^ in E3.5 and retransferred E6.5 embryos. *n* = 3 replicates, with a single replicate consisting of a pool of 10 blastocysts from multiple mice or one E6.5 epiblast. Bars, mean; error bars, standard deviation. Quantification for **f** is provided in Supplementary Table 4. Additional source data can be found at 10.6084/m9.figshare.14555250.

**Extended Data Fig. 6:**
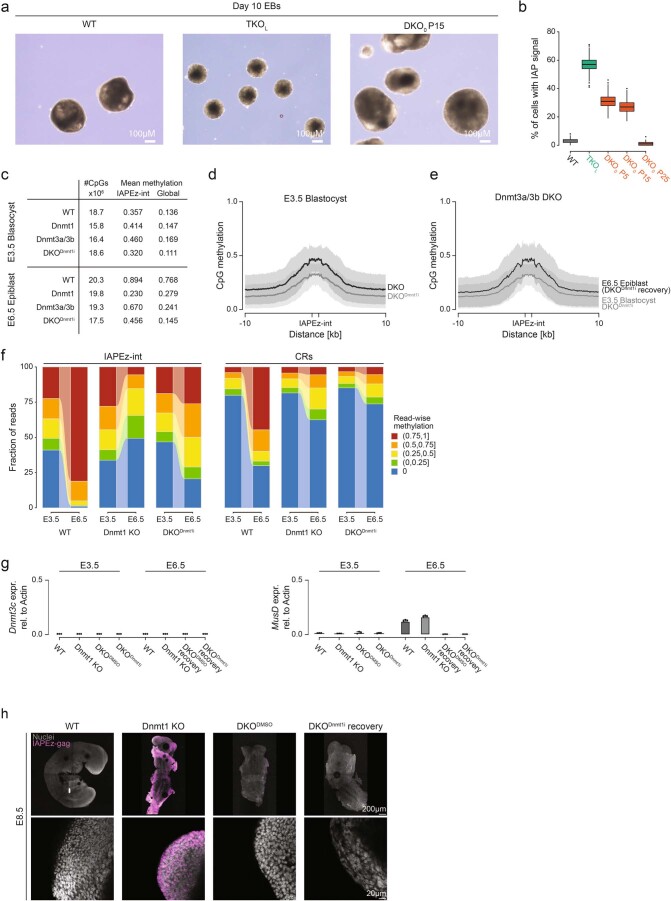
IAPEz-int methylation and expression are correlated. **a**, Representative images embryoid bodies (EBs) after 10 days of differentiation from WT, TKO_L_, and DKO_0_ P15. *n* = 100 EBs per condition. **b**, Distribution of number of cells with IAP signal determined *in silico* by randomly selecting 200 cells. The boxplots show the distribution of this sampling process over 1,000 repetitions; centerline is median; boxes, first and third quartiles; whiskers, 1.5 x inter-quartile range; data beyond the end of the whiskers are displayed as points. **c**, WGBS coverage and mean methylation levels for IAPEz-int and globally in E3.5 Blastocyst and E6.5 Epiblast. **d**, Profile plot depicting the methylation distribution over IAPEz-int elements E3.5 Dnmt3a/3b DKO (DKO) and Dnmt3a/3b DKO with Dnmt1 inhibitor-treated (DKO^Dnmt1i^) blastocysts. **e**, Profile plot depicting the methylation distribution over IAPEz-int elements for E3.5 DKO^Dnmt1i^ Blastocyst and the matching Dnmt1i recovery E6.5 Epiblast. **f**, Fraction of single read methylation levels in IAPEz-int and CRs for WT, Dnmt1 KO, and DKO^Dnmt1i^ at E3.5 to E6.5. **g**, RT-qPCR of *Dnmt3c* expression and a control retrotransposon *ETn-MusD* relative to *Beta-Actin* expression in WT, Dnmt1 KO, DKO^DMSO^, and DKO^Dnmt1i^ (0.35uM Dnmt1i from 2-cell until E3.5, retransfer at E3.5). *n* = 3 with ten Blastocysts or one E6.5 Epiblast per replicate. Bars denote the mean and the error bars denote standard deviation. **h**, IAPEz-gag expression assayed by RNA-FISH in WT, Dnmt1 KO, DKO^DMSO^, and DKO^Dnmt1i^ (treated with 0.35 uM Dnmt1i) embryos at E8.5. Dnmt1i and DMSO treatment as well as KO were carried out in the same manner as diagrammed in the zygotic KO pipeline illustrated in Fig. 6b with the omission of the WGBS step at E3.5/E6.5 for continued gestation. Bars denote the mean and the error bars denote mean plus standard deviation.

Next, we wanted to investigate whether the observed Dnmt1 de novo activity is sufficient to repress IAPEz-ints, which would indicate functional relevance. Previous work has shown that IAPs remain largely repressed in methylation-deficient pluripotent cells but not somatic cells^33–35^. Thus, we derived day 10 embryoid bodies (EBs) from WT, TKO_L_ and P5, P15 and P25 DKO_0_ cells and performed RNA fluorescence in situ hybridization (RNA-FISH) to visualize IAP expression using probes targeting the IAPEz-int specific gag sequence (Extended Data Fig. 6a and Supplementary Table 1). As expected, WT EBs do not show IAP expression, while the lack of methylation in the TKO_L_ EBs resulted in a high proportion of cells that showed de-repression of IAPEz-ints (Fig. 6a). Interestingly, the reversal of the knockdown and the subsequent Dnmt1-induced de novo gain of methylation in the ESCs, specifically at IAPEz-ints, is linked to a notable reduction in IAP expression in the derived EBs (Fig. 6a and Extended Data Fig. 6b).

### Dnmt1 de novo methylation of IAPEz-ints occurs in vivo

Finally, to return to a refined assessment of the Dnmt1 de novo activity in vivo, we generated zygotic knockouts of Dnmt1 and Dnmt3a/3b. We then treated the Dnmt3a/3b DKO with a non-covalent Dnmt1-specific inhibitor (GSK3484862) until the blastocyst stage (DKO^Dnmt1i^) and collected retransferred embryos for all conditions at E6.5. The inhibitor treatment was designed to transiently block Dnmt1 and reduce global DNA-methylation levels as much as possible without compromising viability, to facilitate the measurement of Dnmt1 activity globally and at IAPEz-ints (Fig. 6b,c). Treatment at a concentration of 0.35 μM still yielded 40% phenotypically normal blastocysts at E3.5. These were able to develop into morphologically normal E6.5 embryos, while higher concentrations resulted in defects at E3.5, such as reduced growth and impaired survival or failure to produce any viable E6.5 embryos (Fig. 6c). We then performed WGBS on WT, DMSO-treated control and DKO^Dnmt1i^ E3.5 blastocysts as well as DKO^Dnmt1i^ E6.5 epiblasts to evaluate the ability and extent of Dnmt1 de novo activity in this context. Comparing IAPEz-int and global methylation at E3.5 confirms the effect of Dnmt1 inhibitor treatment (Extended Data Fig. 6c). Although the lowly methylated global landscape at E3.5 showed comparable methylation levels in Dnmt3a/3b DKO and inhibitor-treated embryos, we observed a more substantial loss of methylation at IAPEz-ints in the latter. Intriguingly, a comparison of the DKO^Dnmt1i^ E3.5 to E6.5 recovery landscape showed a notable gain of methylation at IAPEz-ints (0.320 to 0.456 average methylation; Fig. 6d,e and Extended Data Fig. 6d,e). Read-level methylation analysis of Dnmt1 KO comparing E3.5 to E6.5 shows a gain of completely unmethylated reads in the Dnmt1 KO, in line with its perceived maintenance role. By contrast, the WT and DKO^Dnmt1i^ recovery both show an increase in read-level methylation with the number of reads containing some methylation in the recovery reaching a large proportion of WT levels (Extended Data Fig. 6f).

To determine the effect of the Dnmt1-induced focal gain of methylation at IAPEz-int, we analyzed IAPEz-gag expression in the different conditions (Fig. 6f, Extended Data Fig. 6g and Supplementary Table 4). In the E3.5 embryos, no considerable expression of IAPEz-gag could be detected in the WT or Dnmt1 KO embryos. We observed minimal de-repression in the Dnmt3a/3b DKO DMSO and much higher expression in the DKO^Dnmt1i^ E3.5 embryos, matching the more pronounced methylation difference (Fig. 6d–f). When comparing the E6.5 epiblasts, we could validate the increased expression of IAPEz-gag in the Dnmt1 KO, as previously shown^3,4^, and the persistent repression in WT embryos. Most strikingly, in concordance with the gain of methylation in the E6.5 DKO^Dnmt1i^ compared to E3.5 DKO^Dnmt1i^, we observed a reduction in *IAPEz-gag* expression, which is maintained at E8.5 (Fig. 6d–f and Extended Data Fig. 6h). This further supports the notion that Dnmt1 catalyzes de novo methylation during embryonic development and specifically at IAPEz-ints.

## Discussion

Dnmt1 is historically defined as the maintenance methyltransferase, and that description seems largely appropriate given its strong preference for a hemimethylated substrate. Further supporting this classification is the substantial loss of global methylation in *Dnmt1* knockouts and the highly discordant pattern of the remaining methylation. The latter suggests that, in the absence of Dnmt1, methylation is continuously added de novo by Dnmt3s, but then generally not maintained. As stated above, it has been noted that Dnmt1 has a much lower but potentially present de novo activity. However, to what degree and the role it might play in development had not been addressed so far. Our study adds two critical insights, along with several mechanistic details, to these questions. First, Dnmt1 in cells and the developing embryo can globally add methyl groups through a non-canonical de novo function. Second, this activity is specifically targeted to a sub-family of LTR retrotransposons and enables substantially higher methylation than background levels. Mechanistically, we establish that Dnmt1 is also dependent on its co-factor Uhrf1 for de novo activity and shows an association with H3K9me3- and TRIM28-enriched genomic regions. Although further study is needed, zinc finger proteins are known to recruit TRIM28 to ERV retrotransposons, which could serve to enrich both Uhrf1 and H3K9 tri-methyltransferases like Setdb1 at these regions^36^. Uhrf1 binding to Trim28 or H3K9me3 at the IAPs could then potentially increase the retention time of Dnmt1 at these locations, allowing for increased de novo and maintenance activity. Moreover, it provides a pathway for Uhrf1 recruitment independent of hemimethylated DNA. In the post-replication maintenance scenario, Uhrf1 would normally recognize the methylated strand and Dnmt1 the unmethylated to transfer the methyl group^9^.

How did this evolve and is it functionally relevant? The evolutionary origins of this activity will require further investigation. Still, we can speculate that, during specific periods such as in early primordial germ cells (PGCs), where Dnmt3s are downregulated, a combined de novo and maintenance function would appear to be of utility. Moreover, after implantation, the repressive mechanism for retrotransposon silencing becomes DNA-methylation-dependent, creating additional urgency to stably maintain high DNA-methylation levels at these sites.

**Extended Data Fig. 7:**
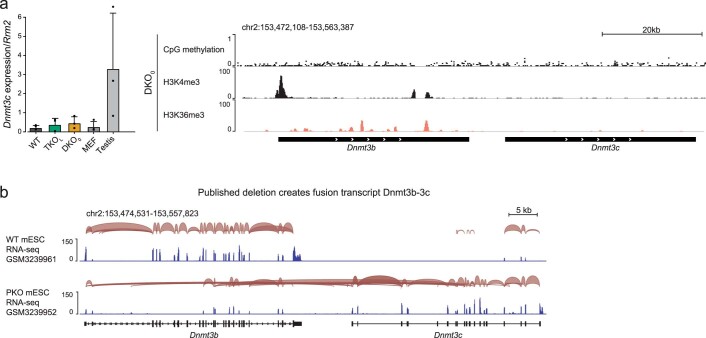
Catalytically active methyltransferase Dnmt3c is not expressed in DKO_0_ ESCs. **a**, RT-qPCR of Dnmt3c normalized to *Rrm2*. Lack of expression of Dnmt3c in WT ESC, TKO_L,_ and DKO_0_. Levels of expression present in ESC were similar to the mouse embryonic fibroblast (MEF, negative control); mouse testes serve as a positive control (*n* = 3, testes collected from three individual mice). Bars denote the mean and the error bars denote mean plus standard deviation. WGBS and ChIP-seq (top: WGBS, middle: H3K4me3, bottom; H3K36me3) at the *Dnmt3b* and *Dnmt3c* locus in the DKO_0_ state. Lack of H3K4me3 at the promoter and H3K36me3 across the gene body of *Dnmt3c* suggests no detectable transcription (WGBS *n* = 1, H3K4me3 *n* = 2, H3K36me3 *n* = 1). **b**, Re-analysis of splice sites of the RNA-seq from Wang *et al*.^19^, showing deletion associated read-through of the *Dnmt3b* promoter into the *Dnmt3c* gene, resulting in high read count levels for catalytic *Dnmt3c* isoforms.

One of our study’s main goals was to demonstrate that the observed de novo activity is indeed the result of Dnmt1, which we have done using a range of genetic tools. There are three additional known Dnmts—Dnmt2, Dnmt3c and Dnmt3l—that are worth briefly discussing^37^. Dnmt2 has been reported to catalyze transfer RNA methylation, and no discernible DNA-methylation activity was present in the TKO_L_ or TKO cells, excluding it as a potential factor in the DKO_0_ gain^38^. Dnmt3c is not normally expressed in ESCs and, even if it were present at low levels, its activity has no detectable effect based on our TKO_L_ or TKO cells (Extended Data Fig. 7a). A recently published study reported Dnmt3c activity upon a Dnmt3b deletion, which we confirmed creates a new fusion transcript and is therefore not generally present in ESCs but rather specific to that study (Extended Data Fig. 7b)^19^. Finally, Dnmt3l has no catalytic activity and is present in all ESC lines, again with no measurable impact. As no additional DNA methyltransferases are known at present, it leaves Dnmt1 as the sole possible catalytic enzyme responsible for our measured de novo gain in DKO_0_ or even more so in TKO cells rescued with ectopic, catalytically active Dnmt1.

In summary, we show that Dnmt1 has both de novo and maintenance activity directed towards IAP retrotransposons and may thereby contribute to their stable silencing in early development and possibly other contexts. Our insights highlight that even the well-established DNA-methylation field continues to evolve, and some classifications may need to be revisited as tools and knowledge expand.

## Methods

### Cell culture

J1 and KH2 mouse ESCs were acquired at the time of creation^22,39^ and V6.5 mouse ESCs were obtained from Konrad Hochedlinger. All lines tested negative for mycoplasma and were cultured in knockout DMEM medium (Gibco) containing 15% FBS, 1% penicillin/streptomycin, 1% glutamine, 1% non-essential amino acids (NEAA) and 10^5^ U leukemia inhibitory factor (LIF)^39^. For ESC maintenance, dishes were coated with 0.2% gelatin, and mitomycin-C-treated CD1 mouse embryonic fibroblasts (MEFs) were plated as a confluent layer of feeder cells. ESCs were seeded at a density of 50,000 cells per well of a six-well plate and were split every three days. All cell lines are available upon reasonable request.

### DKO_0_ cell line generation

DKO_0_ ESCs were generated by transiently transfecting clonal TKO_L_ ESCs^21,22^ with Cre recombinase (Addgene 24593) using the Amaxa 4D nucleofector X-Unit (Lonza) to remove the shRNA-GFP (GFP, green fluorescent protein) construct, followed by sorting for GFP-negative cells.

### TKO cell line generation

To generate Dnmt TKO ESCs, WT KH2 cells were initially transfected with px458 containing sgRNAs targeting the highly conserved PC motif in the catalytic domains of Dnmt3a and Dnmt3b. The resulting KH2 DKO cells were then transfected with px458 containing Dnmt1-specific sgRNAs to create TKO cells. The cells were transfected using the Amaxa 4D nucleofector X-Unit (Lonza) according to the manufacturer’s guidelines. Knockouts were verified by genotyping, western blot and quantitative PCR (qPCR).

### Uhrf1 FLAG line generation

To generate endogenous Uhrf1-3xFLAG cells, a gRNA ‘Uhrf1_FLAG_sg’ was cloned into pU6-(BbsI) CBh-Cas9-T2A-mCherry (Supplementary Table 1). WT V6.5 and TKO_L_ J1 ESCs were transfected with 10 μg of pU6-(BbsI) CBh-Cas9-T2A-mCherry-sgUhrf1, 10 µg donor plasmid coding for C-terminal homology arms and a 3xFLAG-Tag with a GS-linker (Ct_UHRF1_GGGGS2_3xFlag) using FuGENE and fluorescence-activated cell sorted (FACS) for mCherry-positive cells 48 h post-transfection. After several days of culture, individual colonies were picked, expanded and screened through PCR. The expression level of confirmed homozygous tagged Uhrf1 in isolated clones was assessed by western blot.

### Uhrf1 KO line generation

TKO_L_ ESCs were transfected with px330-mCherry containing sgRNAs targeting exon 1 or exons 3 and 6 of Uhrf1 (Supplementary Table 1). mCherry-positive cells were selected via FACS after 48 h. After replating and expansion, several clones were picked and propagated individually. These were screened by PCR and western blot.

### Dnmt1 piggybac plasmid construction

The coding sequence for V5-Dnmt1 was PCR-amplified from a synthesized codon-optimized Dnmt1 construct and introduced into pENTR4-V5 by Gibson assembly (Supplementary Table 1). Plasmids were isolated from transformed bacteria (One Shot ccdB Survival 2 T1R, Invitrogen) and the correct sequence of the constructs was confirmed by Sanger sequencing. The V5-Dnmt1 sequence was then cloned into the Piggybac expression vector pPB mCherry-3XFLAG-attR v2 (a modified version of plasmid SPB-007 (Transposagen) containing an mCherry-3XFLAG sequence followed by a ccdB and chloramphenicol resistance cassette flanked by attR sites) using LR clonase II (Clonase Gateway LR Clonase II enzyme mix, Invitrogen) according to the manufacturer’s recommendations. Plasmids were isolated from transformed bacteria (DH5α; Meissner Lab) and the correct sequence of the constructs was confirmed by Sanger sequencing.

### Dnmt1 rescue experiment

KH2 TKO cells were transfected with Dnmt1 piggyBac using the FuGENE HD transfection reagent (Promega) according to the manufacturer’s instructions. The KH2 TKO cells had been cultivated for 21 passages after the Dnmt1 KO. Cells were transfected in culture medium without antibiotics. After overnight incubation, the medium was replaced with standard culture medium. Successful transfection was assessed by FACS two days post-transfection and positive cells were placed back into culture for expansion. The integration of the construct was evaluated by FACS and numbers of integrations were quantified by qPCR. Positive clones were expanded and expression levels of the construct were confirmed by western blot (ab87654, Abcam) and RT–qPCR.

### Bisulfite amplicon sequencing

gDNA (500 ng) was subjected to bisulfite conversion using the EZ DNA Methylation-Gold kit according to the manufacturer’s instructions (Zymo). This was separated into four PCR reactions amplified for 15 cycles with IAPEz bisulfite primers using TaKaRa EpiTaq HS (Takara; Supplementary Table 1). The four reactions were purified and pooled using minElute columns (Qiagen) then subjected to end repair and A-tailing (NEB ultra II end prep), after which Illumina adapters were ligated (NEB adapter, NEB ultra II ligation module). The resulting adapter ligated fragments were purified using 0.85 volumes of Ampure beads (Beckmann Coulter). The resulting eluate was used in a PCR reaction with indexed Illumina PCR primers (NEBNext Multiplex Oligos for Illumina Index Primers Set 1). A double-sided Ampure bead purification was performed (0.65 then 0.85) and finished libraries were pooled then sequenced in paired-end 150 mode on a NovaSeq 6000 system.

### Oxford Nanopore library construction

Genomic DNA was isolated from 16 million cells using the GeneJET Genomic DNA extraction kit (Thermo). Two reactions of 7 μg gDNA were then end-repaired and a-tailed using Ultra II End-prep enzyme mix (NEB) according to the manufacturer’s recommendations. Adapter mix AMX1D (Oxford Nanopore) and Quick ligation mix (NEB) were added and the reaction was incubated at room temperature for 30 min. Adapter-ligated gDNA was then purified using Ampure XP magnetic beads (Beckman Coulter) at a bead-to-sample ratio of 0.5:1. Before elution, the beads were washed twice with ABB buffer (Oxford Nanopore) then incubated with Nanopore elution buffer (Oxford Nanopore) for 15 min at 37 °C. The finished library was added to running buffer (RBF) and library loading beads (LLB) (Oxford Nanopore) then loaded onto a flow cell.

### Hairpin bisulfite sequencing of repetitive elements

Hairpin bisulfite sequencing was performed according to ref. ^16^. Briefly, 200 ng of genomic DNA was digested with 10 U of BsaWI (NEB), DdeI (NEB), Eco47I (Thermo Fisher Scientific) or MspI (Thermo Fisher Scientific), incubated for 16 h at 60 °C (BsaWI) or 37 °C (DdeI, Eco47I and MspI) and afterwards heat-inactivated. Endonuclease-specific hairpin-linker (50 pmol) was ligated in a 20-µl reaction at 16 °C for 3 h. Bisulfite conversion was carried out using Zymo’s EZ DNA Methylation-Gold kit according to the manufacturer’s instructions. Bisulfite-treated hairpin-DNA was eluted in 20 µl of elution buffer. PCR was performed in 30-µl reactions with 2 µl of hairpin-bisulfite template and 37 PCR cycles using HotFirePol DNA polymerase (Solis BioDyne) or HotStarTaq DNA polymerase (Qiagen). PCR products were purified from a 1.2% agarose gel using Avegene’s GenepHlow Gel/PCR kit according to the manufacturer’s instructions and eluted in 20 µl 0.1× TE buffer. Amplicons were indexed in a 50-µl PCR using specific TruSeq indices and six PCR cycles. Index PCRs were purified with 1.1× AMPure XP beads (Beckman Coulter). Amplicons were sequenced on an Illumina MiSeq in a 2 × 250-bp sequencing mode.

### MeDIP library construction

The method was adapted from ref. ^40^. Briefly, DNA was extracted from 5 × 10^5^ to 1 × 10^6^ ESCs using phenol-chloroform-isoamyl alcohol (Thermo) extraction, then 500 ng of purified DNA was subjected to sonication on a Covaris S220 sonicator (10% duty cycle, intensity 5, 200 cycles per burst, six 1-min cycles). After sonication, fragmented DNA was end-repaired and A-tailed using an Ultra II End Repair/dA-Tailing module (NEB), then adapters (Broad Institute, single index P7) were ligated using Blunt/TA Ligase Master Mix (NEB). Both protocols were carried out as recommended by the manufacturer. A MagMeDIP kit (Diagenode) was used for precipitation and wash steps according to the manufacturer’s recommendations. Libraries were PCR-amplified with universal primers to add the P7/P5 graft sites using Phusion High-Fidelity PCR Master Mix with HF buffer (NEB), then size-selected between 200 and 700 bp on E-gel agarose gels 2% (Thermo). DNA was purified using a MinElute gel extraction kit (Qiagen). Purified DNA was quantified using Bioanalyzer HS DNA (Agilent) and qBit HS dsDNA (Thermo) kits then sequenced on a HiSeq 4000 system.

### Chromatin immunoprecipitation sequencing

Five (histone) or 25 (Trim28) million cells were crosslinked with 1% methanol-free formaldehyde (FA) (Thermo) for 5 or 8 min, respectively, at room temperature. Glycine was added to a final concentration of 125 mM and mixed for 5 min at room temperature. Crosslinked cells were washed with DPBS twice then spun down for 3 min at 15,000*g*. The cells were then incubated with 500 µl of cell lysis buffer (20 mM Tris-HCl pH 8.0, 85 mM KCl, 0.5% NP40) for 10 min on ice then spun down for 3 min at 2,500*g*. The supernatant was removed and the cell pellet was resuspended in 500 µl of nuclear lysis buffer (10 mM Tris-HCl, pH 7.5, 1% NP40, 0.5% sodium deoxycholate, 0.1% SDS) then incubated for 10 min on ice. The volume was increased to 1 ml using nuclei lysis buffer then sonicated on a Covaris E220 Evolution sonicator (peak incident power (PIP), 140.0; duty factor, 5.0; cycles per burst, 200; 10 min). After sonication, chromatin was spun down at 15,000*g* for 10 min to pellet insoluble material. The volume was increased to 1.5 ml with chip dilution buffer (0.01% SDS, 1.1% Triton X-100,1.2 mM EDTA, 16.7 mM Tris-HCl pH 8.1, 167 mM NaCl) and 2 µg of H3K4me3 antibody (Abcam, ab8580), H3K36me3 (Active Motif, 61101), H3K9me3 (Abcam, ab8898) or Trim28 (Abcam, ab22553) were added. The immunoprecipitation mixture was allowed to rotate overnight at 4 °C. Next day, 40 µl of Protein A/G Dynabeads (Thermo, 10001D) was added to the immunoprecipitation mixture and allowed to rotate for 4 h at 4 °C. This was followed by two washes of each of the following: low salt wash buffer (0.1% SDS, 1% Triton X-100, 2 mM EDTA, 20 mM Tris-HCl pH 8.1, 150 mM NaCl); high salt wash buffer (0.1% SDS, 1% Triton X-100, 2 mM EDTA, 20 mM Tris, pH 8.1, 500 mM NaCl); LiCl wash buffer (0.25 M LiCl, 1% NP40, 1% deoxycholate, 1 mM EDTA, 10 mM Tris-HCl pH 8.1) and TE buffer pH 8.0 (10 mM Tris-HCl, pH 8.0, 1 mM EDTA pH 8.0). DNA was eluted twice using 50 µl of elution buffer (0.5–1% SDS and 0.1 M NaHCO_3_) at 65 °C for 15 min. A 16-µl volume of reverse crosslinking salt mixture (250 mM Tris-HCl, pH 6.5, 62.5 mM EDTA pH 8.0, 1.25 M NaCl, 5 mg ml^−1^ Proteinase K) was added, and samples were allowed to incubate at 65 °C overnight. For library preparation, DNA was purified using AMPure XP beads (Beckman Coulter) and treated with DNase-free RNase (Roche) for 30 min at 37 °C. DNA libraries were then end-repaired and A-tailed using an Ultra II End Repair/dA-Tailing module (NEB) and adapters (Broad Institute, single index P7) were ligated using Blunt/TA Ligase Master Mix (NEB). Next, libraries were PCR-amplified using Pfu Ultra II Fusion High-fidelity DNA polymerase (Agilent) then size-selected on a gel for fragments between 200 and 1,000 bp.

### ChIPmentation

Cells were washed once with PBS and fixed with 1% methanol-free formaldehyde (Thermo) for 10 min at room temperature with rotation. The formaldehyde was quenched with 125 mM glycine for 5 min at room temperature. Cells were spun at 500*g* for 10 min at 4 °C and washed twice with ice-cold PBS supplemented with Protease Inhibitor cOmplete. Subsequent work was performed on ice and with buffers cooled to 4 °C. The pellet was lysed in L3B buffer (10 mM Tris-HCl, pH 8.0, 100 mM NaCl, 1 mM EDTA, 0.5 mM EGTA, 0.1% sodium deoxycholate, 0.5% *N*-lauroylsarcosine, 1× protease inhibitors) and sonicated in a 130 µl milliTUBE in a Covaris E220 for 7 min until most of the fragments were 200–700 base pairs long (settings: duty cycle, 5%; peak incident power, 140 W; cycles per burst, 200). Lysates were supplemented with 1% Triton X-100 and centrifuged at full speed for 5 min at 4 °C, and the supernatant containing the sonicated chromatin was transferred to a new tube. In parallel, Protein G magnetic beads (Invitrogen) were blocked and conjugated to an antibody by washing them twice in PBS with 0.5% BSA and resuspended in 200 μl of PBS with 0.5% BSA per immunoprecipitation. Anti-Flag (1 μg, Millipore/Sigma F1804) was added and bound to the beads by rotating for >1 h at room temperature. Blocked antibody-conjugated magnetic beads were added to the tube containing the chromatin and incubated overnight at 4 °C. The beads were then washed twice with each of the following: TFWBI (20 mM Tris-HCl/pH 7.4, 150 mM NaCl, 0.1% SDS, 1% Triton X-100, 2 mM EDTA), TF-WBIII (250 mM LiCl, 1% Triton X-100, 0.7% sodium deoxycholate, 10 mM Tris-HCl/pH 8, 1 mM EDTA) and 10 mM Tris-HCl pH 8. Beads were resuspended in 24 μl of tagmentation buffer and 1 μl of Tn5 transposase (Illumina 15027866, 15027865) and then incubated at 37 °C for 5 min in a thermocycler. Tagmentation reactions were removed and beads were washed twice with WBI and TET (0.2% Tween-20, 10 mM Tris-HCl/pH 8.0, 1 mM EDTA) (twice). Beads were then incubated with 70 μl of elution buffer (0.5% SDS, 300 mM NaCl, 5 mM EDTA, 10 mM Tris-HCl pH 8.0) containing 2 μl of Proteinase K (NEB) for 1 h at 55 °C and 8 h at 65 °C to reverse crosslink, and the supernatant was transferred to a new tube. Another 30 μl of elution buffer was added to the beads and incubated with another 1 μl of Proteinase K for 1 h at 55 °C, then the eluates were combined. Finally, DNA was purified with AMPure XP beads (sample-to-beads ratio of 1:2). Relative quantitation was performed using SYBR Green as in ref. ^41^ using 2 μl of DNA. Libraries were amplified according to the *C*_q_ values obtained in the previous step (12 cycles were used), purified using AMPure XP beads and eluted in 15 μl of water^41^.

### WGBS library construction

Genomic DNA (100–200 ng) was fragmented using a Covaris S2 system for 6 min according to the following program: duty cycle, 5%; intensity, 10; cycles per burst, 200. The sheared DNA was purified using the DNA Clean and Concentrator kit from Zymo. Bisulfite conversion of DNA was then conducted using the EZ DNA Methylation-Gold kit (Zymo Research), eluting in 15 µl low TE buffer. To minimize loss during storage, bisulfite-converted DNA was immediately processed for generating WGBS libraries using the Accel-NGS Methyl-Seq DNA library kit (Swift Biosciences). All protocols were carried out according to the manufacturer’s specifications. The libraries were sequenced as 150-bp paired-end reads on an Illumina NovaSeq system.

### Co-immunoprecipitation mass spectrometry

The protocol was implemented as published in ref. ^29^. Briefly, 50 million cells were crosslinked with 1% methanol-free formaldehyde (Thermo) for 8 min, followed by quenching with 125 mM glycine for 5 min. Crosslinked cells were lysed with 10 ml lysis buffer 1 (50 mM HEPES-KOH pH 7.5, 140 mM NaCl, 1 mM EDTA, 10% (vol/vol) glycerol, 0.5% (vol/vol) NP40/Igepal CA-630 and 0.25% (vol/vol) Triton X-100) for 10 min at 4 °C then centrifuged to pellet the cells (5 min 2,000*g* at 4 °C). The pellet was then dissolved in 10 ml lysis buffer 2 (10 mM Tris-HCl (pH 8.0), 200 mM NaCl, 1 mM EDTA and 0.5 mM EGTA) and incubated for 10 min at 4 °C followed by another centrifugation with the same parameters. Lysis buffer 3 (1.5 ml) (10 mM Tris-HCl (pH 8.0), 100 mM NaCl, 1 mM EDTA, 0.5 mM EGTA, 0.1% (wt/vol) sodium deoxycholate and 0.5% (vol/vol) *N*-lauroylsarcosine) was used to resuspend the pellet. The cell suspension was sonicated for 25 min on a Covaris E220 Evolution system (PIP, 140.0; duty factor, 5.0; cycles per burst, 200). Triton X (10% vol/vol) was added and the lysate was centrifuged for 10 min at 20,000*g*. Cleared lysate was removed and 10 μg of anti-Flag antibody (Supplementary Table 1) precoupled to protein G beads (Invitrogen) was added. The solution was allowed to incubate overnight and washed 10 times with RIPA buffer (50 mM HEPES (pH 7.6), 1 mM EDTA, 0.7% (wt/vol) sodium deoxycholate, 1% (vol/vol) NP40 and 0.5 M LiCl) the following day. This was followed by two washes with 100 mM ammonium bicarbonate. Proteomics sample preparation was performed according to a published protocol with minor modifications^42^. In brief, three biological replicates of DKO_0_ Uhrf1-FLAG, TKO_L_ Uhrf1-FLAG and untagged control samples were subjected to denaturing conditions and sequentially digested with LysC and trypsin (Roche). Peptide desalting was performed according to the manufacturer’s instructions (Pierce C18 Tips, Thermo Scientific). LC-MS/MS was carried out by nanoflow reverse-phase liquid chromatography (Dionex Ultimate 3000, Thermo Scientific) coupled online to a Q-Exactive HF Orbitrap mass spectrometer (Thermo Scientific), as reported previously^43^. Briefly, the LC separation was performed using a PicoFrit analytical column (75 μm (ID) × 50 cm (length), tip ID of 15 µm; New Objectives) in-house-packed with 3-µm C18 resin (Reprosil-AQ Pur, Dr. Maisch). Raw MS data were processed with MaxQuant software (v1.6.0.1) and searched against the mouse proteome database UniProtKB (UP000000589) with 22,286 entries, released in December 2018. The MaxQuant processed output files are provided in Supplementary Table 3, which shows peptide and protein identification, accession numbers, percent sequence coverage of the protein, *q*-values and label-free quantification (LFQ) intensities. The MS data have been deposited to the ProteomeXchange Consortium (http://proteomecentral.proteomexchange.org) via the PRIDE partner repository^44^ with the dataset identifier PXD025736. For interactor identification, *t*-test-based statistics were applied on LFQ. First, the logarithms (log_2_) of the LFQ values were taken, resulting in a Gaussian distribution of the data. This allowed the imputation of missing values by a normal distribution (width, 0.3; shift, 1.8), assuming these proteins were close to the detection limit. Statistical outliers for the pulldown of UHRF1-FLAG were compared to untagged UHRF1 and then determined using a two-tailed *t*-test. Multiple testing correction was applied by using a permutation-based false discovery rate method in Perseus.

### Western blotting

Cells were lysed in RIPA buffer and incubated at 4 °C for 30 min, then centrifuged for 15 min at 13,000*g*. Supernatant containing soluble proteins was transferred to a new tube to which NuPAGE LDS sample buffer 4× (Thermo) and reducing reagent 10× (Thermo) were added to 1× concentrations. The sample was then denatured for 10 min at 70 °C, then 10 µg of the sample, concentration determined by bicinchoninic acid protein assay (Thermo), was run on a NuPage 4–12% Bis-Tris protein gel in 1× MOPS buffer with 10 µl of SeeBlue Pre-Stained Protein Standard (Life Technologies) for 1.5 h at 100 V. Protein was then transferred to a PVDF membrane for 9 min at 25 V using the iBlot2 system (Invitrogen). The membrane was blocked in 5% non-fat milk, cut into two sections and incubated with primary antibody (FLAG Sigma F1804 1:1,000; Dnmt1 Abcam ab87654 1:1,000; Uhrf1 Santa-Cruz sc-373750 1:250; GAPDH Cell Signalling 14C10 1:1,000; Lamin-B Abcam ab8982 1:1,000; β-actin Abcam ab8226 1:1,000; Supplementary Table 1) overnight at 4 °C, washed three times for 10 min in PBS with 0.1% (vol/vol) Tween and incubated with species-specific horseradish peroxidase secondary antibody (1:10,000 Jackson Laboratory 115-035-174 or 211-032-171) for 1 h. Following three 10-min washing steps in PBS with 0.1% Tween, the protein bands were visualized using SuperSignal West Dura Extended Duration Substrate (Thermo).

### RNA fluorescence in situ hybridization for IAPEz-gag on EBs

Before hanging drop production, WT, TKO_L_ and DKO_0_ P15 ESCs were dissociated to single cells using TryplE (Thermo), and MEFs were depleted by replating the cells for 45 min on gelatin-coated culture plates. Floating cells were gently collected and passed through a 50-μm filter (Roth) counted and were diluted to a concentration of 20 cells μl^−1^ in differentiation medium (growing medium, 20% FBS without LIF). EBs were established in 25-μl hanging drops on low-adherent bacterial culture dish covers, allowing 500 cells per drop to aggregate for 48 h. After two days, 100 EBs were pooled into a 10-cm low-adherent bacterial culture dish and differentiation medium was replaced every other day. Cells were disassociated with accutase (Sigma) and then seeded on poly-l-lysine-coated glass coverslips and allowed to adhere for 10 min. Coverslips were washed twice with 1× PBS and fixed with 4% PFA for 10 min. After washing twice with 1× PBS, cells were permeabilized in 70% ethanol overnight at 4 °C. RNA-FISH was performed with the Stellaris buffers and protocol, with some modifications. Briefly, coverslips were incubated for 15 min in wash buffer A before hybridization with probes. IAPEz transcripts were detected using an oligo probe with 5′Cy3 label for the *gag* region of the IAPEz repeat sub-family^45^. The probe was resuspended in TE buffer to a stock concentration of 12.5 µM, and a working concentration of 125 nM in hybridization buffer was used. Hybridization was carried out in an equilibrated chamber at 37 °C for 6–8 h. Coverslips were washed twice in wash buffer A for 30 min at 37 °C. Nuclei were counterstained for 5 min with 0.2 µg ml^–1^ DAPI. Coverslips were washed in wash buffer B for 5 min at room temperature, followed by mounting on glass slides with ProLongGold antifade mounting medium.

Images were acquired using a ×100 oil immersion objective (NA = 1.4) on an Axio Observer Z1/7 running under ZEN 2.3 software. For each sample and replicate, 100–200 single tile regions were defined and the optimal focus was adjusted visually on the nuclear counter stain. The focused image was used as a center for a *z*-stack of 11 slices with a section thickness of 1.0 µm between individual slices. Thereby, a total stack height of 11 µm was collected covering slightly more than a single cell height to ensure that the cell patch would be captured in all three dimensions. Image acquisition was performed using a Zeiss Axiocam 506 system in a 5 × 5 binning mode, resulting in a lateral resolution of 0.22 µm pixel^−1^. The resulting images were projected using maximum intensity projection (MIP) in a ZEN 3.2 (Zeiss) dedicated analysis workstation. Object quantification was performed in the image analysis module in ZEN 3.2 (Zeiss). Briefly, within MIPs, primary objects/cells/nuclei were identified by nuclear counter staining using Otsu intensity thresholds after faint smoothing (Gauss 2,0), and nearby objects were segmented downstream by standard water shedding. Secondary objects were identified exclusively within primary objects by applying local rolling ball background subtraction in the primarily defined nuclei in the respective fluorescence image. Secondary objects were identified with a subsequent fixed intensity threshold. All objects were filtered according to circularity and area. From the resulting object, 250 individual cells per condition were randomly sampled using R to run statistics. All images are presented without background subtraction.

### Whole-mount RNA fluorescence in situ hybridization for IAPEz-gag on embryos

Whole-mount RNA-FISH was performed according to the recommended protocol from HCR Molecular Instruments with the modifications outlined below. WT and mutant embryos were dissected at E8.5 from uteri of surrogate mice and immediately fixed in 4% PFA overnight at 4 °C. Embryos were washed twice for 10 min in PBST (1× PBS + 0.5% Tween-20). Embryos were dehydrated in methanol with a series of graded methanol/PBST washes for 10 min each on ice (25% MeOH/75% PBST; 50% MeOH/50% PBST; 75% MeOH/25% PBST; 100% MeOH; 100% MeOH), followed by storage in 100% MeOH at −20 °C. Embryos were transferred to 2-ml tubes and rehydrated with a series of graded MeOH/PBST washes for 10 min each on ice (75% MeOH/25% PBST; 50% MeOH/50% PBST; 25% MeOH/75% PBST; 100% PBST; 100% PBST), followed by 100% PBST for 10 min at room temperature. Embryos were treated with 10 µg ml^−1^ Proteinase K for 8 min at room temperature, followed by two washes of 5 min each with PBST. Embryos were post-fixed with 4% PFA for 20 min at room temperature, followed by three washes of 5 min each with PBST. Embryos were pre-hybridized by incubating in probe hybridization buffer for 5 min at room temperature, followed by another incubation for 30 min at 37 °C. Probes were resuspended in 500 µl of probe hybridization buffer to a final concentration of 1 pmol and hybridized overnight at 37 °C. Embryos were washed six times for 15 min each in 1 ml of probe wash buffer at 37 °C, and four washes in 5X SSCT (5X SSC + 0.1% Tween-20) at room temperature. Hairpin probes (h1 and h2) were prepared separately by denaturing 10 µl of each (from 3 µM stock) for 90 s at 95 °C and snap-cooling in the dark for 30 min at room temperature. Embryos were incubated in amplification buffer for 10 min at room temperature, followed by incubation with both hairpins in 500 µl of amplification buffer at room temperature overnight in the dark. Embryos were washed in 2 ml of 5X SSCT at room temperature (twice for 5 min each; three times for 30 min each; twice for 5 min each). Embryos were incubated in 0.5 µg ml^−1^ DAPI solution for 1 h at room temperature and mounted in drops of 5X SSCT for imaging. Images were acquired on a Zeiss LSM-880 confocal microscope at ×10 and ×63 magnification with averaging four times per frame and 10-µm *z*-stacks. Images were processed in Fiji and maximum intensity projection is represented. All images are presented without background subtraction. HCR probes for IAPEz-B1 are available at Molecular Instruments lot no. PRF680.

### Dnmt1 inhibition and recovery in vivo

All mice were kept under specific-pathogen-free conditions in individually ventilated cages at a temperature of 22 ± 2 °C and a humidity of 55 ± 10% with a 12-h light/dark cycle (7:00 to 19:00). B6D2F1 females (7–9 weeks of age; Envigo) were superovulated with 5 IU of pregnant mare serum gonadotropin (PMSG) followed by 5 IU of human chorionic gonadotropin (HCG) after 46 h. MII-stage oocytes were isolated after 12 h and cultured in pre-gassed KSOM drops. Zygotes were generated by in vitro fertilization (IVF) with F1 (B6/CAST > 2 months of age) sperm, as previously described^46^. Pro-nuclei stage 3 zygotes were washed in M2 medium before electroporation. Assembly of the Alt-R CRISPR-Cas9 ribonucleoprotein and electroporation were performed as previously described^4^. Two-cell stage (2C) embryos were then incubated with Dnmt1i (GSK3484862) or with DMSO. Blastocysts were scored for morphology, size and viability and then retransferred to pseudo-pregnant CD1 female fosters. E6.5 embryos were recovered and used for expression or methylation analysis.

All procedures were performed in our specialized facility, and we followed all relevant animal welfare guidelines and regulations. Protocols were approved by Harvard University IACUC protocol (28-21) and the Max Planck Institute for Molecular Genetics (G0247/13-SGr1).

### WGBS of in vivo samples

E6.5 embryos were collected in M2 medium, Reichert’s membrane was completely removed before ExE/epiblast separation. ExE and epiblast were physically separated by cutting at the interface with a fine glass capillary. The visceral endoderm was removed from the ExE/epiblast by incubating in 1× non-enzymatic cell dissociation solution (Sigma-Aldrich) for 30 min at 4 °C, followed by slowly peeling off the layer by passing it through a glass capillary. Between 3 and 20 embryos were pooled and digested overnight at 55 °C in 200 μl of gDNA lysis buffer (10 mM Tris-HCl pH 8.0, 10 mM NaCl, 10 mM EDTA, 0.5% SDS plus fresh 300 μg ml^−1^ Proteinase K). gDNA was then extracted using 200 μl phenol:chloroform:isoamyl alcohol and precipitated with 2 μl of molecular-grade glycogen, 10 μl of 5 M NaCl and 500 μl 100% ethanol at −20 °C overnight. The gDNA precipitate was spun for 45 min at 4 °C at maximum speed and washed once with 1 ml of 70% ethanol. Following a second spin at 4 °C for 45 min, the gDNA pellet was air-dried for 10 min and resuspended in 50 μl of Swift low-EDTA TE buffer. The resulting gDNA was sonicated using a Covaris s220 system (duty cycle, 10%; intensity, 4; cycles per burst, 200; 35 s per cycle; three cycles total). The sonicated fragments were analyzed for the correct size using a Tape Station D5000 instrument. The fragmented gDNA was then bisulfite-converted using the Zymo EZ DNA Methylation-Gold kit following the manufacturer’s instructions. WGBS libraries were constructed from the bisulfite-converted gDNA using the Swift Accel-NGS Methyl-Seq DNA Library kit following the manufacturer’s instructions. Final libraries were amplified with 9–12 cycles.

### RNA isolation and quantitative real-time PCR

RNA from E3.5 and E6.5 embryos was isolated using a PicoPure RNA isolation kit following the manufacturer’s protocol. Complementary DNA (cDNA) was prepared with ~80% of the total RNA yield using a High-Capacity cDNA Reverse Transcription kit following the manufacturer’s protocol. For ESCs, an RNeasy kit (Qiagen) was used to extract RNA and a RevertAid First Strand cDNA Synthesis kit (Thermo) was used to generate cDNA (both used in accordance with the manufacturer’s recommendations).

### Computational methods

Computational methods are provided in Supplementary Note 1.

### Reporting Summary

Further information on experimental design is available in the Nature Research Reporting Summary linked to this Article.

## Online content

Any methods, additional references, Nature Research reporting summaries, source data, extended data, supplementary information, acknowledgements, peer review information; details of author contributions and competing interests; and statements of data and code availability are available at 10.1038/s41594-021-00603-8.

## Supplementary information

Supplementary Information Computational methods.

Reporting Summary

Peer Review Information

Supplementary Table 1 Overview of primers, sgRNAs, plasmids, antibodies and small molecules, and hairpin bisulfite linkers and consensus sequences, used in this study.

Supplementary Table 2 Location of DMRs between P15 DKO_0_ and TKO_L_.

Supplementary Table 3 Results from the mass spectrometry experiments.

Supplementary Table 4 Raw Ct values and quantification from quantitative PCR analysis.

## Data Availability

All sequencing data have been deposited in the Gene Expression Omnibus (GEO) under accession code GSE158460. Previously published in vivo mouse embryo WGBS data were used for in vivo comparisons^12,13^, and the data are available from GEO under accession codes GSE137337 and GSE84235. Encode histone modifications (H3K4me1, H3K4me3, H3K27me3, H3K9me3, H3K27ac, H3K36me3, H3K9ac) were downloaded from NCBI (GSE31039). MS data are available on PRIDE using the accession code PXD025736. Source data for figures are deposited online at 10.6084/m9.figshare.14555250. Source data are provided with this paper.

## Source data

Source Data Fig. 2 Unprocessed western blots. Source Data Fig. 4 Unprocessed western blots. Source Data Extended Data Fig. 1 Unprocessed western blots. Source Data Extended Data Fig. 5 Unprocessed western blots.

## Code availability

Code is available at https://github.com/HeleneKretzmer/Dnmt1_de_novo.
