# Supplementary material for: Dnmt1 has de novo activity targeted to transposable elements
Source: Nat Struct Mol Biol. 2021 Jun 17;28(7):594–603. doi: 10.1038/s41594-021-00603-8 (PMC8279952; doi:10.1038/s41594-021-00603-8)
Supplement: Supplementary file 1 — Computational methods. [file 41594_2021_603_MOESM1_ESM.pdf]

nature portfolio

https://doi.org/10.1038/s41594-021-00603-8

Supplementary information

Dnmt1 has de novo activity targeted to transposable elements

In the format provided by the authors and unedited

Computational Methods

Long-read Nanopore sequencing processing

All Oxford Nanopore Technologies derived data (PromethION) were processed using the Nanopype pipeline (v1.1.0)[1]. The base-caller Guppy (v4.0.11) was used with the r9.4.1 high-accuracy configuration. Quality filtering was disabled for any base-calling. Base-called reads were first aligned against the mouse reference genome mm9 using minimap2 (v2.10)[2] with the ont parameter preset. Structural variations were called using Sniffles (v1.0.10)[3]. Sniffles was configured to report variations of at least 500 bp, supported by at least five reads from read fragments longer than 2 kb (-l 500 –s 5 –r 2000).

Genome wide methylation rates were obtained by aligning all reads against reference genome mm9 using NGMLR (v0.2.7) and calling methylation using Nanopolish (v0.13.2, default parameters)[4]. NGMLR is only reporting primary and supplementary alignments, in the case of equal scores for multiple positions the read is reported to be unmapped. The output was filtered for individual CpGs with an absolute log-likelihood ratio greater 2.0 and genomic positions with 15x coverage.

Whole-genome bisulfite sequencing (WGBS) processing

WGBS sequencing data were checked and corrected for sequencing quality and adapter content using fastQC (v0.11.8) and cutadapt (v2.4). Subsequently, the reads were aligned to the mouse mm9 reference genome using BSmap (v2.9.0) with flags -v 0.1 -s 16 -w 100 -S 1 –q 20 –u -R[5]. The methylation level of all CpGs captured was calculated using the mcall module (v1.3.2) in the MOABS software suite with standard parameter settings[6]. Finally, all CpGs that were covered with less than 10 reads (10x) were discarded from the analysis.

Assessment of global, genome-wide methylation, as well as region-specific methylation, was performed using average (arithmetic mean) methylation levels. Replicates were grouped after filtering for coverage by calculating the arithmetic mean of the remaining positions given their presence in at least one replicate.

Amplicon bisulfite sequencing processing

WGBS sequencing data were aligned to the mouse full length IAPEz-int flanked by IAPLTR1 consensus sequences using BSmap (v2.9.0) with flags -v 0.1 -s 16 -w 100 -S 1 –q 20 –u -R[5]. The methylation level of all CpGs captured was calculated using the mcall module (v1.3.2) in

the MOABS software suite with standard parameter settings[6]. Finally, all CpGs that were covered with less than 1,000 reads (1,000X) were discarded from the analysis.

Hairpin bisulfite processing

CpG methylation states were determined using BiQAnalyzerHT, filtering for a sequence identity of >= 0.8 (reference sequence for IAP and IAPez have been shortened in order to obtain a better mapping of the sequencing reads) and strand-specific methylation patterns were obtained using Hairpinanalyzer V0.3 (Mathias Bader and Julia Arand, available under: https://github.com/PascalGiehr/Hairpinanalyzer), filtering for a linker conversion rate of >= 0.8. H(O)TA was applied in order to determine enzyme efficiencies[7,8].

MeDIP-seq processing

The MeDIP-seq sequencing data were aligned to the mouse reference genome mm9 using segemehl (v0.2.0, default parameters)[9-11] and the alignment was filtered to remove unusual insert sizes (>1kb) and multiple mappers. Postprocessing was done using the R package QSEA[12] with the DKO_0 methylation rates as reference.

ChIP-seq processing

The ChIP-seq sequencing data as well as the control input sequencing were aligned to the mouse mm9 reference genome using BWA mem[13] using the default parameter. GATK[14] was used to obtain alignment metrics and remove duplicates. Peaks were called using the MACS2 (2.1.2_dev)[15] peakcall function using default parameters. After validation of replicate comparability and quality, replicates were merged on read level and reprocessed together with input samples. Background subtracted coverage files were obtained using MACS2 bdgcomp with -m FE.

Bioinformatics tools, statistics, and visualization

Command-line processing of BAM, BED and bigwig files was done using SAMtools (v1.10)[16], BEDtools (v2.25.0)[17] and UCSCtools (v4)[18]. If not stated otherwise: All statistics and plots are generated using R version 3.6.1 “Action of the Toes”. In all boxplots, the centerline is median; boxes, first and third quartiles; whiskers, 1.5 x inter-quartile range; data beyond the end of the whiskers are displayed as points. Statistical tests for increased base pair overlap between DMRs and CRs with genomic elements were carried out using the Wilcoxon test (R function wilcox.test, alternative ‘greater’). Heatmaps were plotted using the R Complex Heatmap

(v1.99.5)[19] and Enriched Heatmap (1.19.2)[20] package. Further visualization was done using the smoothScatter function, the vioplot (v0.3.5), and ggplot2 (v3.3.2) packages. Profile plots and ChIP-seq/MeDIP-seq heatmaps were created with deepTools (v.2.5.4)[21], and browser track figures using IGV (v2.9.2)[22].

Differentially methylated regions

Differentially methylated regions (DMRs) were called using a dual sliding window approach. To capture smaller windows, the first window was defined as 1 kb in length and 250 bp sliding, for broader regions the second window was defined as 2 kb and 500 bp sliding. Windows covering less than 10 CpGs or located in regions called as missing based on Nanopore sequencing were excluded from the analysis. For each of the remaining windows, the average methylation rate difference between the P15 DKO_0 and TKO_L samples was calculated and any window with an absolute difference >0.15 was considered a potential DMR. Finally, all DMRs within 5 kb distance were merged and regions with an average methylation rate of >0.15 in the DKO_0 P15 were considered as DMRs.

Control regions

Control regions (CRs) were resampled from the genome using BEDtools shuffle. DMR length distribution (in nt) and the number of DMRs were preserved and only regions with 10 or more CpGs were allowed as a destination for BEDtools shuffle. To obtain these regions, we merged all CpGs covered by TKO_L and DKO_0 samples that are within a distance of 2.5 kb and filtered for a minimum of 10 CpGs. The identity of the original DMR was transferred to allow for the exclusion of the CR if the DMR was removed based on Nanopore sequencing data analysis. Each DMR was placed randomly 1,000 times to avoid strong impact of outlier samples. Additionally, the same number of CRs were placed only into regions with DMR-like CpG density to create the CpG-density matched CRs (CR^{CpG}).

Filtering for high confidence DMRs

DMRs were filtered based on Nanopore long reads from the same cell line by stringently filtering on methylation difference as measured from Nanopore read data. Only DMRs that had at least 10 informative CpG positions were subject to filtering and any DMR with a mean difference between the DKO_0 and TKO_L samples of less than 0.05 was excluded (n = 1,058). The corresponding control regions were removed from the set of CRs used for further analysis (n = 1,515).

Comparison of alignments at DMRs/CRs

For Illumina sequenced WGBS data global as well as alignments overlapping DMRs and CRs were counted individually using the SAMtools view command for counting (-c), DMRs, or CRs specified by -L and filtered out (-F) of specific bitwise flags. Unique alignments of Illumina short-read WGBS were defined by the not set "not primary alignment" SAM flag (bitwise flag 256). For Nanopore long-read alignments, the aligner does not report any reads that align to more than one location with the same quality. To validate that DMRs are not biased towards lower coverage with this approach, the read coverage distribution of DMRs and CRs for Nanopore sequencing were compared to Illumina WGBS read coverage distributions with and without multi-mappers (bam to coverage in-house python script).

Characterization of genome-wide methylation

1 kb tiling windows were calculated using BEDtools makewindow with parameter -w 1000. The average methylation level of all 1 kb windows was calculated using UCSCtools bigWigAverageOverBed and filtered for a minimum coverage of 5 CpGs. The methylation rate distribution of the DKO_0 and TKO_L was visualized using the density plot function of ggplot2. The methylation difference in these windows between the DKO_0 and TKO_L was calculated and visualized using the empirical cumulative density function of the ggplot2 package. The genomic distribution of DMRs was visualized using the *Cis*-regulatory Element Annotation System (CEAS)[23].

Correlation of methylation rates in 5 kb windows and IAPs

5 kb tiling windows were calculated using BEDtools makewindow with parameter -w 5000. The average methylation level of all 5 kb windows not overlapping an IAP as well as for all IAP elements were calculated using UCSCtools bigWigAverageOverBed and filtered for a minimum coverage of 10 CpGs. For visualization the R smoothScatter function was used.

Split violin plots for DMRs and CRs

Genome-wide methylation information was filtered by sample to CpGs within DMRs or CRs. The single CpG resolution methylation values were visualized in R using the vioplot package (parameter side=left/right).

Read distribution in MeDIPseq

SAMtools view was used to calculate the number of reads in DMRs/CRs (parameter -c -F 256 -F4). CR counts were divided by 1,000 to account for their overrepresentation. Boxplots were used to display the mean number of reads.

Profile plots and enrichment heatmaps (ChIP-seq/MeDIP-seq)

The computeMatrix function of the deepTools package in scale-regions mode with parameters –missingDataAsZero binSize=50 or 100 was used to calculate the input for the plotHeatmap function. Missing data color was set to white.

Overlap of DMRs and CRs with genomic element

Genomic regions were each first collapsed using BEDtools merge to avoid counting regions twice, i.e., overlapping repeat classes within a family. Next, for each genomic element the overlap with DMRs (CRs) was calculated, normalized by the total length of DMRs (CRs) and visualized using ggplot2. For enrichment of DMRs a two-sample Wilcoxon test was used to compare DMR and CR overlaps (alternative='greater') with the respective elements.

Fold-change of hairpin bisulfite derived CpG methylation status

Hairpinanalyzer output was further used to visualize the change in CpG status composition per target. The fraction of CpGs that are fully methylated, hemi-methylated on plus- or minus-strand or fully unmethylated were normalized by TKO_L values for DKO_0 P1, DKO_0 P5 and WT. Log2-fold change of these normalized values was plotted using ggplot2.

Unique vs. multiple mapper analysis

Alignment statistics of Illumina sequenced samples for DMRs (CRs) were obtained using SAMtools view with parameters -c (count) -F 4 (exclude unmapped reads) and -F/-f 256 (unique/multiple alignments) and -L DMRs/CRs (reduce to regions). For CRs the average per replicate was used to calculate the log2 fold-change of unique (multiple) alignments between DMRs and CRs. A paired Wilcoxon test was used to test if unique (multiple) alignments show a different prevalence in DMRs compared to CR. Nanopore reads are by default only aligned uniquely, therefore the distribution of DMR vs. CR alignment rates (log2 fold-change) is only shown for unique alignments.

Gene set enrichment analysis of mass spectrometry experiments

Genes that were detected as significantly enriched in DKO_0 and TKO_L Uhrf1-FLAG Mass Spec experiment were used as input for the WEB-based Gene SeT AnaLysis Toolkit (WebGestalt) to calculate Gene Set Enrichment Analysis (gene ontology: cellular components)[24].

ERVK, methylation and Uhrf1, Trim28 and H3K9me3 enrichment at DMRs

The heatmap visualizing the overlap with ERVKs, the binding signal of Uhrf1, Trim28 and H3K9me3 as well as DMR was plotted using the R package EnrichedHeatmap. Single nucleotide resolution bigWig files and the ERVK annotation file were loaded to R and for each sample the normalizeToMatrix function (w=250; methylation: mean_mode='absoltute', background=NA; Uhrf1, Trim28 and H3K9me3: mean_mode='coverage', background=0) was used to calculate the signal across DMRs.

IAPEz-int methylation comparison of E3.5 and E6.5

The mean DMR methylation rate of each E3.5 and E6.5 embryo conditions was calculated and visualized using the R package ComplexHeatmap. The color key was set display the center color to 0.15 to allow for visualization of expected methylation rate dynamics.

References

1. Giesselmann, P., Hetzel, S., Muller, F.J., Meissner, A. & Kretzmer, H. Nanopype: a modular and scalable nanopore data processing pipeline. *Bioinformatics* **35**, 4536 (2019).
2. Li, H. Minimap2: pairwise alignment for nucleotide sequences. *Bioinformatics* **34**, 3094-3100 (2018).
3. Sedlazeck, F.J. *et al.* Accurate detection of complex structural variations using single-molecule sequencing. *Nat Methods* **15**, 461-468 (2018).
4. Simpson, J.T. *et al.* Detecting DNA cytosine methylation using nanopore sequencing. *Nat Methods* **14**, 407-410 (2017).
5. Xi, Y. & Li, W. BSMAP: whole genome bisulfite sequence MAPping program. *BMC Bioinformatics* **10**, 232 (2009).
6. Sun, D. *et al.* MOABS: model based analysis of bisulfite sequencing data. *Genome Biol* **15**, R38 (2014).
7. Lutsik, P. *et al.* BiQ Analyzer HT: locus-specific analysis of DNA methylation by high-throughput bisulfite sequencing. *Nucleic Acids Res* **39**, W551-6 (2011).
8. Kyriakopoulos, C., Giehr, P. & Wolf, V. H(O)TA: estimation of DNA methylation and hydroxylation levels and efficiencies from time course data. *Bioinformatics* **33**, 1733-1734 (2017).
9. Hoffmann, S. *et al.* A multi-split mapping algorithm for circular RNA, splicing, trans-splicing and fusion detection. *Genome Biol* **15**, R34 (2014).
10. Hoffmann, S. *et al.* Fast mapping of short sequences with mismatches, insertions and deletions using index structures. *PLoS Comput Biol* **5**, e1000502 (2009).

11. Otto, C., Stadler, P.F. & Hoffmann, S. Fast and sensitive mapping of bisulfite-treated sequencing data. *Bioinformatics* **28**, 1698-704 (2012).
12. Lienhard, M. *et al.* QSEA-modelling of genome-wide DNA methylation from sequencing enrichment experiments. *Nucleic Acids Res* **45**, e44 (2017).
13. H., L. Aligning sequence reads, clone sequences and assembly contigs with BWA-MEM. *arXiv* (2013).
14. McKenna, A. *et al.* The Genome Analysis Toolkit: a MapReduce framework for analyzing next-generation DNA sequencing data. *Genome Res* **20**, 1297-303 (2010).
15. Zhang, Y. *et al.* Model-based analysis of ChIP-Seq (MACS). *Genome Biol* **9**, R137 (2008).
16. Li, H. *et al.* The Sequence Alignment/Map format and SAMtools. *Bioinformatics* **25**, 2078-9 (2009).
17. Quinlan, A.R. & Hall, I.M. BEDTools: a flexible suite of utilities for comparing genomic features. *Bioinformatics* **26**, 841-2 (2010).
18. Kuhn, R.M., Haussler, D. & Kent, W.J. The UCSC genome browser and associated tools. *Brief Bioinform* **14**, 144-61 (2013).
19. Gu, Z., Eils, R. & Schlesner, M. Complex heatmaps reveal patterns and correlations in multidimensional genomic data. *Bioinformatics* **32**, 2847-9 (2016).
20. Gu, Z., Eils, R., Schlesner, M. & Ishaque, N. EnrichedHeatmap: an R/Bioconductor package for comprehensive visualization of genomic signal associations. *BMC Genomics* **19**, 234 (2018).
21. Ramirez, F., Dundar, F., Diehl, S., Gruning, B.A. & Manke, T. deepTools: a flexible platform for exploring deep-sequencing data. *Nucleic Acids Res* **42**, W187-91 (2014).
22. Thorvaldsdottir, H., Robinson, J.T. & Mesirov, J.P. Integrative Genomics Viewer (IGV): high-performance genomics data visualization and exploration. *Brief Bioinform* **14**, 178-92 (2013).
23. Ji, X., Li, W., Song, J., Wei, L. & Liu, X.S. CEAS: cis-regulatory element annotation system. *Nucleic Acids Res* **34**, W551-4 (2006).
24. Wang, J., Vasaikar, S., Shi, Z., Greer, M. & Zhang, B. WebGestalt 2017: a more comprehensive, powerful, flexible and interactive gene set enrichment analysis toolkit. *Nucleic Acids Res* **45**, W130-W137 (2017).
