# Supplementary material for: Dnmt1 has de novo activity targeted to transposable elements
Source: Nat Struct Mol Biol. 2021 Jun 17;28(7):594–603. doi: 10.1038/s41594-021-00603-8 (PMC8279952; doi:10.1038/s41594-021-00603-8)
Supplement: Supplementary file 2 — Reporting Summary [file 41594_2021_603_MOESM2_ESM.pdf]

nature research

Corresponding author(s): Alexander Meissner

Last updated by author(s): May 5, 2021

Reporting Summary

Nature Research wishes to improve the reproducibility of the work that we publish. This form provides structure for consistency and transparency in reporting. For further information on Nature Research policies, see our [Editorial Policies](#) and the [Editorial Policy Checklist](#).

Statistics

For all statistical analyses, confirm that the following items are present in the figure legend, table legend, main text, or Methods section.

n/a	Confirmed	
☐	☒	The exact sample size (*n*) for each experimental group/condition, given as a discrete number and unit of measurement
☐	☒	A statement on whether measurements were taken from distinct samples or whether the same sample was measured repeatedly
☐	☒	The statistical test(s) used AND whether they are one- or two-sided<br>*Only common tests should be described solely by name; describe more complex techniques in the Methods section.*
☒	☐	A description of all covariates tested
☐	☒	A description of any assumptions or corrections, such as tests of normality and adjustment for multiple comparisons
☐	☒	A full description of the statistical parameters including central tendency (e.g. means) or other basic estimates (e.g. regression coefficient) AND variation (e.g. standard deviation) or associated estimates of uncertainty (e.g. confidence intervals)
☐	☒	For null hypothesis testing, the test statistic (e.g. *F*, *t*, *r*) with confidence intervals, effect sizes, degrees of freedom and *P* value noted<br>*Give P values as exact values whenever suitable.*
☒	☐	For Bayesian analysis, information on the choice of priors and Markov chain Monte Carlo settings
☒	☐	For hierarchical and complex designs, identification of the appropriate level for tests and full reporting of outcomes
☐	☒	Estimates of effect sizes (e.g. Cohen's *d*, Pearson's *r*), indicating how they were calculated

Our web collection on statistics for biologists contains articles on many of the points above.

Software and code

Policy information about availability of computer code

Data collection	Zeiss Axiocam 506 images were processed in ZEN 3.2, Zeiss LSM880 confocal microscope images were processed with FIJI (ImageJ, 2.1.0/1.53i).
Data analysis	Nanopype pipeline (v1.1.0), Guppy (v4.0.11), minimap2 (v2.10), Sniffles (v1.0.10), NGMLR (v0.2.7), nanopolish (v0.13.2), BSmap (v2.9.0), FastQC(v0.11.8), cutadapt(v2.4), MOABS mcall module (v1.3.2), segemehl (v0.2.0), QSEA (1.12.0), BWA mem (v0.7.17), GATK (v4) , MACS2 (2.1.2_dev), Hairpinanalyzer (v0.3), IGV (v2.9.2), SAMtools (v1.10), BEDtools (v2.25.0), UCSCtools (v4), R Complex Heatmap (v1.99.5), Enriched Heatmap (1.19.2), deepTools (v.2.5.4), R (v3.6.1), vioplot (v0.3.5), ggplot (3.3.2), and Perseus(1.6.6.0)

For manuscripts utilizing custom algorithms or software that are central to the research but not yet described in published literature, software must be made available to editors and reviewers. We strongly encourage code deposition in a community repository (e.g. GitHub). See the Nature Research guidelines for submitting code & software for further information.

Data

Policy information about availability of data

All manuscripts must include a data availability statement. This statement should provide the following information, where applicable:

- Accession codes, unique identifiers, or web links for publicly available datasets
- A list of figures that have associated raw data
- A description of any restrictions on data availability

All sequencing data have been deposited in the Gene Expression Omnibus (GEO) under accession code GSE158460 (secure token to allow review while it remains in private status:olejmcqwtdozxan). In vivo mouse embryo WGBS data from our own publications were used for the initial in vivo comparisons. This data is available from Gene Expression Omnibus under accession code GSE137337 and GSE84235. CpG island (CGI) and Repeat Masker tracks were downloaded for mm9 from the UCSC genome browser. Encode histone modifications (H3K4me1, H3K4me3, H3K27me3, H3K9me3, H3K27ac, H3K36me3, H3K9ac) were downloaded from NCBI

(GSE31039). Gene annotation was downloaded from Encode for mm9 and filtered for protein-coding genes. Mass spectrometry data is available on PRIDE using the accession PXD025736. Source Data for figures is deposited online at https://doi.org/10.6084/m9.figshare.14555250. Code is available at https://github.com/HeleneKretzmer/Dnmt1_de_novo

Field-specific reporting

Please select the one below that is the best fit for your research. If you are not sure, read the appropriate sections before making your selection.

☒ Life sciences ☐ Behavioural & social sciences ☐ Ecological, evolutionary & environmental sciences

For a reference copy of the document with all sections, see nature.com/documents/nr-reporting-summary-flat.pdf

Life sciences study design

All studies must disclose on these points even when the disclosure is negative.

Sample size

No statistical methods were used to predetermine sample sizes and are indicated in the figure panels or legends. Sample sizes for qPCR, Mass Spectroscopy, ChIP-seq, WGBS, and other sequencing technologies are consistent with the current standards for sample sizes and included controls in the published literature. The number of embryos reported is the maximal number of embryos we could recover and reasonably submit for WGBS (E6.5 = 3, E3.5 = 20).

Data exclusions

Blastocysts were scored for morphology, size, and viability with any embryos showing delayed development excluded from retransfer. In/exclusion criteria for morphometric analysis are specified in Methods section under "Dnmt1 inhibition and recovery in vivo.
In/exclusion criteria for in vivo and DMR analysis are specified in Supplementary Note on Computational Methods section. Briefly, DMRs were filtered based on Nanopore long reads from the same cell line by stringently filtering on methylation difference as measured from Nanopore read data. Only DMRs that had at least 10 informative CpG positions were subject to filtering and any DMR with a mean difference between the DKO0 and TKOL samples of less than 0.05 was excluded.

Replication

All replication attempts for this study were successful. Findings were replicated across two different Dnmt KO cell lines with separate Dnmt1 rescue strategies. Both systems show similar effects upon Dnmt1 re-expression and target regions are highly reproducible. Uhrf1 KO studies involved two separate KO clones with different targeting strategies, with both lines showing similar extreme hypomethylated phenotype. All ChIP-seq assays were run in duplicate, except H3K36me3 n=1, and correlation was compared to WT V6.5 mESC and between replicates. All in vivo WGBS assays represent a pool of embryos (E6.5 = 3, E3.5 n = WT 8, Dnmt1 KO 12, DKO 20, DKODMSO 8, DKODnmt1i 8 embryos). Mass spec samples were run in replicates of 3 and compared to untagged control samples also run in triplicate. QPCR assays were run in biological triplicate. Hairpin bisulfite analysis was run once for each primer pair in each cell line. Westerns were run once but corroborated by different targeting strategies in the TKOL and TKO with ectopic Dnmt1 and Uhrf1 KO subclones or genotyping. MeDIP was run in triplicate for each time point. The EB experiments constitute a pool of exactly 100 EBs and use random sampling to remove any operator bias. PromethION runs were performed once for each sample due to high coverage depth.

Randomization

Cell culture samples for every experiment were collected without a preconceived selection strategy. Embryos for every experiment were collected without a preconceived selection strategy or prioritization by morphology.

Blinding

No blinding was carried out as it was not relevant for the strategies used in this study. However, our analytical pipeline followed uniform criteria applied to all samples, allowing us to analyze our data in an unbiased manner.

Reporting for specific materials, systems and methods

We require information from authors about some types of materials, experimental systems and methods used in many studies. Here, indicate whether each material, system or method listed is relevant to your study. If you are not sure if a list item applies to your research, read the appropriate section before selecting a response.

Materials & experimental systems

n/a	Involved in the study
☐	☒ Antibodies
☐	☒ Eukaryotic cell lines
☒	☐ Palaeontology and archaeology
☐	☒ Animals and other organisms
☒	☐ Human research participants
☒	☐ Clinical data
☒	☐ Dual use research of concern

Methods

n/a	Involved in the study
☐	☒ ChIP-seq
☒	☐ Flow cytometry
☒	☐ MRI-based neuroimaging

Antibodies

Antibodies used

H3K9me3 (2ug ChIP-seq; Abcam, ab8898), H3K4me3 (2ug ChIP-seq; Abcam, ab8580), H3K36me3 (2ug ChIP-seq; Active Motif, 61101), 5mC (1:33 MeDIP; Diagenode, C15410205-50), FLAG (1ug for ChIPmentation and 10ug for RIME; Sigma, F1804), Uhrf1 (1:250 western blot; Santa-Cruz, sc-373750), Dnmt1 (1:1,000 western blot; Abcam, ab87654), GAPDH (1:1,000 western blot; Cell Signalling,

	14C10), Trim28 (1:1,000 for western blot and 5ug for ChIP-seq; Abcam, ab22553), Lamin-B (1:1,000 western blot; Abcam, ab8982), Beta Actin (1:1,000 western blot; Abcam, ab8226), and HRP secondary (1:10,000 western blot; Jackson Laboratory # 115-035-174 or 211-032-171)
Validation	Antibodies were validated against WT V6.5 mESCs in both western blot or ChIP-seq assays.

Eukaryotic cell lines

Policy information about cell lines

Cell line source(s)	The TKOL cell line was originally published in Meissner et al., 2005 and the WT KH2 lines were those published in Beard et al., 2006. Both were from the original lines used in the publication and obtained from Alexander Meissner at the time of their creation. Information on cell line generation provided in Methods under: “DKO0 cell line generation”, “TKO cell line generation”, “Uhrf1 FLAG line generation”, “Uhrf1 KO line generation”, and “Dnmt1 rescue experiment”, V6.5 cell RRID = CVCL_C865. Wild-type V6.5 mouse embryonic stem cell line was provided by the lab of Konrad Hochedlinger.
Authentication	Dnmt1 protein expression was validated by western blot and/or methylation assayed by WGBS for TKOL, DKO0, TKO, TKO +Dnmt1, TKO + catalytic inactive Dnmt1, TKOL Uhrf1 KO, DKO0 Uhrf1 KO, and V6.5 WT
Mycoplasma contamination	Cell lines tested negative for mycoplasma.
Commonly misidentified lines (See ICLAC register)	None used.

Animals and other organisms

Policy information about studies involving animals; ARRIVE guidelines recommended for reporting animal research

Laboratory animals	All mice kept under SPF-Conditions in individually ventilated cages at a temperature of 22°C +/- 2 °C and a humidity of 55% +/- 10% with a 12hr light/dark cycle (7am to 7pm). Oocytes were isolated from B6D2F1 strain female mice (age 7 to 9 weeks, Envigo), sperm was isolated from B6/CAST F1 male mice (>2 months of age) which were generated previously by breeding C57BL/6J strain female mice with CAST/EiJ strain males (reported in Grosswendt et al., 2020). Blastocysts were transferred into Hsd:ICR (CD-1) strain female mice (age 8-[illegible]12 weeks 30-35g, Envigo) which had been mated with Vasectomized SW strain males (> 1 year of age, Envigo)
Wild animals	Wild animals were not involved in this study.
Field-collected samples	No samples were collected from the field.
Ethics oversight	All procedures have been performed in our specialized facility, and we followed all relevant animal welfare guidelines and regulations. Protocols were approved by Harvard University IACUC protocol (28-21) and the Max Planck Institute for Molecular Genetics (G0247/13-SGr1).

Note that full information on the approval of the study protocol must also be provided in the manuscript.

ChIP-seq

Data deposition

☒ Confirm that both raw and final processed data have been deposited in a public database such as GEO.

☒ Confirm that you have deposited or provided access to graph files (e.g. BED files) for the called peaks.

Data access links *May remain private before publication.*	All sequencing data have been deposited in the Gene Expression Omnibus (GEO) under accession code GSE158460 (secure token to allow review while it remains in private status:olejmcqwtdozxan).
Files in database submission	DKOzero_H3K9me3.broadPeak.bed<br>DKOzero_Trim28.broadPeak.bed<br>DKOzero_Uhrf1.broadPeak.bed<br>DKOzero_Uhrf1_p3_rep2.broadPeak.bed<br>DKOzero_p3_Uhrf1_rep1.broadPeak.bed<br>DKOzero_p5_rep1_H3K9me3.broadPeak.bed<br>DKOzero_p5_rep1_p5Cre_Trim28.broadPeak.bed<br>DKOzero_p5_rep2_H3K9me3.broadPeak.bed<br>DKOzero_p5_rep2_p5Cre_Trim28.broadPeak.bed<br>TKOlike_H3K9me3.broadPeak.bed<br>TKOlike_Trim28.broadPeak.bed<br>TKOlike_Uhrf1.broadPeak.bed<br>TKOlike_Uhrf1_rep1.broadPeak.bed<br>TKOlike_Uhrf1_rep2.broadPeak.bed<br>TKOlike_rep1_H3K9me3.broadPeak.bed<br>TKOlike_rep1_Trim28.broadPeak.bed<br>TKOlike_rep2_H3K9me3.broadPeak.bed<br>TKOlike_rep2_Trim28.broadPeak.bed<br>V6.5_Uhrf1_rep1.broadPeak.bed

V6.5_Uhrf1_rep2.broadPeak.bed
V65_rep1_H3K9me3.broadPeak.bed
V65_rep1_Trim28.broadPeak.bed
V65_rep2_H3K9me3.broadPeak.bed
V65_rep2_Trim28.broadPeak.bed
H3K9me3_WT_rep1.bw H3K9me3_WT_rep1_R1.fastq.gz H3K9me3_WT_rep1_R2.fastq.gz
H3K9me3_WT_rep2.bw H3K9me3_WT_rep2_R1.fastq.gz H3K9me3_WT_rep2_R2.fastq.gz
H3K9me3_DKO0_rep1.bw H3K9me3_DKO0_rep1_R1.fastq.gz H3K9me3_DKO0_rep1_R2.fastq.gz
H3K9me3_DKO0_rep2.bw H3K9me3_DKO0_rep2_R1.fastq.gz H3K9me3_DKO0_rep2_R2.fastq.gz
H3K9me3_TKOL_rep1.bw H3K9me3_TKOL_rep1_R1.fastq.gz H3K9me3_TKOL_rep1_R2.fastq.gz
H3K9me3_TKOL_rep2.bw H3K9me3_TKOL_rep2_R1.fastq.gz H3K9me3_TKOL_rep2_R2.fastq.gz
Uhrf_WT_rep1.bw Uhrf_WT_rep1_R1.fastq.gz Uhrf_WT_rep1_R2.fastq.gz
Uhrf_WT_rep2.bw Uhrf_WT_rep2_R1.fastq.gz Uhrf_WT_rep2_R2.fastq.gz
Uhrf_DKO0_rep1.bw Uhrf_DKO0_rep1_R1.fastq.gz Uhrf_DKO0_rep1_R2.fastq.gz
Uhrf_DKO0_rep2.bw Uhrf_DKO0_rep2_R1.fastq.gz Uhrf_DKO0_rep2_R2.fastq.gz
Uhrf_TKOL_rep1.bw Uhrf_TKOL_rep1_R1.fastq.gz Uhrf_TKOL_rep1_R2.fastq.gz
Uhrf_TKOL_rep2.bw Uhrf_TKOL_rep2_R1.fastq.gz Uhrf_TKOL_rep2_R2.fastq.gz
Trim28_WT_rep1.bw Trim28_WT_rep1_R1.fastq.gz Trim28_WT_rep1_R2.fastq.gz
Trim28_WT_rep2.bw Trim28_WT_rep2_R1.fastq.gz Trim28_WT_rep2_R2.fastq.gz
Trim28_DKO0_rep1.bw Trim28_DKO0_rep1_R1.fastq.gz Trim28_DKO0_rep1_R2.fastq.gz
Trim28_DKO0_rep2.bw Trim28_DKO0_rep2_R1.fastq.gz Trim28_DKO0_rep2_R2.fastq.gz
Trim28_TKOL_rep1.bw Trim28_TKOL_rep1_R1.fastq.gz Trim28_TKOL_rep1_R2.fastq.gz
Trim28_TKOL_rep2.bw Trim28_TKOL_rep2_R1.fastq.gz Trim28_TKOL_rep2_R2.fastq.gz
WT_Uhrf1_Input_rep1.bw WT_Uhrf1_Input_rep1_R1.fastq.gz WT_Uhrf1_Input_R2.fastq.gz
WT_Uhrf1_Input_rep2.bw WT_Uhrf1_Input_rep2_R1.fastq.gz WT_Uhrf1_Input_R2.fastq.gz
DKO0_Uhrf1_Input_rep1.bw DKO0_Uhrf1_Input_rep1_R1.fastq.gz DKO0_Uhrf1_Input_R2.fastq.gz
DKO0_Uhrf1_Input_rep2.bw DKO0_Uhrf1_Input_rep2_R1.fastq.gz DKO0_Uhrf1_Input_R2.fastq.gz
TKOL_Uhrf1_Input_rep1.bw TKOL_Uhrf1_Input_rep1_R1.fastq.gz TKOL_Uhrf1_Input_R2.fastq.gz
TKOL_Uhrf1_Input_rep2.bw TKOL_Uhrf1_Input_rep2_R1.fastq.gz TKOL_Uhrf1_Input_R2.fastq.gz

Genome browser session
(e.g. UCSC)

http://genome-euro.ucsc.edu/s/helene/Dnmt1_de_novo

Methodology

Replicates

2

Sequencing depth

Each individual library was sequenced to a target depth of 50 million reads

Antibodies

H3K9me3 (Abcam, ab8898), FLAG (Sigma, F1804), Trim28 (Abcam, ab22553)

Peak calling parameters

Peaks were called using the MACS2 (2.1.2_dev) peakcall function using default parameters.

Data quality

Raw data were inspected using FastQC, adapter and low quality bases were removed using cutadapt. Correlation of coverage BigWig files across replicates and conditions was used to check for reproducibility.

Software

The ChIP-seq sequencing data as well as the control input sequencing were aligned to the mouse mm9 reference genome using BWA mem using the default parameter. GATK was used to obtain alignment metrics and remove duplicates. Peaks were called using the MACS2 (2.1.2_dev) peakcall function using default parameters. After validation of replicate comparability and quality, replicates were merged on read level and reprocessed together with input samples. Background subtracted coverage files were obtained using MACS2 bdgcomp with -m FE.
