# Supplementary figures and images for: Dnmt1 has de novo activity targeted to transposable elements
Source: Nat Struct Mol Biol. 2021 Jun 17;28(7):594–603. doi: 10.1038/s41594-021-00603-8 (PMC8279952; doi:10.1038/s41594-021-00603-8)

Fig. 2a

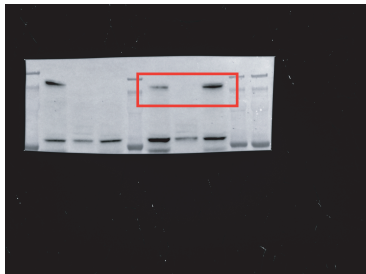

Fig. 2b

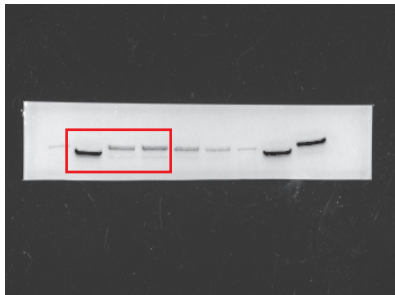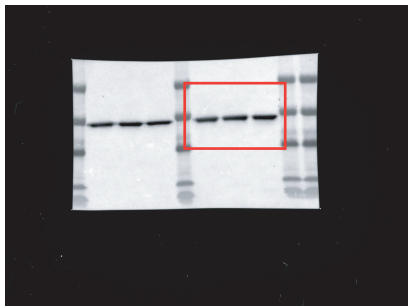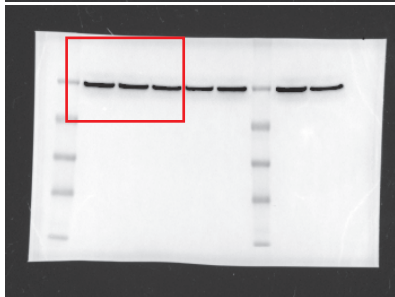

Supplement: Source Data Fig. 2 — Unprocessed western blots. [file 41594_2021_603_MOESM8_ESM.pdf]

Fig. 4a (left)

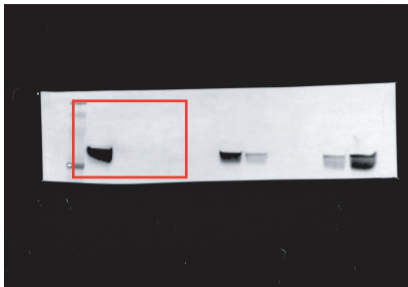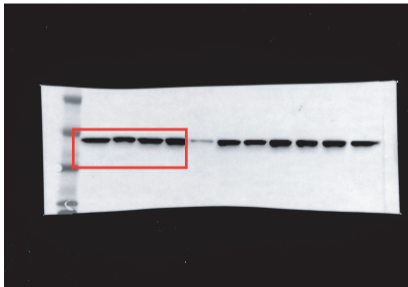

Fig. 4a (right)

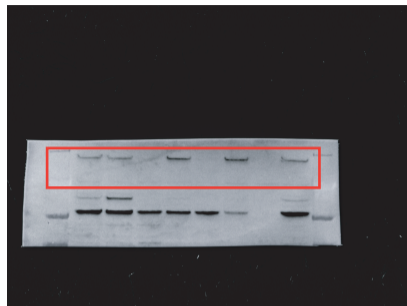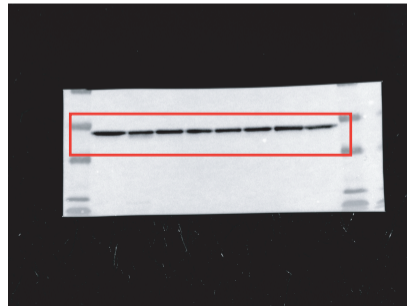

Supplement: Source Data Fig. 4 — Unprocessed western blots. [file 41594_2021_603_MOESM9_ESM.pdf]

ED 1a (left)

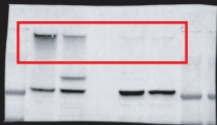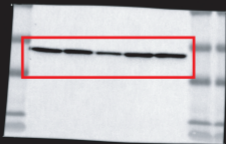

Supplement: Source Data Extended Data Fig. 1 — Unprocessed western blots. [file 41594_2021_603_MOESM10_ESM.pdf]

ED 5a (top)

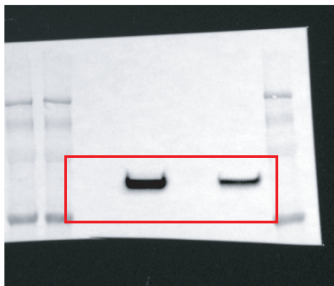

ED 5a (bottom)

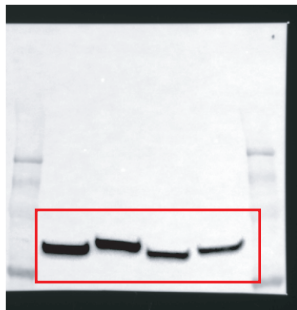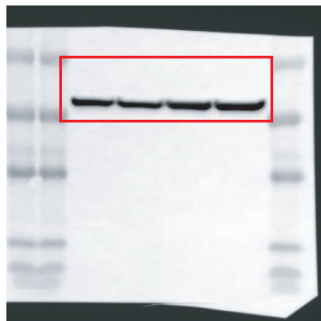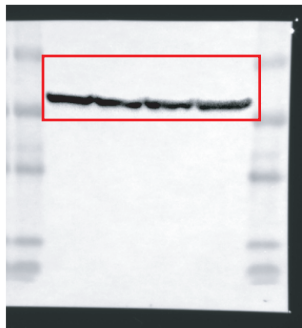

Supplement: Source Data Extended Data Fig. 5 — Unprocessed western blots. [file 41594_2021_603_MOESM11_ESM.pdf]
