# Supplementary material for: Dnmt1 has de novo activity targeted to transposable elements
Source: Nat Struct Mol Biol. 2021 Jun 17;28(7):594–603. doi: 10.1038/s41594-021-00603-8 (PMC8279952; doi:10.1038/s41594-021-00603-8)
Supplement: Supplementary file 3 — Peer Review Information [file 41594_2021_603_MOESM3_ESM.pdf]

natureresearch

Peer Review Information

Journal: Nature Structural and Molecular Biology
Manuscript Title: Dnmt1 has de novo activity targeted to transposable elements
Corresponding author name(s): Alexander Meissner

Reviewer Comments & Decisions:

Decision Letter, initial version:

18th Dec 2020

Dear Alex,

Thank you again for submitting your manuscript "Dnmt1 has de novo activity targeted to transposable elements". I apologize for the delay in reaching a decision, which resulted from the difficulty in obtaining referee reports. Nevertheless, we now have received comments from two reviewers, both experts in DNA methylation, who evaluated your paper (appended below). In light of those reports, we remain interested in your study and would like to invite you to respond to the comments of the referees, in the form of a revised manuscript.

You will see that both reviewers find the study interesting and well executed, but they also raise several concerns that should be addressed in the revision. Apart from several clarifications, the reviewers request additional experimental support for the proposed mechanism of genomic recruitment via Trim28 and H3K9me3 and reviewer #2 requires further evidence for the de novo activity of Dnmt1.

Please be sure to address/respond to all concerns of the referees in full in a point-by-point response and highlight all changes in the revised manuscript text file. If you have comments that are intended for editors only, please include those in a separate cover letter.

We are committed to providing a fair and constructive peer-review process. Do not hesitate to contact us if there are specific requests from the reviewers that you believe are technically impossible or unlikely to yield a meaningful outcome.

We expect to see your revised manuscript within 6-8 weeks. If you cannot send it within this time, please contact us to discuss an extension; we would still consider your revision, provided that no similar work has been accepted for publication at NSMB or published elsewhere.

As you already know, we put great emphasis on ensuring that the methods and statistics reported in our papers are correct and accurate. As such, if there are any changes that should be reported, please submit an updated version of the Reporting Summary along with your revision.

Please follow the links below to download these files:

Reporting Summary:
https://www.nature.com/documents/nr-reporting-summary.pdf

Please note that the form is a dynamic 'smart pdf' and must therefore be downloaded and completed in Adobe Reader.

When submitting the revised version of your manuscript, please pay close attention to our href="https://www.nature.com/nature-research/editorial-policies/image-integrity">Digital Image Integrity Guidelines. and to the following points below:

-- that unprocessed scans are clearly labelled and match the gels and western blots presented in figures.
-- that control panels for gels and western blots are appropriately described as loading on sample processing controls
-- all images in the paper are checked for duplication of panels and for splicing of gel lanes.

Finally, please ensure that you retain unprocessed data and metadata files after publication, ideally archiving data in perpetuity, as these may be requested during the peer review and production process or after publication if any issues arise.

Please note that all key data shown in the main figures as cropped gels or blots should be presented in uncropped form, with molecular weight markers. These data can be aggregated into a single supplementary figure item. While these data can be displayed in a relatively informal style, they must refer back to the relevant figures. These data should be submitted with the final revision, as source data, prior to acceptance, but you may want to start putting it together at this point.

DATA AVAILABILITY: this journal strongly supports public availability of data. All data used in accepted papers should be available via a public data repository, or alternatively, as Supplementary Information. If data can only be shared on request, please explain why in your Data Availability Statement, and also in the correspondence with your editor. Please note that for some data types, deposition in a public repository is mandatory - more information on our data deposition policies and available repositories can be found below:
https://www.nature.com/nature-research/editorial-policies/reporting-standards#availability-of-data

Deposition of deep sequencing data is mandatory, and the datasets must be released prior to or upon publication. To avoid delays in publication, dataset accession numbers must be supplied with the final accepted manuscript and appropriate release dates must be indicated at the galley proof stage.

SOURCE DATA: we urge authors to provide, in tabular form, the data underlying the graphical

representations used in figures. This is to further increase transparency in data reporting, as detailed in this editorial (http://www.nature.com/nsmb/journal/v22/n10/full/nsmb.3110.html). Spreadsheets can be submitted in excel format. Only one (1) file per figure is permitted; thus, for multi-paneled figures, the source data for each panel should be clearly labeled in the Excel file; alternately the data can be provided as multiple, clearly labeled sheets in an Excel file. When submitting files, the title field should indicate which figure the source data pertains to. We encourage our authors to provide source data at the revision stage, so that they are part of the peer-review process.

While we encourage the use of color in preparing figures, please note that this will incur a charge to partially defray the cost of printing. Information about color charges can be found at http://www.nature.com/nsmb/authors/submit/index.html#costs

Nature Structural & Molecular Biology is committed to improving transparency in authorship. As part of our efforts in this direction, we are now requesting that all authors identified as 'corresponding author' on published papers create and link their Open Researcher and Contributor Identifier (ORCID) with their account on the Manuscript Tracking System (MTS), prior to acceptance. This applies to primary research papers only. ORCID helps the scientific community achieve unambiguous attribution of all scholarly contributions. You can create and link your ORCID from the home page of the MTS by clicking on 'Modify my Springer Nature account'. For more information please visit please visit www.springernature.com/orcid.

Please use the link below to submit your revised manuscript and related files:

[REDACTED]

Note: This URL links to your confidential home page and associated information about manuscripts you may have submitted, or that you are reviewing for us. If you wish to forward this email to co-authors, please delete the link to your homepage.

We look forward to seeing the revised manuscript and thank you for the opportunity to review your work.

With kind regards,
Anke

Anke Sparmann, PhD
Senior Editor
Nature Structural and Molecular Biology
ORCID 0000-0001-7695-2049

Reviewers' Comments:

Reviewer #1:

Remarks to the Author:
In this manuscript by Haggerty et al. “Dnmt1 has de novo activity targeted to transposable elements” the authors explore the possibility that DNMT1, which is known as the maintenance DNA methytransferase enzyme, might have de novo methylation activity.

This is a very interesting study, well executed and written, that demonstrates that DNMT1, under certain conditions as it has previously been observed (in cancer context particularly) and here in early mouse development on repetitive elements, can have de novo activity. I enjoyed reading the manuscript, highly novel findings and it will be useful for the research community.

Some aspects of the data are somewhat unclear and would benefit from further explanations and/or confirmatory experimental data:

1. Control regions to compare DMRs to: What is the CG content of these random regions, the same/similar like for the DMRs? The supplementary methods section says that “only regions with sufficient
CpGs were allowed as a destination for BEDtools shuffle”. What is the definition of sufficient? Can the authors show a CG distribution plot for CR and DMR?

2. The Dnmt1 shRNA cell line (TKOL): is it stable over time? Do the authors have data to show the DNMT1 levels over many passages? Figure 1D doesn’t show that.

3. Can the authors clarify that for the Nanopore sequencing the same DMRs have been used as in Fig 1 and not a new DMR set resulting from Nanopore?

4. Fig 2d and e - What's the precision of 5mC detection by Nanopore? In fig 2e there are many positions that have neither unmethylated or methylated CpGs.

5. Fig 3a,b - Data doesn't indicate that DNMT1 protein was expressed in UHRF1 KO cells. There could be all sorts of reasons this might not happen. Also, could be strengthened by ectopic DNMT1 expression.

6. To generate the UHRF1-FLAG, I couldn’t find the sequence of “Uhrf1_FLAG_sg”, the suppl tables are not named as in text, not easy to navigate through.

7. To claim that Trim 28 might provide an interaction scaffold for UHRF1, the authors could KD Trim28 in DKO0 and test if remethylation happens, otherwise it’s not conclusive.

8. How can the authors explain the finding that the E6.5 recovery methylation level, which was modest, was able to suppress completely the expression of IAPEz in Fig 4f?

Minor points:

-not clear to a general reader what 0.05 means in this context “96% IAPs are less methylated, loss >0.05, in the Dnmt1 KO”, a non-specialist reader might understand 5%, 0.05% or a p-value, just add more detail to be clearer. Same for “The DMRs have a mean
methylation of 0.162”

- can the authors briefly explain why they used MeDIP (with all its inherent non-linear enrichment of DNA methylation) when they could have done WGBS? Especially since small methylation changes cannot be confidently called with MeDIP.

- It’s not very clear here, is n=4,708 excluded or remaining? Same with 13Mb “First, we directly excluded all CpGs from the DMR detection that are located in regions detected as a deletion in the TKOL or DKO0 but present in the reference genome (n = 4,708 regions, in total 13Mb).”

Reviewer #2:
Remarks to the Author:
In this study, Haggerty, Kretzmer et. al. provide a comprehensive dissection and analysis of Dnmt1 “de novo” methylation activity in early development. Through stringent genetic manipulations during early mouse development, Dnmt1 is demonstrated to have “de novo” methylation activity that is enriched at IAP retrotransposons. This finding is reinforced through the novel use of long-read Nanopore sequencing to ensure the Dnmt1 dependent methylation at repetitive elements by significantly increasing the coverage of uniquely mapped reads at repetitive regions of the genome. The authors additionally demonstrate that Dnmt1 “de novo” activity is reliant on the presence of the cofactor Uhrf1. Using mass spectrometry, it is demonstrated that Uhrf1 interacts with the heterochromatic scaffolding protein, Trim28, and the authors hypothesize that this interaction coupled with association of H3K9me3 helps direct Uhrf1, and subsequently Dnmt1, to IAP retrotransposons for “de novo” methylation deposition. Finally, using in vivo mouse models, the authors demonstrate that “de novo” methylation activity of Dnmt1 is required to effectively repress IAPz-gag expression.

Overall, the study provides rigorous evaluation of Dnmt1 methylation activity in early development which is currently an active area of research. The in vitro, in vivo, and extensive genome-wide DNA methylation sequencing provide strong evidence for the role of Dnmt1 in silencing IAP retrotransposons during development by DNA methylation. However, a few aspects of the conclusions need to be addressed before the study is suitable for publication at NSMB:

Major Comments:

- The genetic experiments in Figure 1 and Figure 2 strongly suggest that Dnmt1 plays a major role in suppressing IAP retrotransposons in early development by DNA methylation. However, the ability to call this methylation “de novo” activity by Dnmt1 requires further evidence. For example, in Figure 1j, while DNA methylation is very low in the TKOL cells, there does appear to be a small amount of DNA methylation remaining, particularly at regions that are later deemed “de novo” methylated by Dnmt1 in later passages. Potentially, the small amount of DNA methylation that remains in a subpopulation of cells may serve as a template for Dnmt1 to perform traditional “maintenance” methylation (e.g., activity toward hemi-methylated DNA substrates), and the increase in DNA methylation at the later passages is a result of outgrowth of this subpopulation. To firmly establish that this gain in methylation is due to “de novo” activity of Dnmt1, performing strand-specific analyses like hairpin bisulfite sequencing would help demonstrate that Dnmt1 is acting in a de novo manner (e.g., methylating a fully unmethylated DNA substrate) at the identified genomic regions.

- While the results support the hypothesis that Uhrf1 is needed for Dnmt1 activity at these regions,

the rationale for the mechanism by which Uhrf1 and Dnmt1 are recruited is speculative at best. The authors hypothesize that since Trim28 interacts with Uhrf1, Trim28 aids in the deposition of H3K9me3 providing a means of recruitment for Uhrf1 through its histone binding domains. However, recent work suggests that H3K9me3 is dispensable for Uhrf1 role in DNA methylation maintenance (PMID: 27554592; PMID: 33097091; PMID: 30956060). The authors strongly hint at this mechanism by stating there is "co-localization" and "association" with H3K9me3 and Trim28; however, there is no functional evidence that demonstrates the requirement of Trim28 and H3K9me3 for Uhrf1 recruitment at these genomic locations.

Minor comments:

- The mass spectrometry results in Figure 3f seem to be selective of interactions that support the hypothesis; however, there appear to be several other protein interactions with Uhrf1 that are not discussed.

- It is an overstatement to say that the enrichment of H3K9me3 in DMRs of interest indicates that it has an impact on DNMT1 activity as stated in the text.

- The physiologic/pathologic relevance of this finding is unclear.

Author Rebuttal to Initial comments

We thank the reviewers for their feedback and constructive suggestions on how to improve this study. Please find below a summary of the additional experiments we conducted based on the reviewers feedback. A more detailed description and answers are given below in the point-by-point response. Major edits in the main text are marked in blue.

- We added **Hairpin bisulfite sequencing on TKO_L, DKO_0 P1 and P5 and WT** (Amplicon on several repeat classes) that shows an increase in hemi-methylated sites at IAPEz-int elements upon Dnmt1 re-expression, which is in line with the focal gain of methylation at these regions.
- We also used the resulting hairpin bisulfite data as input for **an HMM that predicts *de novo* and maintenance methylation's efficiencies** based on the observed levels of strand-specific methylation over the three time points. The model predicts increased *de novo* efficiency upon Dnmt1 re-expression, which is in line with the gain we observe in our DKO_0 WGBS series.
- We sorted (FACS) single cells from a DKO_0 P1 population, passaged them until P5, and then assayed methylation at IAPEz elements with amplicon bisulfite sequencing to show that **methylation gain at DMRs is not only occurring in a subpopulation of cells**.
- Based on the known and essential role of Trim28, a simple knockout would not work as any disruption of Trim28 is lethal even in mESCs. In order to circumvent this, we considered inducible shRNA knockdown, conditional knockout, and Fkbp degron integration. Ultimately, due to limitations with the other techniques (in detail discussed in the point-by-point), we designed a **Trim28 degron system** to analyze the dependency of Dnmt1 on Trim28 as a scaffold. However, despite these efforts, we found the lethality of Trim28 degradation is severe and manifests within a few days. Based on our various time points (see Figure 2), we would expect that at least 1-2 weeks

are needed before a meaningful gain of methylation occurs. Therefore, we **restructured the recruitment mechanism section** more carefully to present our ChIP-Seq and MassSpec results and the corresponding literature as support.

- We validated that the disruption of Uhrf1 by sgRNA and Cas9 did not alter the **presence or re-expression of Dnmt1 in the DKO_0 Uhrf1 KO by Western blot** analysis.
- To demonstrate **the stability of the Dnmt1 repression in the TKO_L cell line** we added a Western blot as well as WGBS comparing the initial TKO_L to TKO_L cells passaged for additional 5 or 10 additional passages.
- We added read-level methylation analysis for the *in vivo* experiments, showing a substantial number of cells/reads (80%) with at least partially methylated reads in the Dnmt3a/3b DKO Dnmt1 inhibitor-treated recovery sample at E6.5 Epiblast.
- We created an additional set of **CpG density matched of CRs,** to exclude that the enrichment of DMRs in IAPEz-ints is a result of CpG density underlying our DMR and CR definition.
- We included a GO-term analysis to better represent the complete set of proteins enriched in the **Uhrf1-FLAG mass spec**.
- To benchmark the **precision of 5mC identification by Nanopore,** we compared this to our WGBS data and found that correlation of WT methylation levels measured by Nanopore and WGBS showed good concordance between the two methodologies.

In addition, we further improved the following data:

- Increased the sequencing depth of the **Nanopore data to an average of 82X** and performed read-level methylation analysis.
- We extended the passaging time for the **DKO_0 by adding WGBS for P25**, which shows a further increase in methylation at DMRs and but also the genomic background.
- We performed our **EB time course with the DKO_0 P25** cells and further refined the methods we used to detect IAPEz-gag RNA-FISH and process the images.
- Additional **whole embryo IAPEz-gag RNA FISH was performed on WT, Dnmt1 KO, Dnmt3a/3b DKO, and DKO^{Dnmt1i} E8.5 embryos** to assess IAPEz repression in a later developmental context and show continued silencing in Dnmt3a/3b DKO and DKO^{Dnmt1i} embryos while Dnmt1 KO embryos show high levels of IAPEz-gag transcripts.

Reviewers' Comments:

Reviewer #1:

Remarks to the Author:

In this manuscript by Haggerty et al. “Dnmt1 has de novo activity targeted to transposable elements” the authors explore the possibility that DNMT1, which is known as the maintenance DNA methytransferase enzyme, might have de novo methylation activity.

This is a very interesting study, well executed and written, that demonstrates that DNMT1, under certain conditions as it has previously been observed (in cancer context particularly) and here in early mouse development on repetitive elements, can have de novo activity. I enjoyed reading the manuscript, highly novel findings and it will be useful for the research community.

We appreciate the positive feedback and the very helpful, constructive suggestions below.

Some aspects of the data are somewhat unclear and would benefit from further explanations and/or confirmatory experimental data:

1. Control regions to compare DMRs to: What is the CG content of these random regions, the same/similar like for the DMRs? The supplementary methods section says that “only regions with sufficient CpGs were allowed as a destination for BEDtools shuffle”. What is the definition of sufficient? Can the authors show a CG distribution plot for CR and DMR?

Response: We thank the reviewer for raising this point and added a more detailed description of the CR generation method to the Supplement (Section: “Control regions”). In brief, we merged all CpGs covered by the TKO_L and DKO_0 samples with a maximum distance of 2.5kb. All resulting regions with a minimum of 10 CpGs (which matches the DMRs, so it was called sufficient) were allowed as a location for CR.

This resulted in a CpG density closely resembling the genome-wide distribution (**Extended Data Figure 1d**) but lower than for the DMRs. However, given that CpG rich regions are frequently unmethylated (e.g., CGIs), we felt that an actual random distribution resembling the genome would allow for a more unbiased comparison.

Action taken: To ensure that no bias against LTRs was introduced, we created an additional set of CR matching the CpG density of the DMRs (CR^{CpG}) and compared their methylation and enrichment with the DMRs. CR^{CpG} regions show similar methylation dynamics to the previous CR (mean methylation in the DKO_0 P15 of 0.045 and 0.056, respectively) but contain an average number of CpGs close to that of the DMRs (DMRs 40, CR 12, CR^{CpG} 47). These alternative CpG density adjusted CR show a feature enrichment similar to the random CR, with a slightly higher enrichment in CpG rich features like TSS and CGIs, as well as SINEs, but not LTRs. Therefore, we decided to keep the random CR distribution to not artificially enrich for TSS and CGIs.

We included this comparison as a **new Extended Data Figure 1d and 2b**. and updated the Methods and text on page 4.

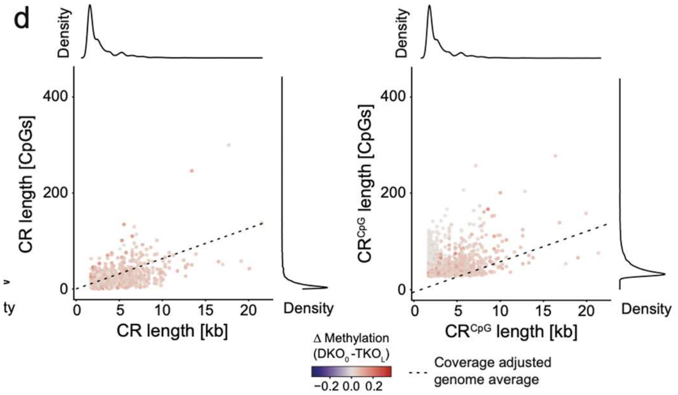

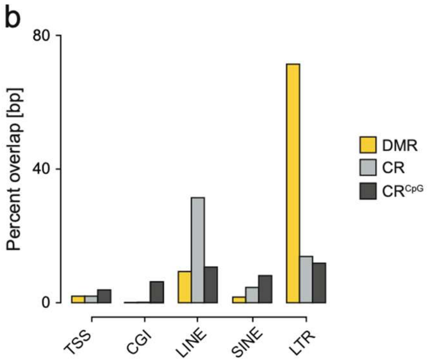

2. The Dnmt1 shRNA cell line (TKOL): is it stable over time? Do the authors have data to show the DNMT1 levels over many passages? Figure 1D doesn't show that.

Response: We agree that this information should be included. In addition, we would like to note that we also used the complete (true) TKO with no DNMT activity left (see below).

Action taken: We now added the passage information to the legend of the respective panel and a western blot of TKO_L cells passage 1, 5 and 10 that shows continued repression of Dnmt1 (**new Extended Data Figure 1a, left**). Furthermore, we now updated the figures by moving the complete TKO line (which retains no DNMT activity) and the ectopic rescue cell lines (including the catalytic active and inactive versions) that show hypermethylation of the same regions into the main figures (**Fig. 2 b, c, g**). The latter cannot be explained by any endogenous residual DNMT1 activity and together with the knockdown experiments strengthens the conclusion independently.

To further validate that we have a stable methylation landscape even for the later passage TKO_L, we performed WGBS for passage 10 and found DMR as well as global and CR methylation levels matching the TKO_L data used throughout the manuscript (**new Extended Data Figure 1a, right**).

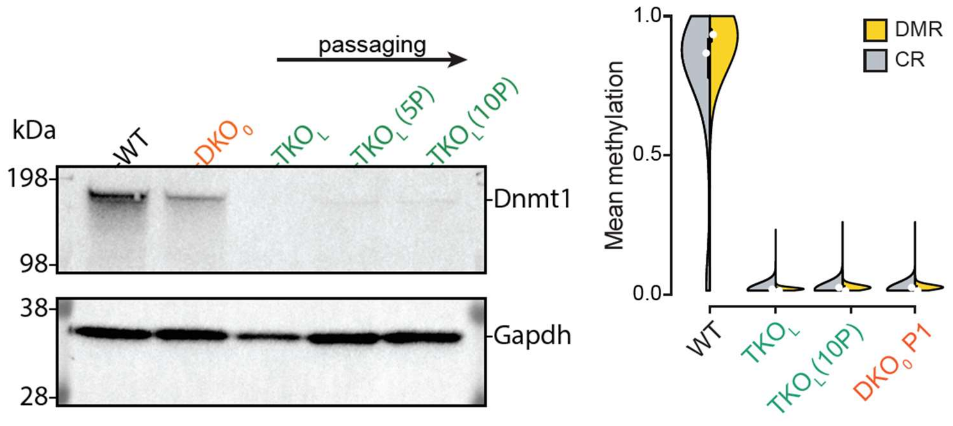

3. Can the authors clarify that for the Nanopore sequencing the same DMRs have been used as in Fig 1 and not a new DMR set resulting from Nanopore?

Response: We are glad the reviewer asked this question and would like to apologize that this was not clear enough. We used the same WGBS called DMR regions as seen in **Fig. 2** and validated these using Nanopore sequencing (**Fig. 3**). Throughout the rest of the manuscript, we only used the subset of WGBS defined DMRs that we could also validate using Nanopore data. The DMR numbers are as follows: n = 2,573 and after filtering with Nanopore reads: n = 1,515.

Action taken: We now changed the paragraph on page 6 of the manuscript to stress our validation approach and the nature of the DMRs. Since we only kept DMRs we could validate with Nanopore sequencing, we excluded 1,058 DMRs to make our set of methylation gaining regions as reliable as possible. This final set now includes 1,515 (see page 6) instead of 2,573 (see page 6) and is used for the remainder of the manuscript.

4. Fig 2d and e - What's the precision of 5mC detection by Nanopore? In fig 2e there are many positions that have neither unmethylated or methylated CpGs.

Response: The reviewer correctly points out that the number of missing values in the panel needs further explanation. We applied a stringent quality-cutoff (log-likelihood ratio >2.5) for calling a CpG on read-level as methylated or unmethylated, to only rely on confident calls and in exchange accepted missing values within reads on low confidence calls. On average, using this cutoff, we call ~60% of the CpGs per read, while the remaining CpGs are considered missing values. We now also further increased the sequencing depth, recovering almost the same number of CpGs at 15X coverage as in the WGBS.

Action taken: To address the latter, we re-sequenced the cell lines to a much greater depth, resulting in an average sequencing depth of 82X coverage between all cell lines and a set of 18 and 19 million CpGs covered by a minimum of 15 reads in TKO_L and DKO_0 cell lines, respectively. Next, we took a closer look at the Nanopore methylation precision; we used the WT ES cell line and added a **new panel (c) to Extended Data Figure 3**, showing the comparison of WGBS and Nanopore methylation rates in CpG resolution and 1kb windows. Briefly, while some divergence can be observed on single CpG resolution, only a 4.2% of these 1kb windows exceed a difference of 0.2, most of them (2.9%) are very conservative and underestimate the WGBS methylation level, indicating a good precision of 5mC detection.

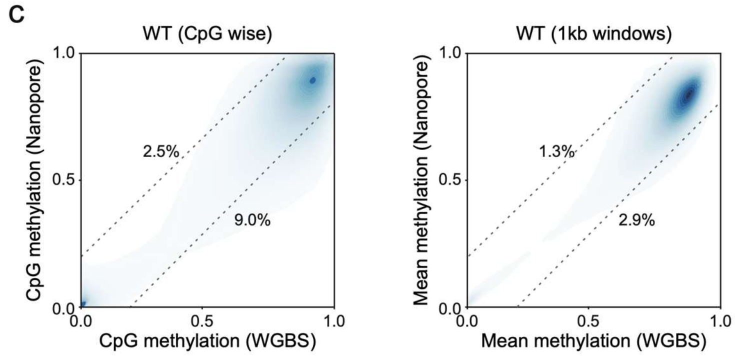

5. Fig 3a,b - Data doesn't indicate that DNMT1 protein was expressed in UHRF1 KO cells. There could be all sorts of reasons this might not happen. Also, could be strengthened by ectopic DNMT1 expression.

Response: The reviewer is absolutely right that we should have included this additional control.

Action taken: We now added an **updated panel a in Figure 4** (former figure 3), with a Western blot determining the presence of Dnmt1 in both the C5 and A10 Uhrf1 KO clones upon shDnmt1 excision. Additionally, we have added WGBS of Uhrf1 DKO_0 p15 for both A10 and C5 clones to **revised Figure 4**,

which has methylation levels comparable to the TKO_L both globally and at DMRs. Coverage statistics and average CpG methylation of the Uhrf1 KO DKO_0 p15 clones have been added to **revised Figures 4b-c.**

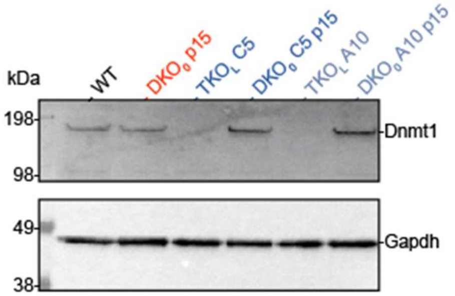

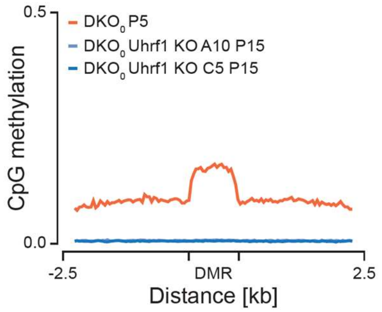

6. To generate the UHRF1-FLAG, I couldn't find the sequence of "Uhrf1_FLAG_sg", the suppl tables are not named as in text, not easy to navigate through.

Action taken: We have fixed this in the current version on page 7 as well as added the missing Uhrf1_FLAG_sg to the table (**Supplementary Table 1**).

7. To claim that Trim 28 might provide an interaction scaffold for UHRF1, the authors could KD Trim28 in DKO0 and test if remethylation happens, otherwise it's not conclusive.

Response: This is a good suggestion and aligns with some requests from reviewer 2. As such, we have considered various options such as knockdown, conditional knockdown/out, or degron lines. Due to the known lethality of Trim28 depletion, one would have to build conditional systems and the time it might take to derive clonal lines will likely be prohibitory. For instance, we could try to use a system that is the inverse of our shRNA against Dnmt1 and therefore would allow for conditional activation of shRNA expression upon Cre transfection (PMID: 15240889). However, this would mean we would need to first transfect Cre, then sort for the population that successfully recombine inside this limited timeframe. Additionally, problems with clones that exhibit weaker knockdown gaining a selective advantage would remain. Also, survival issues that arise due to sorting or selecting an already stressed population of cells would affect a conditional knockout system as well.

To circumvent these limitations, we designed and engineered a Trim28 degron cell line as outlined below. We had hoped that this would be enough to explore the role of Trim28 directly. Unfortunately, while the system turned out to work great (degradation of Trim 28 within hours, see Figure below) the dTag treatment results in severe lethality within days and that is not sufficient for collecting appropriate samples based on our data in **Figure 2**.

To nonetheless adjust to the reviewers points, we have revised the text on page 6 and provide the following additional considerations:

We would like to note that both Uhrf1 recognition of H3K9me3 and Dnmt1's interaction with the dual H3K18/23ub mark, which is catalyzed by Uhrf1, have been demonstrated in several instances across the literature (Dnmt1-H3K18/23: PMID: 29053958, PMID: 26065575, PMID: 32675241; Uhrf1-H3K9me3: PMID: 29395786, PMID: 23022729, PMID: 23752590). Additionally, Trim28's recruitment to transposable elements in the genome through KRAB zinc finger recognition and its recruitment of Setdb1, which catalyzes H3K9me3, are both well supported (PMID: 20075919, PMID: 11959841, PMID: 29728365). We provide H3K9me3, Trim28, and Uhrf1 ChIP-seq/ChIPmentation data sets for both TKO_L and DKO_0, which indicates that both the histone modification and the enzyme that catalyzes the ubiquitin are enriched at DMRs (no ChIP suitable antibodies are available for H3K18/23ub are available at this time). Further mass spec data validate our Uhrf1 mapping by pulling down Trim28 as well as heterochromatic factors and show Dnmt1 enrichment.

Action taken: As noted above we generated an inducible degradation system for Trim28 by inserting the Fkbp degron at the endogenous Trim28 locus through sgRNA/Cas9 targeting and homology-directed repair (PMID: 29581585). This overcomes several issues that were stated above, as the cell line would be clonal and the effect is mediated through the endogenous Trim28, no subpopulation effects would be present. Additionally, the use of a freely diffusing small molecule to initiate Trim28 protein degradation, would not necessitate sorting for effected cells. We started by cloning the sgRNA that targets the N-terminal region of Trim28 into px458, which expresses it from a U6 promoter and contains Cas9 under the control of a constitutively active CMV promoter. This was followed by assembling the homology arms that correspond to the 800bp of the genome on either side of the Trim28 sgRNA recognition site with the Fkbp-mCherry fusion sequence. After these were cloned TKO_L cells were transfected with both the sgRNA and Fkbp-mRuby repair template and FACS for mRuby2 signal after 3 days. The resulting GFP/mRuby2 double-positive cells were expanded at low density and single clones were picked. These were then screened for Fkbp-mRuby2 insertion by western blotting for Trim28 in order to visualize the expected size shift due to Fkbp-mRuby insertion. Several clones that had the correct size shift were expanded and then used for further functional tests.

To test if the system was effective at degrading Trim28 500nM dtag-13, the small molecule that links Fkbp to the E3 ligase responsible for ubiquitin deposition and proteasomal targeting, was added to several clones over a 24 hour time course and assayed by western blotting. One clone that showed near

complete degradation within 24 hours was then expanded and transfected with Cre for removal of the shRNA against Dnmt1. Another round of FACS for mRuby single positive cells followed this in order to generate DKO$_0$-Fkbp-mRuby2 cells. These cells were expanded and then subjected to culture in dtag-13 containing media, which unfortunately led to severe growth arrest and cell death in a few days. We therefore had to revert to revising the text and hope the restructured section still addresses the core concerns of the reviewer.

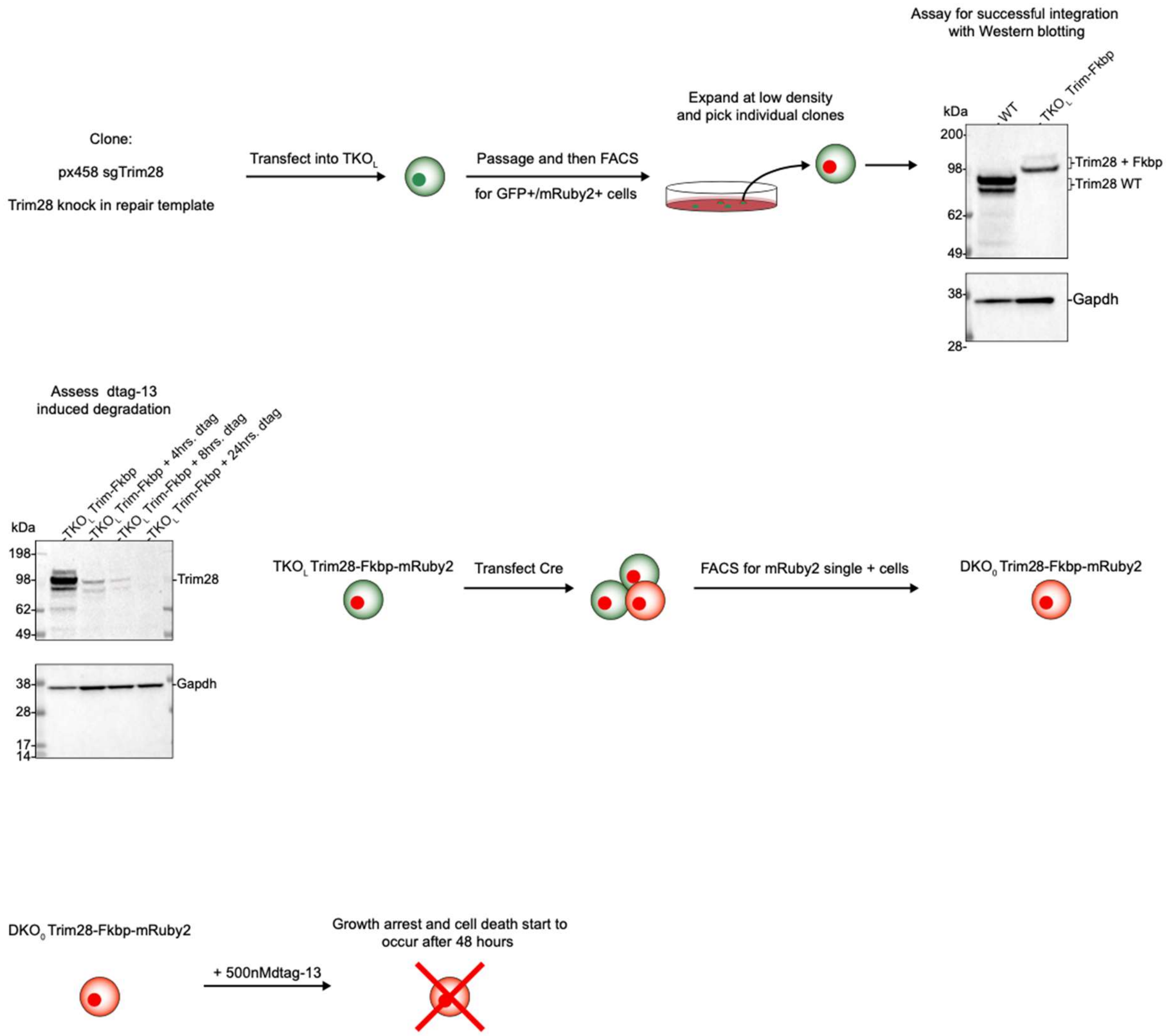

8. How can the authors explain the finding that the E6.5 recovery methylation level, which was modest, was able to suppress completely the expression of IAPEz in Fig 4f?

Response: The methylation values depicted in **new Fig. 6d, e** (former figure 4) are the change between E3.5 and E6.5. At E3.5 IAPs are already methylated higher than background levels (0.32) and the increase observed between these time points in the Dnmt3a/3b DKO + Dnmt1i samples results in average IAP methylation of (0.47) at E6.5. However, although the average methylation level in the DKO^{Dnmt1i} recovery doesn't reach that observed in WT, read-wise methylation analysis reveals a very low fraction of completely unmethylated reads that remains (see figure below and **Extended Data Figure 6f**). Therefore, the average methylation level is not indicative of the total amount of unmethylated cells (alleles) but a result of discordant (partial) methylation across the IAPEz-int. Due to the relatively high absolute number of CpGs, intermediate methylation levels are likely sufficient to repress IAPEz-int expression.

Action taken: We added the read-wise methylation figure to the new **Supplementary Figure 6f** and changed the main text on page 9 to emphasize the effect of discordant methylation. The figure shows the classification of all reads overlapping IAPEzs by their read-wise methylation level. While the WT loses almost all unmethylated reads (blue) from E3.5 to E6.5, a very similar trend can be observed in the DKO with Dnmti1 inhibitor recovery after recovery by E6.5. The reads gain at mainly hemi-methylation (green, yellow, orange), which is in contrast to the Dnmt1 KO, which loses hemi-methylated reads and gains unmethylated reads, in line with the loss of methylation at IAPEzs.

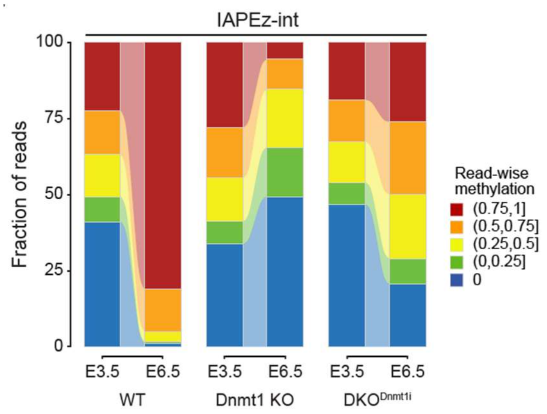

Minor points:

- not clear to a general reader what 0.05 means in this context "96% IAPs are less methylated, loss >0.05, in the Dnmt1 KO", a non-specialist reader might understand 5%, 0.05% or a p-value, just add more detail to be clearer. Same for "The DMRs have a mean methylation of 0.162"

Response: We agree with the reviewer, that the way methylation levels were stated could be improved.

Action taken: We added more details to the text on page 3, now reading: “96% of IAPs being less methylated in the E6.5 Dnmt1 KO versus E3.5 ICM (methylation loss defined as a difference greater than 5% (delta = 0.05))”.

- can the authors briefly explain why they used MeDIP (with all its inherent non-linear enrichment of DNA methylation) when they could have done WGBS? Especially since small methylation changes cannot be confidently called with MeDIP.

Response: We agree with the reviewer that the precise measurement of methylation changes cannot be generated using MeDIP. As the reviewer already stated, MeDIP is highly sensitive to any methylation and, although not quantitative, will give a reasonable binary estimate, e.g., presence or absence of methylation in regions.

By normalizing the number of reads sequenced within DMRs by the number of background reads, we cannot make any assumptions about absolute methylation levels – we fully agree with the reviewer. However, we only used the shift in the distribution across samples and passages to compare the focal enrichment of reads, to conclude that the same regions show focally higher enrichment across replicates and that the enrichment increased after the first passage.

Finally, to directly answer the reviewers’ question, the MeDIP experiments were among the earlier exploratory experiments. While we considered leaving it out for the reasons the reviewer states, we figured it does actually add some value and independent confirmation.

Action taken: To guide the reader better we have updated the text on page 4 to explain the rationale for including the method and resulting data.

- It’s not very clear here, is n=4,708 excluded or remaining? Same with 13Mb “First, we directly excluded all CpGs from the DMR detection that are located in regions detected as a deletion in the TKOL or DKO0 but present in the reference genome (n = 4,708 regions, in total 13Mb).”

Response: We apologize for the confusion and rewrote the sentence to be more precise. In addition, we sequenced the Nanopore samples to a greater depth, now having an average coverage of 82X. This allowed

us to improve the deletion (structural variation) calling and thereby increased the number of regions detected as a deletion in both, TKO_L and DKO_0.

Action taken: We clarified in this part of the manuscript on page 6 and it now reads: n = 8,210 missing regions, in total 26Mb, excluded from the analysis.

Reviewer #2:

Remarks to the Author:

In this study, Haggerty, Kretzmer et. al. provide a comprehensive dissection and analysis of Dnmt1 "de novo" methylation activity in early development. Through stringent genetic manipulations during early mouse development, Dnmt1 is demonstrated to have "de novo" methylation activity that is enriched at IAP retrotransposons. This finding is reinforced through the novel use of long-read Nanopore sequencing to ensure the Dnmt1 dependent methylation at repetitive elements by significantly increasing the coverage of uniquely mapped reads at repetitive regions of the genome. The authors additionally demonstrate that Dnmt1 "de novo" activity is reliant on the presence of the cofactor Uhrf1. Using mass spectrometry, it is demonstrated that Uhrf1 interacts with the heterochromatic scaffolding protein, Trim28, and the authors hypothesize that this interaction coupled with association of H3K9me3 helps direct Uhrf1, and subsequently Dnmt1, to IAP retrotransposons for "de novo" methylation deposition. Finally, using in vivo mouse models, the authors demonstrate that "de novo" methylation activity of Dnmt1 is required to effectively repress IAPz-gag expression.

Overall, the study provides rigorous evaluation of Dnmt1 methylation activity in early development which is currently an active area of research. The in vitro, in vivo, and extensive genome-wide DNA methylation sequencing provide strong evidence for the role of Dnmt1 in silencing IAP retrotransposons during development by DNA methylation.

We very much appreciate the reviewers thorough reading of the manuscript and constructive feedback that we hopefully addressed sufficiently, as outlined below.

However, a few aspects of the conclusions need to be addressed before the study is suitable for publication at NSMB:

Major Comments:

- The genetic experiments in Figure 1 and Figure 2 strongly suggest that Dnmt1 plays a major role in suppressing IAP retrotransposons in early development by DNA methylation. However, the ability to call this methylation "de novo" activity by Dnmt1 requires further evidence. For example, in Figure 1j, while DNA methylation is very low in the TKOL cells, there does appear to be a small amount of DNA methylation remaining, particularly at regions that are later deemed "de novo" methylated by Dnmt1 in later passages. Potentially, the small amount of DNA methylation that remains in a subpopulation of cells may serve as a template for Dnmt1 to perform traditional "maintenance" methylation (e.g., activity toward hemi-methylated DNA substrates), and the increase in DNA methylation at the later passages is a result of outgrowth of this subpopulation. To firmly establish that this gain in methylation is due to "de novo" activity of Dnmt1, performing strand-specific analyses like hairpin bisulfite sequencing would help demonstrate that Dnmt1 is acting in a de novo manner (e.g., methylating a fully unmethylated DNA substrate) at the identified genomic regions.

Response: We thank the reviewer for this constructive feedback and agree with the reviewer that hairpin bisulfite would be a very good method to quantify the gain of hemi-methylated sites as an additional measurement of *de novo* methylation contrasting the maintenance activity. Additionally, we included an experiment to determine if a subpopulation of cells retain methylation, which is outlined below, to better control for subpopulation-specific effects.

Action taken: To ensure high quality hairpin bisulfite data, we teamed up with the lab of Jörn Walter, who has used this approach successfully before including on repetitive elements (see page 5). We performed the hairpin bisulfite sequencing on WT, TKO_L, P1, and P5 DKO_0 cells across IAPEz, IAP-LTR, L1, major satellite, and MERVL elements. Both full and hemimethylation increases in the DKO_0 by P5 in all classes but major satellites, which only shows an increase in fully methylated sequences. Furthermore, IAPEz's show a relative increase compared to TKO_L in hemimethylted CpG dyads to 75% of WT levels by P5 (new **Fig. 2h**), which is notable considering that *de novo* Dnmt3s are active in the WT. Additionally, we used this hairpin data as input for a Hidden Markov Model to estimate *de novo* and maintenance methylation efficiency. For most repeat classes, the low levels of methylation in the KO lines make modeling difficult; however, the increased methylation observed at IAPEz-int elements allowed us to effectively generate a model of *de novo* versus maintenance methylation (new **Fig. 2i**). The model predicts a *de novo* methylation catalyzed by Dnmt1 that aligns well with the gain of methylation observed in the WGBS passaging data.

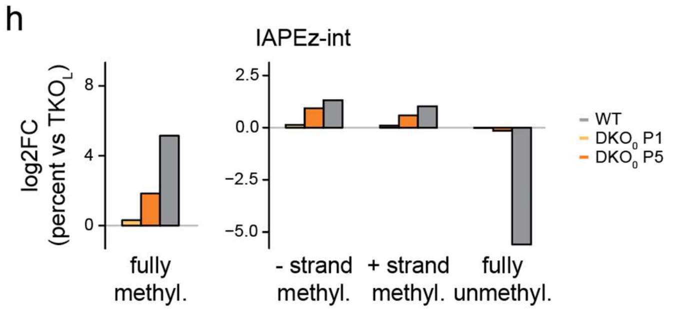

To further rule out that a subpopulation of cells retain methylation at DMRs we sorted (FACS) single cells from a DKO_0 P1 population and passaged them until P5 and then assayed methylation at IAPEz elements with amplicon bisulfite sequencing (see page 4, new **Extended Data Fig. 2a**). If a subpopulation of cells in the TKO_L retained methylation and this is leading to the gain, we would expect a bimodal landscape in the clones; either elevated methylation at DMRs or a complete lack of such. In contrast, the amplicons reveal that methylation varies little between the nine clones assayed and a similar pattern emerges. This argues against a residual subpopulation as the explanation for our observed methylation. We would like to further point out that the methylation gain is specific to IAP's with other classes of transposable elements that are upregulated upon loss of DNA methylation remaining less targeted. This would be unexpected if a subpopulation gained a selective advantage from TE silencing, unless IAPs were the sole class of transposable element that has a deleterious effect.

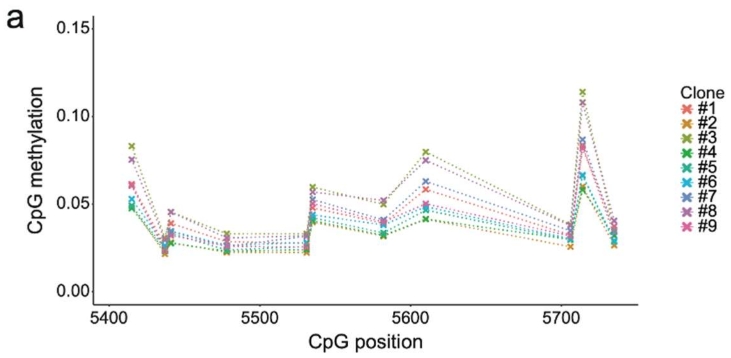

- While the results support the hypothesis that Uhrf1 is needed for Dnmt1 activity at these regions, the rationale for the mechanism by which Uhrf1 and Dnmt1 are recruited is speculative at best. The authors hypothesize that since Trim28 interacts with Uhrf1, Trim28 aids in the deposition of H3K9me3 providing a means of recruitment for Uhrf1 through its histone binding domains. However, recent work suggests that H3K9me3 is dispensable for Uhrf1 role in DNA methylation maintenance (PMID: 27554592; PMID: 33097091; PMID: 30956060). The authors strongly hint at this mechanism by stating there is "co-

localization” and “association” with H3K9me3 and Trim28; however, there is no functional evidence that demonstrates the requirement of Trim28 and H3K9me3 for Uhrf1 recruitment at these genomic locations.

Response: We thank the reviewer for pointing this out. We restructured the section dealing with the recruitment mechanism since we believe the data could be presented in a more straightforward fashion. To this end, we would like to note that both Uhrf1 recognition of H3K9me3 and Dnmt1’s interaction with the dual H3K18/23ub mark, which is catalyzed by Uhrf1, are demonstrated in several instances across the literature (Dnmt1-H3K18/23: PMID: 29053958, PMID: 26065575, PMID: 32675241; Uhrf1-H3K9me3: PMID: 29395786, PMID: 23022729, PMID: 23752590). Additionally, Trim28’s recruitment to transposable elements in the genome through KRAB zinc finger recognition and its recruitment of Setdb1, which catalyzes H3K9me3, are both well supported (PMID: 20075919, PMID: 11959841, PMID: 29728365). We provide H3K9me3, Trim28, and Uhrf1 ChIP-seq/ChIPmentation data sets for both TKO_L and DKO_0, which indicates that both the histone modification and the enzyme that catalyzes the ubiquitin mark are enriched at DMRs (no ChIP suitable antibodies are available for H3K18/23ub are available at this time). Further mass spec data validate our Uhrf1 mapping by pulling down with heterochromatic factors and show Dnmt1 enrichment.

We do agree that the vast majority of replication coupled methylation is largely independent of H3K9me2/3 and presumably linked to recruitment of Uhrf1 and Dnmt1 to the replication fork during S-phase (PMID: 32144273, PMID: 28803780). However, as noted in PMID: 27554592, which was cited by reviewer #2, in cells which Uhrf1 is insensitive to H3K9me2/3 a roughly 10% reduction in global DNA methylation is observed with LTRs losing 6.78%. We would like to note that the global loss of 10% is almost double the increase that we observe globally in the DKO_0 at P15 (5.6%), which closely matches what is observed at LTRs in that publication. The paper also states that this loss happens globally but demonstrates a range across repeat elements from less than 1% to an almost 9% loss, indicating that this might not be homogenous across the genome or masked by Uhrf1’s SRA domain recognizing methylated DNA. As the paper states “while our study supports a role for H3K9 methylation in promoting DNA methylation, it demonstrates for the first time that DNA maintenance methylation in mammals is *largely* independent of H3K9 methylation.”

Action taken: Please see the response to Reviewer 1 comment 7.

Based on all this, we have updated the text accordingly and hope that with our experimental data together with existing literature, we present a sufficiently convincing case for the possible underlying mechanism.

Minor comments:

- The mass spectrometry results in Figure 3f seem to be selective of interactions that support the hypothesis; however, there appear to be several other protein interactions with Uhrf1 that are not discussed.

Response: We would like to note that the whole panel showing all overrepresented proteins is present as **Extended Data Figure 5c**, due to the rather complex and busy nature of the panels. However, we agree that the text should also address the enrichment of other proteins and make it clearer that Uhrf1 is known to interact with other protein complexes that are involved in processes such as DNA replication or DNA damage response (PMID: 28803780, PMID: 30357346, PMID: 25818288). To highlight this, we utilized GO Term analysis of the significantly enriched proteins found in the DKO_0 or TKO_L Uhrf1-FLAG IPs, which is included as new **Extended Data Fig. 5d**.

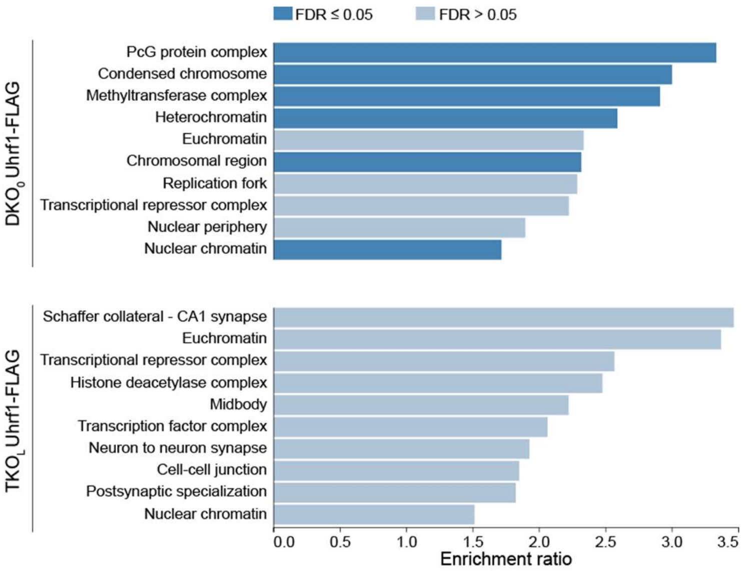

Action taken: Clarified this in text on page 7: "Additionally, GO term analysis of enriched proteins highlights terms associating with heterochromatin and the replication fork, another site of Uhrf1 recruitment, were enriched". Added new **Extended Data Fig. 5d**

- It is an overstatement to say that the enrichment of H3K9me3 in DMRs of interest indicates that it has an impact on DNMT1 activity as stated in the text.

Action taken: We have adjusted the text on page 6 as follows: "Moreover, H3K9me3 peaks tend to be higher methylated than other regions suggesting that H3K9me3 may influence Dnmt1's recruitment or activity at DMR regions (Extended Data Fig. 4f)."

- The physiologic/pathologic relevance of this finding is unclear.

Response: We agree that it is not entirely clear whether this has evolved as a functional role for DNMT1 because it was needed, for instance, as an additional safety mechanism or whether this is just a mechanistically explainable activity based on the involved modifications and other factors. That said, we find the observation together with a few other recent publications of notable interest to the field and hope to advance our understanding of the still evolving field of DNA methylation and DNA methyltransferases.

Decision Letter, first revision:

13th Apr 2021

Dear Alex,

Thank you again for submitting the revision of your manuscript "Dnmt1 has de novo activity targeted to transposable elements". We have now received comments from the two original referees, who assessed the revision (appended below). You will see that both reviewers appreciate the addition of the hairpin bisulfite data, but there are some questions in regards to these data and the associated analysis that will require clarification. In addition, reviewer #2 finds that clonal analysis of TKOL cells is essential. In light of these remarks, we remain interested in your study, but would like to see your response to these points, in the form of a revised manuscript. Please be sure to address/respond to all concerns of the referees in full in a point-by-point response and highlight all changes in the revised manuscript text file. If you have comments that are intended for editors only, please include those in a separate cover letter.

We are committed to providing a fair and constructive peer-review process. Do not hesitate to contact us if there are specific requests from the reviewers that you believe are technically impossible or unlikely to yield a meaningful outcome.

We expect to see your revised manuscript within 4-6 weeks. If you cannot send it within this time, please contact us to discuss an extension; we would still consider your revision, provided that no similar work has been accepted for publication at NSMB or published elsewhere.

As you already know, we put great emphasis on ensuring that the methods and statistics reported in our papers are correct and accurate. As such, if there are any changes that should be reported, please submit an updated version of the Reporting Summary along with your revision.

Please follow the links below to download these files:

Reporting Summary:
https://www.nature.com/documents/nr-reporting-summary.pdf

Please note that the form is a dynamic 'smart pdf' and must therefore be downloaded and completed in Adobe Reader.

When submitting the revised version of your manuscript, please pay close attention to our href="https://www.nature.com/nature-research/editorial-policies/image-integrity">Digital Image Integrity Guidelines. and to the following points below:

-- that unprocessed scans are clearly labelled and match the gels and western blots presented in figures.
-- that control panels for gels and western blots are appropriately described as loading on sample processing controls
-- all images in the paper are checked for duplication of panels and for splicing of gel lanes.

Finally, please ensure that you retain unprocessed data and metadata files after publication, ideally archiving data in perpetuity, as these may be requested during the peer review and production process or after publication if any issues arise.

Please note that all key data shown in the main figures as cropped gels or blots should be presented in uncropped form, with molecular weight markers. These data can be aggregated into a single supplementary figure item. While these data can be displayed in a relatively informal style, they must refer back to the relevant figures. These data should be submitted with the final revision, as source data, prior to acceptance, but you may want to start putting it together at this point.

SOURCE DATA: we urge authors to provide, in tabular form, the data underlying the graphical representations used in figures. This is to further increase transparency in data reporting, as detailed in this editorial (http://www.nature.com/nsmb/journal/v22/n10/full/nsmb.3110.html). Spreadsheets can be submitted in excel format. Only one (1) file per figure is permitted; thus, for multi-paneled figures, the source data for each panel should be clearly labeled in the Excel file; alternately the data can be provided as multiple, clearly labeled sheets in an Excel file. When submitting files, the title field should indicate which figure the source data pertains to. We encourage our authors to provide source data at the revision stage, so that they are part of the peer-review process.

Data availability: this journal strongly supports public availability of data. All data used in accepted papers should be available via a public data repository, or alternatively, as Supplementary Information. If data can only be shared on request, please explain why in your Data Availability Statement, and also in the correspondence with your editor. Please note that for some data types, deposition in a public repository is mandatory - more information on our data deposition policies and available repositories can be found below:
https://www.nature.com/nature-research/editorial-policies/reporting-standards#availability-of-data

Deposition of deep sequencing data is mandatory, and the datasets must be released prior to or upon publication. To avoid delays in publication, dataset accession numbers must be supplied with the final

accepted manuscript and appropriate release dates must be indicated at the galley proof stage.

While we encourage the use of color in preparing figures, please note that this will incur a charge to partially defray the cost of printing. Information about color charges can be found at http://www.nature.com/nsmb/authors/submit/index.html#costs

Please use the link below to submit your revised manuscript and related files:

[REDACTED]

Note: This URL links to your confidential home page and associated information about manuscripts you may have submitted, or that you are reviewing for us. If you wish to forward this email to co-authors, please delete the link to your homepage.

We look forward to seeing the revised manuscript and thank you for the opportunity to review your work.

With kind regards,
Anke

Anke Sparmann, PhD
Senior Editor
Nature Structural and Molecular Biology
ORCID 0000-0001-7695-2049

Reviewers' Comments:

Reviewer #1:
Remarks to the Author:
The evidence for DNMT1 de novo activity provided is rather convincing, and this is further backed up by the addition of the hairpin bisulfite data. Regarding the mechanism, the authors tried various avenues to confirm that TRIM28 acts as a scaffold to mediate de novo-methylation by DNMT1 but due to experimental challenges this was not possible. The authors have formulated the argument carefully in the absence of the conclusive data. This is a valuable study for the research community interested in the nuances of DNA methyltransferases, in particular for DNMT1. The authors have addressed all of my other concerns.

One question on the new data in (Fig ED3a and b): can the authors speculate why is it that the IAP LTRs are much slower in maintaining DNA methylation on the “newly” hemimethylated strands from DKO0-P1 to DKO0-P5 compared to IAPEz-int? The data gives the impression that DNMT1 has a bias towards de-novo activity and loses its maintenance activity? This seems to be an interesting data pointing again towards locus/sequence specific molecular ecosystems in epigenetic mechanisms.

Reviewer #2:
Remarks to the Author:
In this resubmission, Haggerty et al performed additional experiments and analysis, as requested, to further support their conclusion that Dnmt1 functions as a de novo methyltransferase at retrotransposons in early development. However, major concerns from the prior review are not fully addressed in this resubmission, and further analysis/discussion related to Major Comments below are needed to more strongly support key conclusions of this study.

Major Comments:

1) The addition of hairpin bisulfite sequencing data significantly adds to the dissection of Dnmt1 maintenance versus de novo activity. However, aspects of this analysis and interpretation of the results need further consideration. For example, in Figure ED3a, the authors use results from IAPEz-int to support their conclusion that Dnmt1 acts in a de novo manner at these loci. However, the data in the TKO¬¬L demonstrates that 2.54% of the CpGs are methylated on both strands and that, cumulatively between both stands, 3.29% of the CpGs are hemimethylated BEFORE Dnmt1 is reintroduced to the system. The authors state, "We observed an increase in fully methylated CpG dyads across all repeat classes relative to the TKOL by P5, with IAPEz's showing the highest fraction. Additionally, in contrast to other repeat classes, IAPEz's show a relative increase in hemimethylated CpG dyads on both strands across passaging and notably reach 75% of WT by P5 (compare to TKOL)." Indeed, the authors consistently demonstrate the increase in both fully methylated CpGs and hemimethylated CpGs in the DKO0 cells in the analysis presented in ED3b and Figure 2h-i relative to the TKOL cells as a Log2FC.

Another concern with the hairpin bisulfite sequencing analysis and interpretation is that there is no acknowledgement that the TKOL cells already contain both hemimethylated and fully methylated CpGs, providing a template for Dnmt1 to perform maintenance methylation. Indeed, presentation of the data as Log2FC relative to TKOL cells masks that TKOL cells do contain CpGs that are methylated and can serve as a template for Dnmt1 maintenance methylation. Analysis of this data should include a separation of CpGs that are already methylated in TKOL and maintained in the DKO0 passages versus truly de novo methylated CpGs that were completely unmethylated in TKOL and gained methylation after reintroduction of Dnmt1 in the DKO0 passages. The hairpin bisulfite sequencing data confirms that there is indeed de novo methylation; however, it also demonstrates that maintenance methylation is occurring. The fact that both maintenance and de novo methylation is occurring in this system needs to be further addressed in the manuscript and additionally needs to be discussed in terms of how to account for both mechanisms occurring in the WGBS data.

2) To address the concern of an outgrowth of a subpopulation from TKOL cells that retained methylation, the authors conducted MeDIP-seq and amplicon bisulfite sequencing on a number of clones derived from DKO0 P1 cells that were passaged an additional four times (P5). While the data certainly shows reproducibility of methylated DMRs across all clones, it still does not address whether methylation was already present in the TKOL cell population. Clonal analysis of TKOL cells is needed to confirm that these are truly de novo hypermethylated DMRs.

3) In the rebuttal, the authors state, "If a subpopulation of cells in the TKOL retained methylation and

this is leading to the gain, we would expect a biomodal landscape in the clones; either elevated methylation at DMRs or a complete lack of such." This rationale is unclear without conducting the same analysis in TKOL cells. How would you interpret elevated methylation in the DKO0 P5 cells?

4) The additional analysis of Uhrf1 mass spectrometry data in Figure ED5c-d sufficiently addresses the original minor comment. The authors also provided sufficient attempts (degron of Trim28) and rationale for the inability to demonstrate the dependence of the proposed mechanism on Trim28. The language in the text appropriately does not overstate the interpretation of the presented data; however, the authors still heavily suggest this mechanism could be at play in both the results and discussion sections without real evidence outside of overlaps of distributions and interactions from mass spec. Notably, it does appear the distribution of H3K9me3 (Figure ED4d), Uhrf1 (Figure ED5b), and Trim28 (Figure ED5e) do not change in the different cell populations, and this is not commented on in the text.

Minor Comments:

1. Why is the color scale range for Uhrf1 binding different from Figure 5C (range = [0-2]) versus Figure ED5b (range = [0-0.25])? The same is true for Trim28, Figure 5c versus Figure ED5e.

2. Check the data in Figure ED3g --- the color scheme is swapped from Figure ED3d, and the data in the current form demonstrates that methylation in TKOL at both the remaining DMRs (from Nanopore analysis) and excluded DMRs is elevated compared to DKO0 --- this is in contrast to the rest of the data presented in the manuscript. Indeed, the data does not match the interpretation that is written in the figure legend: "Both subsets show comparable methylation levels when measured by WGBS, indicating that they don't represent a low or high methylated outlier set." The data does not support this statement as currently presented. Additionally, the y-axis is labeled "Men methylation" instead of "Mean methylation".

3. Color scale in Figure 6d differs from how Mean methylation is presented elsewhere in the manuscript (e.g. Figure 5c). Indeed, the blue coloring appears to be below 0.0, and given the range of methylation data [0-1], the blue color should not appear at all in the heatmaps.

4. In Figure 3d, the data for TKOL (the control) also needs to be presented in this way in the figure to show the change in methylation at DKO0 P15 at individual loci. Potentially (and as indicat¬¬ed by the hairpin bisulfite sequencing data), TKOL does have loci that are methylated, and these loci are masked/diluted in the average profiles presented in Figure 3c.

Author Rebuttal, first revision:

Reviewers' Comments:

Reviewer #1:
Remarks to the Author:
The evidence for DNMT1 de novo activity provided is rather convincing, and this is further backed up by the addition of the hairpin bisulfite data. Regarding the mechanism, the authors tried various avenues to confirm that TRIM28 acts as a scaffold to mediate de novo-methylation by DNMT1 but due to experimental challenges this was not possible. The authors have formulated the argument carefully in the

absence of the conclusive data. This is a valuable study for the research community interested in the nuances of DNA methyltransferases, in particular for DNMT1. The authors have addressed all of my other concerns.

We appreciate the summary and are glad that we could satisfy all the concerns raised. Below we provide a short reply to the final question.

One question on the new data in (Fig ED3a and b): can the authors speculate why is it that the IAP LTRs are much slower in maintaining DNA methylation on the "newly" hemimethylated strands from DKO0-P1 to DKO0-P5 compared to IAPEz-int? The data gives the impression that DNMT1 has a bias towards de-novo activity and loses its maintenance activity? This seems to be an interesting data pointing again towards locus/sequence specific molecular ecosystems in epigenetic mechanisms.

If we understand the question correctly, the reviewer is curious about the following part (purple boxes, lack of orange 5mC I 5mC pairs in IAP LTR and limited gain of hemi-methylated (green) strands) in

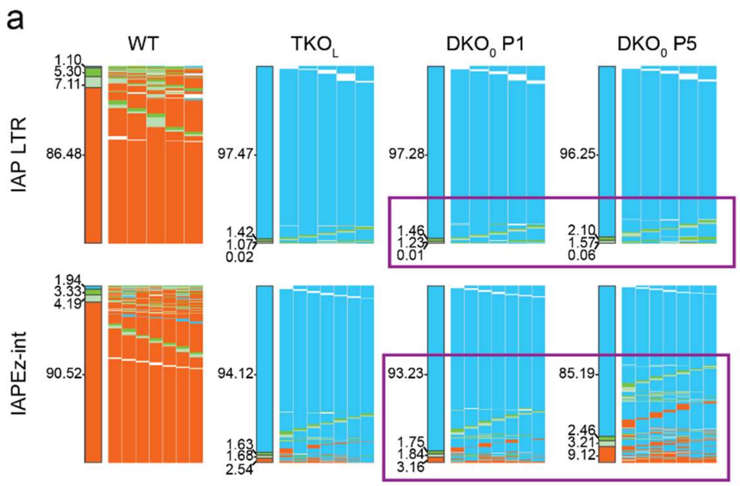

Extended Data Figure 3a):

While indeed intriguing, one first needs to take important technical aspects into consideration. In particular, due to the low frequency of methylation observed in the IAP LTR hairpin samples, which is close to the bisulfite conversion error range, it is in our opinion difficult to make a conclusive/strong biological statement.

That said, the difference is in line with the strong enrichment of DMRs in IAPEz-int elements, but not LTRs, in our WGBS data (see Figure 2e and f, Extended Data Figure 2c).

Assuming the low signal in the hairpin data is not noise, several additional biological factors could indeed play a role in the observed divergent methylation profile:

1) Most notably, the distinct genomic distribution and frequency of IAP LTRs (n = 10,677 including 4,522 solo LTRs) and IAPEz-int elements (full length, n = 2,656) and the specific targeting of IAPEz-int (Extended Data Figure 2c) would be a reasonable explanation.
2) The GAG sequence for the IAPEz-int region generally presents with the highest levels of methylation in our DKO_0 data and it is possible that some sequence motif within the IAPEz-int plays a role in Dnmt1 recruitment to these regions and constrains methylation gain to these elements. As LTRs do not have this, they might not be favored targets of Dnmt1's *de novo* activity.
3) The large pool of potentially lower targeted solo LTR elements and IAPEz-int sequence specific targeting could then explain these observations, which reviewer 1 recognizes could be interesting.

To ensure this is more obvious to the reader, but also not speculate too much, we would propose adding the following sentence to page 5 of the manuscript:

"This increase in hemimethylated DNA, specifically at full length IAPEz elements over other repeat classes, including the larger set of IAP LTRs that includes more than 4,000 solo LTRs, could potentially be the result of sequence specific features that influence Dnmt1's recruitment and hence its *de novo* activity."

Reviewer #2:
Remarks to the Author:
In this resubmission, Haggerty et al performed additional experiments and analysis, as requested, to further support their conclusion that Dnmt1 functions as a de novo methyltransferase at retrotransposons in early development. However, major concerns from the prior review are not fully addressed in this resubmission, and further analysis/discussion related to Major Comments below are needed to more strongly support key conclusions of this study.

We appreciate the reviewer's additional feedback and would like to note that these are not major issues and rather just need some simple clarification from our end. Please see below.

Major Comments:

1) The addition of hairpin bisulfite sequencing data significantly adds to the dissection of Dnmt1 maintenance versus de novo activity. However, aspects of this analysis and interpretation of the results need further consideration. For example, in Figure ED3a, the authors use results from IAPEz-int to support their conclusion that Dnmt1 acts in a de novo manner at these loci. However, the data in the

TKOL demonstrates that 2.54% of the CpGs are methylated on both strands and that, cumulatively between both stands, 3.31% of the CpGs are hemimethylated BEFORE Dnmt1 is reintroduced to the system.

For your convenience we have added the relevant part of Extended Data Figure 3a here (purple box) and yes the reviewer is correct about these numbers (we just fixed a typo in the reviewer comment 3.31% (1.63% + 1.68%) instead of 3.29%).

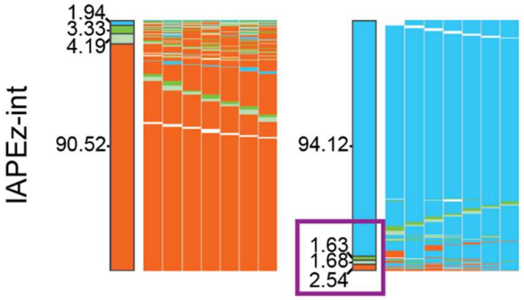

We will respond step by step to the points to make it most intuitive to follow.

The authors state, "We observed an increase in fully methylated CpG dyads across all repeat classes relative to the TKOL by P5, with IAPEz's showing the highest fraction. Additionally, in contrast to other repeat classes, IAPEz's show a relative increase in hemimethylated CpG dyads on both strands across passaging and notably reach 75% of WT by P5 (compare to TKOL)."

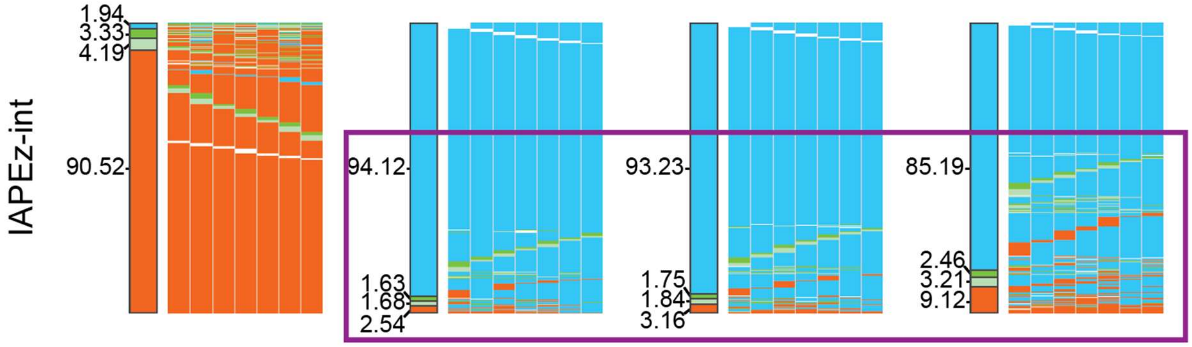

Again, to facilitate this, we have added the relevant part here (purple box and compare those to the two green parts in wildtype: 3.33%+4.19%=7.52% hemimethylated in wildtype). The DKO P5 reaches 2.46%+3.21%=5.67% (5.67/7.52=75%), which is indeed notable and clearly more than present in the TKOL

Indeed, the authors consistently demonstrate the increase in both fully methylated CpGs and hemimethylated CpGs in the DKO0 cells in the analysis presented in ED3b and Figure 2h-i relative to the TKOL cells as a Log2FC.

It is not entirely clear to us, what the concern is here. As shown above everything aligns well and is consistent with our presentation.

Based on the questions below, we speculate that the reviewer is possibly worried about the residual methylation in TKO_L cells, which we of course present very precisely throughout the manuscript. As expected, (by design) these cells can indeed still have some low level, residual Dnmt1 activity (maintenance and *de novo*).

There are a few brief points we would like to mention here that might help:

1) It is also correct, as the reviewer pointed out, that the amount of hemi-methylated sites does not change with the same rate in all classes profiled. In our opinion this argues against a pure cell division driven effect by maintaining strand-specific methylation to both strands and is therefore in line with our conclusions.

2) Even if the hemimethylated reads, which total 3.31% in the TKO_L, were converted perfectly into fully methylated reads by Dnmt1 maintenance this would still be 3.27% less than just the fully methylated fraction in the DKO_0 P5 (1.63%+1.68% (hemi, green) +2.54% (fully, orange) =5.85% versus 9.12% (fully, orange) found in DKO_0 P5).

3) In addition, when comparing the number of IAPEz-int elements that gain methylation with the very small fraction of remaining methylation, it is extremely unlikely that each element has some remaining methylation, which then leads to increased methylation by P5.

4) Finally, the comparison to TKO_L (which is not at zero methylation) seems to be appropriate. The Log2FC is standard and only done to facilitate this comparison (see also the more detailed point for Log2FC below).

Another concern with the hairpin bisulfite sequencing analysis and interpretation is that there is no acknowledgement that the TKOL cells already contain both hemimethylated and fully methylated CpGs, providing a template for Dnmt1 to perform maintenance methylation.

Please see the points above. The apparent lack of acknowledgement is not the case in our opinion. The data are very clearly presented (see again the purple box, same panel as shown above in a larger version) and it is quite obvious what fraction of hemi- or fully methylated strands are already present in the TKO_L and how they evolve in the DKO_0. All comparisons and interpretations take that into account.

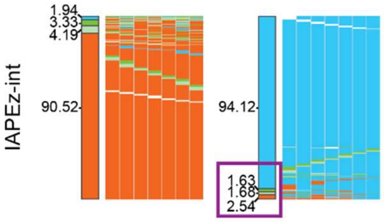

Indeed, presentation of the data as Log2FC relative to TKOL cells masks that TKOL cells do contain CpGs that are methylated and can serve as a template for Dnmt1 maintenance methylation.

We disagree here that this is masking anything. The Log2FC in Extended Data Figure 3b is used right next to the raw percentage data in Extended Data Figure 3a. So the reader cannot possibly miss that part and we utilize the different display of the data to help the reader appreciate the results.

The presentation in ED3a provides the detailed view including the numbers that we also highlighted above. The Log2FC panel in ED3b is utilized to better highlight the respective dynamic across the classes and between strands. The same information is in ED3a but it is just more cumbersome to compare across so many data points, hence the different way of displaying the data. This is very standard and rather than masking provides the reader with different angles to look at the same data.

Analysis of this data should include a separation of CpGs that are already methylated in TKOL and maintained in the DKO0 passages versus truly de novo methylated CpGs that were completely unmethylated in TKOL and gained methylation after reintroduction of Dnmt1 in the DKO0 passages.

This is a reasonable analysis that we have now done and could include on page 4/5 as well as ED Figure 2a (right). Briefly, to better highlight that we do not only observe a gain in methylation at CpGs that were already methylated in the TKO_L cells, we compared the gain in methylation between unmethylated and all other CpG in the DMR regions (n = 63,852 unmethylated, n = 12,531 with residual methylation). Of the unmethylated CpGs in the TKO_L 94% gain methylation by P15 DKO_0 (median delta = 0.139). These numbers are similar to the 95% of CpGs that already had minimal levels of methylation and gained methylation in the TKO_0 (median delta = 0.175).

Therefore, we would conclude, that unmethylated and residually methylated CpG in the TKO_L gain methylation at similar rates and yet again confirms clear *de novo* activity.

Comparison of TKO_L methylation levels and gain of methylation by P15 in the DKO_0 per CpG. Completely unmethylated CpGs (black) gain methylation to very similar levels as CpGs observed with residual methylation.

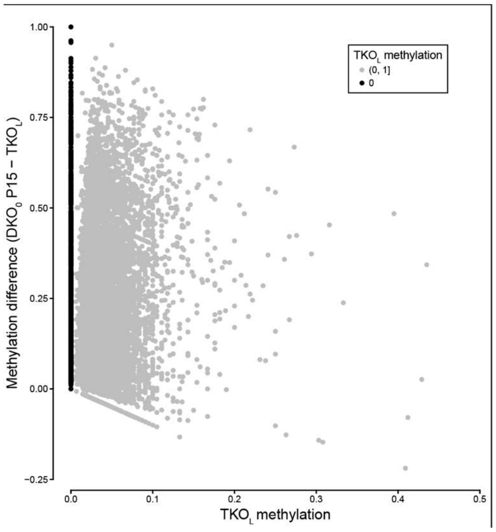

The hairpin bisulfite sequencing data confirms that there is indeed de novo methylation; however, it also demonstrates that maintenance methylation is occurring. The fact that both maintenance and de novo methylation is occurring in this system needs to be further addressed in the manuscript and additionally needs to be discussed in terms of how to account for both mechanisms occurring in the WGBS data.

We are a bit confused about this question. As expected Dnmt1 does indeed perform maintenance methylation, which can be seen in various parts and is specifically highlighted in **Figure 2i**. As the reviewer states here, we demonstrate the *de novo* activity and assume nobody (including us) questions the ability of Dnmt1 to perform maintenance activity based on our *de novo* data.

The HMM model in **Figure 2i** highlights the relative activities and makes it clear that there is our reported low-level *de novo* methylation activity and the expected much higher fidelity of maintenance methylation.

Of course, it is not possible to track individual molecules over cell division to distinguish *de novo* and maintenance activity at specific sites and hence have to infer this from the bulk analysis.

2) To address the concern of an outgrowth of a subpopulation from TKOL cells that retained methylation, the authors conducted MeDIP-seq and amplicon bisulfite sequencing on a number of clones derived from DKO0 P1 cells that were passaged an additional four times (P5). While the data certainly shows reproducibility of methylated DMRs across all clones, it still does not address whether methylation was already present in the TKOL cell population. Clonal analysis of TKOL cells is needed to confirm that these are truly de novo hypermethylated DMRs.

Here we just need to clarify that the TKO_L is already a subclone derived from a single cell. This is an essential aspect of the study, since, as the reviewer pointed out, a bulk TKO_L population could contain distinct subclones, which could maintain residual methylation and then expand upon reactivation of Dnmt1.

The fact that our starting line is already clonal implies that any remaining methylation found, was either already present in the picked single TKO_L cell or was *de novo* added during TKO_L cell line expansion. Based on that, we do not see any reasonable way to explain the data with a pure maintenance activity that acts in our clonal line.

Furthermore, the numbers that we discussed above acknowledge the residual levels, but given the further increase also do not allow any other interpretation than *de novo* activity. In addition, all the other data we provide in the manuscript, including the ectopic rescue in the complete (true) TKO cells, further support that.

Importantly, based on the statements also none of the reviewers questions the fact that there is *de novo* activity.

To address this specific concern, we propose the following additions to the text on page 4 to make the clonal nature of the experiment clearer:

"Additionally, the clonal nature of this line rules out that a subpopulation of cells retained methylation and then later expanded to give rise to any observed change in methylation. "

3) In the rebuttal, the authors state, "If a subpopulation of cells in the TKOL retained methylation and this is leading to the gain, we would expect a biomodal landscape in the clones; either elevated methylation at

DMRs or a complete lack of such." This rationale is unclear without conducting the same analysis in TKOL cells. How would you interpret elevated methylation in the DKO0 P5 cells?

We hope, that the clonal nature of our starting line helps explain why the observed methylation patterns in our clonal line cannot be explained by a subpopulation of TKO_L cells.

The bimodal distribution mentioned before refers to the expected global methylation profiles obtained from a population of cells after single cell sorting. Given that the majority of the genome gains methylation, the starting cell of a subpopulation would have to be methylated at almost all positions. Also, since the TKO_L WGBS shows a global level of 0.3% the majority of cells must be demethylated at almost all positions. Therefore, if there is a subpopulation and Dnmt1 knockdown is reversed, the resulting methylation profile would be either a highly methylated cell that gives rise to the subpopulation or a lowly methylated cell. Multiple cells presenting with higher methylation than the TKO_L WGBS data that matches the distribution of the DKO_0 should be impossible to achieve if the subpopulation hypothesis was true.

4) The additional analysis of Uhrf1 mass spectrometry data in Figure ED5c-d sufficiently addresses the original minor comment. The authors also provided sufficient attempts (degron of Trim28) and rationale for the inability to demonstrate the dependence of the proposed mechanism on Trim28. The language in the text appropriately does not overstate the interpretation of the presented data; however, the authors still heavily suggest this mechanism could be at play in both the results and discussion sections without real evidence outside of overlaps of distributions and interactions from mass spec. Notably, it does appear the distribution of H3K9me3 (Figure ED4d), Uhrf1 (Figure ED5b), and Trim28 (Figure ED5e) do not change in the different cell populations, and this is not commented on in the text.

We are glad to hear the reviewer is satisfied with the effort and changes regarding Trim28.

To address the remaining point, we propose the following change of the text on page 8 (Results): "While speculative, the enrichment of Trim28 and deposition of H3K9me3 at the DMRs may provide an interaction scaffold for Uhrf1, which in turn could serve to recruit or activate Dnmt1 at these regions to facilitate its targeted de novo activity at IAPEz-ints (Fig. 5c). However, follow up studies are needed to directly determine this effect."

And page 10 (Discussion):

"Mechanistically, we establish the dependence on its co-factor Uhrf1 for the *de novo* activity and show an association with H3K9me3 and TRIM28 enriched genomic regions. While further study is needed, zinc finger proteins are known to recruit TRIM28 to ERV retrotransposons, which could serve to enrich both Uhrf1 and H3K9 tri-methyltransferases like Setdb1 at these regions. Uhrf1 binding to Trim28 or H3K9me3

at the IAPs could then potentially increase the retention time of Dnmt1 at these locations allowing for increased de novo and maintenance activity.”

Minor Comments:

1. Why is the color scale range for Uhrf1 binding different from Figure 5C (range = [0-2]) versus Figure ED5b (range = [0-0.25])? The same is true for Trim28, Figure 5c versus Figure ED5e. We thank the reviewer for pointing this out. We will clarify in the figure legends, which data were used. In the EDs the read coverage for each sample was displayed to provide information on the quality and reproducibility across samples, while the heatmap in the main figures shows the pooled replicates that were normalized by the sequenced input. Specifically, we propose to add the following to the figure legends:

Figure 5c: “The signal is displayed as noise subtracted read enrichment for pooled replicates.”

ED Figure 5b, e: “The signal is displayed as fragment pile up per million reads per replicate.”

2. Check the data in Figure ED3g --- the color scheme is swapped from Figure ED3d, and the data in the current form demonstrates that methylation in TKOL at both the remaining DMRs (from Nanopore analysis) and excluded DMRs is elevated compared to DKO0 --- this is in contrast to the rest of the data presented in the manuscript. Indeed, the data does not match the interpretation that is written in the figure legend: “Both subsets show comparable methylation levels when measured by WGBS, indicating that they don’t represent a low or high methylated outlier set.” The data does not support this statement as currently presented. Additionally, the y-axis is labeled “Men methylation” instead of “Mean methylation”.

We thank the reviewer for catching this. In fact, the x-axis labels not the colors were swapped. Thus, the data shown are in line with all other data and the legends. We will of course correct the x-axis labels.

3. Color scale in Figure 6d differs from how Mean methylation is presented elsewhere in the manuscript (e.g. Figure 5c). Indeed, the blue coloring appears to be below 0.0, and given the range of methylation data [0-1], the blue color should not appear at all in the heatmaps.

We thank the reviewer for pointing this out, as it might be confusing to others, too. The software packages used for visualization set the ticks for 0 and 1 at technically most effective positions, while the colors fill the whole width of the bar. Therefore, they are not at the right positions and were already corrected elsewhere in the manuscript.

We will adjust them here as well, to point to the correct colors for the most extreme methylation levels presented:

Minor change to Figure 6d in the Illustrator file.

4. In Figure 3d, the data for TKOL (the control) also needs to be presented in this way in the figure to show the change in methylation at DKO0 P15 at individual loci. Potentially (and as indicated by the hairpin bisulfite sequencing data), TKOL does have loci that are methylated, and these loci are masked/diluted in the average profiles presented in Figure 3c.

We agree with the reviewer, that it might be helpful to also show the TKO_L in this figure. However, we would also like to point out, that true positive methylation found in the TKO_L cells not necessarily means that this represents a subclone but rather *de novo* events already occurring during TKO_L expansion.

Decision Letter, second revision:

28th Apr 2021

Dear Alex,

Thank you for submitting your revised manuscript "Dnmt1 has de novo activity targeted to transposable elements" (NSMB-A44285B). As we had discussed, reviewer #2 found that the clarifications and additional analysis provided in your draft rebuttal satisfy the remaining concerns. Now that these have been included in the manuscript, we'll be happy in principle to publish it in Nature Structural & Molecular Biology, pending minor revisions to comply with our editorial and formatting guidelines.

We have performed detailed checks on your paper and will send you a checklist detailing our editorial and formatting requirements shortly. We are currently finalizing the June issue of NSMB and I would be happy to reserve a space for your article. Due to tight production timelines, we would need to receive the final submission within one week (by May 5th), if possible.

Thank you again for your interest in Nature Structural & Molecular Biology Please do not hesitate to contact me if you have any questions.

With kind regards,
Anke

Anke Sparmann, PhD
Senior Editor
Nature Structural and Molecular Biology
ORCID 0000-0001-7695-2049

Final Decision Letter:

10th May 2021

Dear Alex,

We are happy to accept your revised paper "Dnmt1 has de novo activity targeted to transposable elements" for publication as an Article in Nature Structural & Molecular Biology.

Acceptance is conditional on the manuscript's not being published elsewhere and on there being no announcement of this work to the newspapers, magazines, radio or television until the publication date in Nature Structural & Molecular Biology.

Before the manuscript is sent to the printers, we shall make any detailed changes in the text that may be necessary either to make it conform with house style or to make it intelligible to a wider readership. If the changes are extensive, we will ask for your approval before the manuscript is laid out for production. Once your manuscript is typeset you will receive a link to your electronic proof via email within 20 working days, with a request to make any corrections within 48 hours. Please read proofs with great care to make sure that the sense has not been altered. If you have queries at any point during the production process then please contact the production team at rjsproduction@springernature.com. Once your paper has been scheduled for online publication, the Nature press office will be in touch to confirm the details.

Please note that due to tight production schedules, proofs should be returned as quickly as possible to avoid delaying publication. If you anticipate any limitations to your availability over the next 2-4 weeks (such as vacation or traveling to conferences, etc.), please e-mail rjsproduction@springernature.com as soon as possible. Please provide specific dates that you will be unavailable and provide detailed contact information for an alternate corresponding author if necessary.

To assist our authors in disseminating their research to the broader community, our SharedIt initiative provides all co-authors with the ability to generate a unique shareable link that will allow anyone (with or without a subscription) to read the published article. Recipients of the link with a subscription will also be able to download and print the PDF.

As soon as your article is published, you can generate your shareable link by entering the DOI of your article here: <a href="http://authors.springernature.com/share">http://authors.springernature.com/share<a>. Corresponding authors will also receive an automated email with the shareable link

Note the policy of the journal on data deposition: http://www.nature.com/authors/policies/availability.html.

Your paper will be published online soon after we receive proof corrections and will appear in print in the next available issue. You can find out your date of online publication by contacting the production team shortly after sending your proof corrections. Content is published online weekly on Mondays and Thursdays, and the embargo is set at 16:00 London time (GMT)/11:00 am US Eastern time (EST) on the day of publication. Now is the time to inform your Public Relations or Press Office about your paper, as they might be interested in promoting its publication. This will allow them time to prepare an accurate and satisfactory press release. Include your manuscript tracking number (NSMB-A44285C) and our journal name, which they will need when they contact our press office.

About one week before your paper is published online, we shall be distributing a press release to news

organizations worldwide, which may very well include details of your work. We are happy for your institution or funding agency to prepare its own press release, but it must mention the embargo date and Nature Structural & Molecular Biology. If you or your Press Office have any enquiries in the meantime, please contact press@nature.com.

You can now use a single sign-on for all your accounts, view the status of all your manuscript submissions and reviews, access usage statistics for your published articles and download a record of your refereeing activity for the Nature journals.

If you have not already done so, we strongly recommend that you upload the step-by-step protocols used in this manuscript to the Protocol Exchange. Protocol Exchange is an open online resource that allows researchers to share their detailed experimental know-how. All uploaded protocols are made freely available, assigned DOIs for ease of citation and fully searchable through nature.com. Protocols can be linked to any publications in which they are used and will be linked to from your article. You can also establish a dedicated page to collect all your lab Protocols. By uploading your Protocols to Protocol Exchange, you are enabling researchers to more readily reproduce or adapt the methodology you use, as well as increasing the visibility of your protocols and papers. Upload your Protocols at www.nature.com/protocolexchange/. Further information can be found at www.nature.com/protocolexchange/about.

An online order form for reprints of your paper is available at https://www.nature.com/reprints/author-reprints.html. Please let your coauthors and your institutions' public affairs office know that they are also welcome to order reprints by this method.

Please note that <i>Nature Structural & Molecular Biology</i> is a Transformative Journal (TJ). Authors may publish their research with us through the traditional subscription access route or make their paper immediately open access through payment of an article-processing charge (APC). Authors will not be required to make a final decision about access to their article until it has been accepted. Find out more about Transformative Journals

<B>Authors may need to take specific actions to achieve compliance with funder and institutional open access mandates. For submissions from January 2021, if your research is supported by a funder that requires immediate open access (e.g. according to Plan S principles) then you should select the gold OA route, and we will direct you to the compliant route where possible. For authors selecting the subscription publication route our standard licensing terms will need to be accepted, including our self-archiving policies. Those standard licensing terms will supersede any other terms that the author or any third party may assert apply to any version of the manuscript.

In approximately 10 business days you will receive an email with a link to choose the appropriate publishing options for your paper and our Author Services team will be in touch regarding any additional information that may be required.

You will not receive your proofs until the publishing agreement has been received through our system.

If you have any questions about our publishing options, costs, Open Access requirements, or our legal forms, please contact ASJournals@springernature.com

With kind regards,
Anke

Anke Sparmann, PhD
Senior Editor
Nature Structural and Molecular Biology
ORCID 0000-0001-7695-2049

Click here if you would like to recommend Nature Structural & Molecular Biology to your librarian:
http://www.nature.com/subscriptions/recommend.html#forms
